# Global Burden of Bacterial Skin Diseases: A Systematic Analysis Combined With Sociodemographic Index, 1990–2019

**DOI:** 10.3389/fmed.2022.861115

**Published:** 2022-04-25

**Authors:** Yi Xue, Jie Zhou, Bei-Ni Xu, Yue Li, Wu Bao, Xia Lin Cheng, Yan He, Chun Peng Xu, Jun Ren, Ya rong Zheng, Chi Yu Jia

**Affiliations:** ^1^Department of Burns and Plastic & Wound Repair Surgery, Xiang'an Hospital of Xiamen University, School of Medicine, Xiamen University, Xiamen, China; ^2^School of Medicine, Xiamen University, Xiamen, China; ^3^Department of Clinical Laboratory, Shanghai Ninth People's Hospital, Shanghai Jiaotong University School of Medicine, Shanghai, China; ^4^Division of Plastic Surgery, Siming Branch of the First Affiliated Hospital of Xiamen University, Xiamen, China; ^5^Department of Dermatology, Zhongshan Hospital Xiamen University, Xiamen, China; ^6^Division of Plastic Surgery, Zhongshan Hospital Xiamen University, Xiamen, China

**Keywords:** bacterial skin diseases, cellulitis, pyoderma, sociodemographic index, disability-adjusted life years, global burden

## Abstract

**Background:**

The latest incidence and disability-adjusted life-years (DALYs) of major bacterial skin diseases (BSD) and their relationship with socioeconomic are not readily available.

**Objective:**

Describe the global age-standardized incidence and DALYs rates of BSD and analyze their relationship with socioeconomic.

**Methods:**

All data were obtained from Global Burden of Disease (GBD) 2019 database. The correlation between BSD and socioeconomic development status was analyzed.

**Results:**

The age-standardized incidence and age-standardized DALYs rate of BSD are: 169.72 million [165.28–175.44] and 0.41 million [0.33–0.48]. Of the two main BSD, pyoderma cause significantly much heavier burden than cellulitis. The change of age-standardized incidence (7.38% [7.06–7.67]) and DALYs (−10.27% [−25.65 to 25.45]) rate of BSD presented an upward or downward trend from 1990 to 2019. The highest burden was in the low-middle sociodemographic index (SDI) area while the area with the lowest burden was recorded in the high-middle SDI area in 2019.

**Limitations:**

GBD 2019 data of BSD are derived from estimation and mathematical modeling.

**Conclusion:**

The burden of BSD is related to socioeconomic development status. The results based on GBD2019 data may benefit policymakers in guiding priority-setting decisions for the global burden of BSD.

## Introduction

Skin diseases are common and have a great impact on patients' quality of life (1). More than 1000 skin or skin-related diseases are listed above according to the 10 categories of human diseases in the International Classification of Diseases (2). Strikingly, skin and subcutaneous diseases ranked 18th in the global DALYs disease burden ranking (GBD2013), while skin diseases were the fourth leading cause of disability globally (3).

Amongst skin diseases, bacterial skin infections, which mainly include cellulitis and pyoderma, account for a great proportion (4). However, despite the profound impact of bacterial skin diseases on the global disease burden, there appears to be a lack of commensurate attention globally. With the aim of improving health systems and eliminate disparities, the Global Burden of Disease (GBD) database 2019 incorporates risk factors and other parameters, and covers more than 200 countries and regions from 1990 to 2019 (5).

We combined the latest data from GBD 2019 to systematically analyse the relationship between bacterial skin disease and their relationship with socioeconomic. Changes in the burden of bacterial skin disease from 1990 to 2019 and potential impact of such changes were intensively investigated, implications of which may facilitate in formulating intervention strategies on a global scale.

## Methods

### Overview and Data Sources

About 369 diseases and injuries in 204 countries or regions, as well as behavioral (more than 80), occupational, environmental and other risk factors are included in the GBD2019 database (6). Age-standardized incidence and DALYs rates for bacterial skin diseases were calculated using GBD 2019 (http://ghdx.healthdata.org/gbd-2019).

### DALYs

DALYs stands for the loss of 1 year of healthy life, therefore the burden of disease can be estimated based on DALYs. DALYs includes the years of life lost (YLL) to a disease, and years of disability (YLD) is estimated for each reason, age group, regions, sex and year (7, 8).

### SDI

As a comprehensive indicator, SDI represents the geometric mean of the normalized value of the regional per capita income, the number of years of education of those 15 years old and above, and the total fertility rate (TFR) of women under 25, which is representative of the development of society and the population. The overall status of socioeconomic development can be stratified by the SDI. Specifically, GBD 2019 divides countries into five categories based on SDI indicators: high SDI, high-middle SDI, middle SDI, low-middle SDI and low SDI (9).

### Uncertainty Analysis

This study was based on the assumptions that all the date (incidence or DALYs) were log-normally distributed. The 95% uncertainty interval was the 2.5th and 97.5th percentile. Prism 9.0.0 software and Adobe Photoshop 2021 were used for related graphics and images.

## Results

### Burden of Major Types of Bacterial Skin Diseases

**Figure 1:**
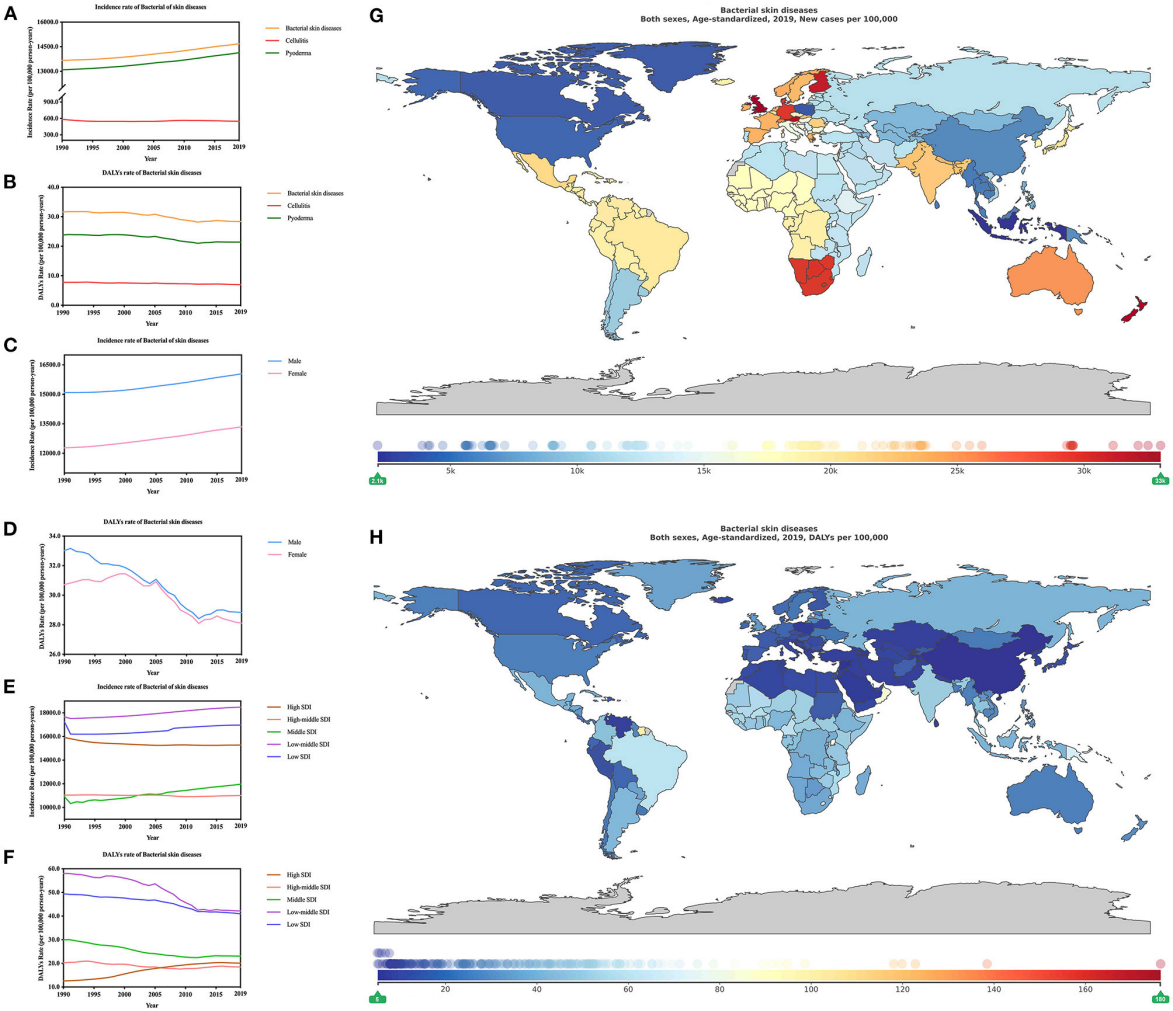
Burden of major bacterial skin diseases for 204 countries and territories. Age-standardized incidence and DALYs rates of major bacterial skin diseases **(A,B)** by type; **(C,D)** by sex; **(E,F)** by SDI per 100,000 people globally, 1990–2019. The distribution of age-standardized incidence **(G)** and DALYs **(H)** rates of bacterial skin diseases per 100,000 population globally, in 2019.

Skin disease, especially bacterial skin infections (including pyoderma and cellulitis), represent a significant global burden of disease (10, 11). Collectively, the age-standardized incidence of bacterial skin disease increased marginally from 1990 to 2019 (7.38% [7.06–7.67], Table 1), whereas the DALYs showed a slightly decreasing trend (−10.27% [−25.65 to 25.45], Table 1). The age-standardized incidence rates of pyoderma remained consistently far beyond cellulitis (Figures 1A,B; Supplementary Table 1). In 2019, age-standardized incidence rates were 146.84 million [143.21–151.34] for pyoderma and 0.28 million [0.21–0.34] for cellulitis (Supplementary Table 1).

**Table 1:** Age-standardized incidence rate of major bacterial skin diseases in 2019, and change of age-standardized incidence rate for both sexes (1990–2010 and 2010–2019).

**Group**	**Bacterial skin diseases**		**Cellulitis**		**Pyoderma**	
	**Number of incident cases, 2019**	**Annualised rate of change of age-standardized incidence(1990–2019) (%)**	**Number of incidence cases, 2019**	**Annualised rate of change of age-standardized incidence (1990–2019) (%)**	**Number of incident cases, 2019**	**Annualised rate of change of age-standardized incidence (1990–2019) (%)**
Global	14684.30(14321.55 to 15134.03)	7.38(7.06 to 7.67)	548.42(515.82 to 580.15)	−6.15(−6.76 to−5.55)	14135.87(13766.18 to 14585.22)	7.99(7.64 to 8.30)
High SDI	15276.52(14896.68 to 15673.53)	−4.06(−4.56 to−3.56)	1611.70(1524.39 to 1695.94)	6.07(4.92 to 7.19)	13664.82(13279.56 to 14062.69)	−5.13(−5.69 to −4.59)
High-middle SDI	11010.68(10736.91 to 11356.88)	−0.29(−0.69 to 0.12)	393.24(368.49 to 417.64)	−10.51(−11.32 to−9.70)	10617.45(10343.26 to 10966.78)	0.13(−0.29 to 0.56)
Middle SDI	11974.15(11676.49 to 12338.95)	9.75(9.36 to 10.17)	326.80(304.80 to 349.61)	8.11(7.50 to 8.73)	11647.34(11348.28 to 12014.66)	9.80(9.40 to 10.22)
Low-middle SDI	18489.09(18017.94 to 19059.53)	4.69(4.09 to 5.26)	380.41(355.40 to 406.00)	4.79(4.16 to 5.40)	18108.69(17636.38 to 18682.22)	4.69(4.08 to 5.27)
Low SDI	16972.32(16528.17 to 17544.18)	−1.60(−2.15 to−1.06)	336.14(313.60 to 358.93)	−1.61(−2.26 to −0.96)	16636.19(16189.18 to 17199.82)	−1.60(−2.16 to −1.05)
High-income	15895.36(15486.56 to 16315.84)	−2.87(−3.36 to−2.39)	1563.47(1477.34 to 1647.42)	9.14(8.05 to 10.22)	14331.89(13933.98 to 14762.22)	−4.02(−4.53 to−3.53)
High-income North America	4637.02(4467.79 to 4788.51)	3.81(2.90 to 4.75)	2952.51(2796.93 to 3102.02)	5.10(3.70 to 6.49)	1684.51(1640.28 to 1736.31)	1.64(0.88 to 2.45)
Australia	24964.52(24183.89 to 25818.02)	1.27(−0.69 to 3.47)	1926.39(1811.22 to 2038.34)	6.76(4.18 to 9.76)	23038.13(22257.84 to 23881.56)	0.84(-1.26 to 3.21)
High-income Asia Pacific	19499.45(18842.85 to 20169.40)	4.03(3.05 to 4.99)	1132.01(1052.80 to 1211.39)	4.00(2.66 to 5.30)	18367.44(17740.12 to 19052.07)	4.03(3.02 to 5.06)
Western Europe	25215.11(24510.93 to 25993.65)	2.93(2.23 to 3.63)	738.30(694.15 to 783.93)	8.42(7.39 to 9.37)	24476.82(23767.14 to 25264.24)	2.78(2.06 to 3.48)
Southern Latin America	10562.41(10229.30 to 10934.31)	1.59(0.16 to 3.11)	528.55(492.82 to 562.19)	7.23(5.02 to 9.40)	10033.86(9694.68 to 10408.39)	1.31(−0.18 to 2.89)
Central Europe, Eastern Europe, and Central Asia	11774.14(11480.19 to 12133.24)	−2.53(−2.93 to−2.15)	751.09(703.88 to 798.13)	0.62(-0.04 to 1.36)	11023.05(10733.59 to 11388.25)	−2.73(−3.14 to−2.35)
Eastern Europe	12069.63(11757.23 to 12447.59)	1.83(1.34 to 2.32)	1076.09(1007.04 to 1142.78)	6.57(5.75 to 7.42)	10993.54(10686.89 to 11369.00)	1.39(0.86 to 1.92)
Central Europe	13412.22(13085.29 to 13795.12)	−5.72(−6.45 to−5.00)	440.54(413.74 to 465.71)	−2.94(−4.24 to −1.60)	12971.68(12644.57 to 13349.88)	−5.82(−6.57 to −5.07)
Central Asia	9050.75(8755.44 to 9401.96)	1.70(0.71 to 2.76)	407.73(379.93 to 435.17)	3.66(2.49 to 4.80)	8643.02(8345.84 to 9004.76)	1.61(0.57 to 2.70)
Latin America and Caribbean	20043.98(19498.86 to 20670.31)	2.18(1.74 to 2.59)	691.79(638.43 to 742.88)	2.71(1.95 to 3.51)	19352.18(18806.80 to 19964.95)	2.16(1.70 to 2.59)
Central Latin America	20371.54(19800.48 to 21042.91)	2.06(1.44 to 2.65)	804.15(742.57 to 865.95)	3.96(3.01 to 4.95)	19567.39(19003.77 to 20222.79)	1.99(1.34 to 2.60)
Andean Latin America	19051.68(18524.39 to 19647.38)	2.11(0.65 to 3.56)	530.75(490.87 to 570.55)	6.44(4.39 to 8.61)	18520.93(17990.10 to 19113.41)	1.99(0.48 to 3.46)
Caribbean	18856.09(18201.68 to 19544.92)	1.59(0.44 to 2.65)	548.04(508.15 to 587.28)	4.04(2.34 to 5.73)	18308.05(17661.07 to 18991.17)	1.52(0.34 to 2.62)
Tropical Latin America	19845.47(19312.68 to 20456.08)	1.68(1.04 to 2.32)	647.42(600.70 to 693.67)	0.16(-1.03 to 1.51)	19198.05(18672.27 to 19809.57)	1.73(1.07 to 2.42)
Southeast Asia, East Asia, and Oceania	6067.76(5892.37 to 6262.81)	−1.57(-2.06 to−1.10)	167.38(155.25 to 179.50)	1.75(0.83 to 2.66)	5900.38(5726.45 to 6089.79)	−1.66(-2.16 to−1.18)
East Asia	6308.69(6132.45 to 6518.26)	−4.05(−4.65 to−3.43)	87.21(80.44 to 93.68)	−21.07(−22.25 to−19.95)	6221.48(6043.85 to 6425.97)	−3.75(−4.37 to−3.13)
Southeast Asia	5544.70(5371.71 to 5722.27)	9.30(8.41 to 10.14)	337.55(314.14 to 360.08)	8.08(7.27 to 8.81)	5207.15(5039.06 to 5389.12)	9.38(8.45 to 10.27)
Oceania	6561.95(6328.33 to 6813.54)	3.24(1.48 to 4.91)	209.56(194.58 to 223.67)	3.76(1.84 to 5.90)	6352.38(6124.11 to 6604.55)	3.22(1.43 to 4.95)
North Africa and Middle East	12492.96(12114.34 to 12941.14)	1.08(0.39 to 1.70)	313.73(292.81 to 335.37)	1.93(1.24 to 2.67)	12179.23(11791.83 to 12633.34)	1.06(0.36 to 1.71)
South Asia	22188.31(21634.46 to 22888.99)	5.53(4.82 to 6.17)	396.59(370.78 to 423.75)	7.63(6.91 to 8.32)	21791.72(21240.04 to 22499.46)	5.49(4.77 to 6.15)
Sub-Saharan Africa	17009.82(16532.26 to 17589.09)	−0.05(−0.39 to 0.29)	369.94(345.87 to 395.15)	−0.17(−0.61 to 0.28)	16639.88(16157.34 to 17214.90)	−0.05(−0.39 to 0.30)
Southern Sub-Saharan Africa	29473.69(28595.32 to 30423.06)	1.54(0.81 to 2.33)	691.61(645.90 to 734.71)	2.67(1.75 to 3.64)	28782.08(27925.79 to 29739.62)	1.51(0.77 to 2.33)
Western Sub-Saharan Africa	17604.18(17083.44 to 18242.78)	2.00(1.56 to 2.41)	393.94(367.42 to 421.43)	1.23(0.72 to 1.77)	17210.25(16685.92 to 17855.90)	2.02(1.57 to 2.44)
Eastern Sub-Saharan Africa	13026.00(12640.03 to 13421.44)	2.49(1.95 to 3.04)	258.59(240.49 to 276.82)	1.98(1.26 to 2.72)	12767.41(12383.60 to 13168.20)	2.50(1.95 to 3.05)
Central Sub-Saharan Africa	18944.63(18292.92 to 19701.23)	2.51(0.91 to 4.11)	391.92(364.94 to 418.69)	1.87(−0.08 to 3.76)	18552.71(17901.08 to 19318.79)	2.53(0.88 to 4.16)

The incidence of bacterial skin disease showed an increasing trend (both sex, 1990–2019) for, as for DALYs rates showed a dramatically decreasing pattern, especially from 2000 to 2012. Male prevalence over female remained from 1990 to 2019. In 2019, age-standardized incidence rate was 160.48 million [156.44–165.45] for males, while it was 133.54 million [130.24–137.58] for females. The age-standardized DALYs rate was 0.29 million [0.19–0.37] for males, whereas it was slightly lower for females, which was 0.28 million [0.22–0.34] (Figures 1C,D; Supplementary Table 2). As for the change of age-standardized incidence rate of bacterial skin diseases?the increasing trend was much greater in females 8.76% [8.36–9.16] than males 6.34% [5.94–6.73]. Interestingly, the change of age-standardized DALYs rate of bacterial skin diseases depicted a contrary trend, which showed that the decrease was more obvious in males (−12.70% [−31.49 to 6.44]) compared with that in females (−8.45% [−28.18 to 10.44] (Supplementary Table 3).

Further, the relationship between the global burden of bacterial skin diseases and socioeconomic status was explored. In 2019, low-middle SDI demonstrated the highest burden for the age-standardized incidence rate of bacterial skin diseases, followed by low SDI, high SDI, middle SDI, and high-middle SDI (Figures 1E,F, Supplementary Table 4). As for age-standardized DALYs, low-middle and high-middle SDI locations recorded the highest and lowest burden, respectively. In low SDI locations, as for incidence rates were 169.72 million [165.28–175.44] and as for DALYs rates were 0.41 million [0.33–0.48]; in high-middle locations, as for incidence rates were 110.10 million [107.37–113.57] and as for DALYs rates 0.18 million [0.13–0.24].

We analyzed the incidence (Table 1) and DALYs (Table 2) rate of bacterial skin diseases in 21 region-specific and country-specific locations. Our findings include the following: Southern Sub-Saharan Africa (294.74 million [285.95–304.23]) and high-income North America (46.37 million [44.68–47.89]), respectively, had the heaviest and lowest burden of incidence. However, in the value of DALYs rates, Oceania [73.28 [46.61–101.48] (per 1,000 person-years)] and East Asia (5.91 [4.67–8.14] [per 1000 person-years)] recorded the heaviest and lowest burden.

**Table 2:** Age–standardized DALYs rate of major bacterial skin diseases in 2019, and change of age–standardized DALYs rate for both sexes (1990–2010 and 2010–2019).

**Group**	**Bacterial skin diseases**		**Cellulitis**		**Pyoderma**	
	**Number of DALYs, 2019**	**Annualised rate of change of age-standardized DALYs(1990–2019) (%)**	**Number of DALYs, 2019**	**Annualised rate of change of age-standardized DALYs(1990–2019) (%)**	**Number of DALYs, 2019**	**Annualised rate of change of age-standardized DALYs(1990–2019) (%)**
Global	28.39(21.35 to 34.10)	−10.27(−25.65 to 2.54)	6.96(4.75 to 8.35)	−10.52(−36.46 to 15.42)	21.43(16.18 to 26.20)	−10.18(−27.51 to 4.89)
High SDI	20.09(13.22 to 27.02)	60.09(10.49 to 92.06)	10.55(6.83 to 13.57)	53.99(2.47 to 82.34)	9.54(6.13 to 14.11)	67.42(16.98 to 117.29)
High–middle SDI	18.48(12.90 to 23.84)	−8.59(−23.19 to 2.74)	6.82(4.43 to 9.33)	−8.83(−32.55 to 8.08)	11.66(8.30 to 15.55)	−8.44(−24.42 to 4.79)
Middle SDI	23.06(17.93 to 27.64)	−23.07(−32.19 to −9.18)	4.90(3.47 to 5.81)	−26.56(−42.04 to −0.92)	18.17(13.99 to 22.37)	−22.07(−33.42 to −5.00)
Low-middle SDI	42.16(28.74 to 50.30)	−27.38(−41.70 to −9.14)	5.82(3.71 to 7.42)	−35.18(−53.06 to −6.26)	36.34(24.88 to 43.95)	−25.96(−42.38 to −6.68)
Low SDI	40.95(32.97 to 47.96)	−17.07(−33.37 to −3.08)	6.60(3.19 to 9.44)	−27.46(−52.26 to 9.00)	34.36(28.47 to 41.47)	−14.72(−33.69 to 1.45)
High-income	20.34(13.26 to 27.56)	69.57(14.93 to 107.13)	10.12(6.66 to 12.97)	57.53(4.49 to 87.64)	10.22(6.28 to 15.09)	83.45(22.56 to 147.97)
High-income North America	25.30(16.87 to 34.92)	80.34(23.22 to 112.72)	17.40(11.42 to 22.26)	53.98(6.32 to 82.54)	7.90(4.28 to 12.35)	189.64(73.58 to 248.27)
Australia	26.18(15.81 to 34.83)	67.19(3.33 to 112.70)	14.33(7.73 to 18.08)	87.83(−7.64 to 150.84)	11.85(7.34 to 18.38)	47.59(11.93 to 88.78)
High-income Asia Pacific	11.98(8.33 to 17.64)	25.42(−0.82 to 43.22)	5.98(4.36 to 8.16)	31.20(−8.42 to 53.38)	6.00(3.47 to 10.47)	20.14(6.09 to 42.16)
Western Europe	18.65(11.77 to 26.33)	66.29(10.08 to 110.20)	6.03(3.60 to 7.65)	62.60(−7.05 to 100.38)	12.62(7.73 to 19.49)	68.12(17.47 to 125.37)
Southern Latin America	36.74(20.58 to 51.05)	155.78(65.62 to 234.98)	10.51(6.48 to 15.41)	55.66(17.00 to 89.96)	26.23(13.62 to 36.20)	244.65(88.76 to 398.00)
Central Europe, Eastern Europe, and Central Asia	23.90(14.91 to 33.28)	32.95(−2.55 to 58.30)	14.48(8.58 to 20.66)	40.69(−5.64 to 72.90)	9.42(5.86 to 13.71)	22.59(−0.92 to 43.55)
Eastern Europe	36.45(21.98 to 51.89)	50.21(5.70 to 84.13)	24.96(14.31 to 36.42)	63.66(3.19 to 107.72)	11.50(6.68 to 16.93)	27.49(3.90 to 50.72)
Central Europe	10.26(6.92 to 14.19)	0.80(−19.91 to 17.63)	2.64(1.96 to 3.38)	−34.41(−44.85 to −17.06)	7.62(4.66 to 11.30)	23.80(−15.21 to 58.80)
Central Asia	9.45(7.02 to 13.13)	9.12(−1.95 to 24.23)	3.35(2.40 to 4.78)	3.69(−9.73 to 21.67)	6.10(4.40 to 9.08)	12.35(−0.06 to 29.52)
Latin America and Caribbean	46.06(27.91 to 61.52)	61.24(−0.40 to 97.32)	10.84(6.30 to 14.83)	13.91(−26.91 to 40.65)	35.22(20.06 to 48.11)	84.89(13.56 to 131.21)
Central Latin America	38.33(23.45 to 55.68)	66.87(10.42 to 106.17)	11.85(6.74 to 17.05)	30.20(−13.29 to 57.88)	26.48(15.62 to 39.13)	90.94(25.47 to 155.03)
Andean Latin America	15.62(11.90 to 20.58)	−27.36(−48.16 to −3.93)	3.26(2.46 to 4.24)	−56.32(−71.90 to 1.76)	12.36(9.04 to 16.77)	−12.00(−47.97 to 33.43)
Caribbean	47.98(36.71 to 71.95)	30.60(−1.63 to 69.71)	12.58(8.11 to 21.42)	0.38(−23.42 to 35.45)	35.39(26.43 to 54.68)	46.25(3.72 to 92.58)
Tropical Latin America	61.55(31.92 to 78.92)	80.27(−4.57 to 130.35)	11.56(5.65 to 15.44)	16.21(−39.77 to 54.48)	49.99(24.78 to 65.46)	106.61(14.09 to 162.55)
Southeast Asia, East Asia, and Oceania	14.70(11.70 to 17.44)	−48.21(−55.39 to −29.59)	3.52(2.27 to 4.17)	−46.53(−59.12 to −22.78)	11.18(8.94 to 13.88)	−48.71(−56.99 to −26.39)
East Asia	5.91(4.67 to 8.14)	−75.82(−81.43 to −53.33)	1.38(1.07 to 1.91)	−75.21(−83.85 to −45.55)	4.53(3.39 to 6.73)	−76.00(−83.62 to −50.43)
Southeast Asia	38.32(27.42 to 45.40)	−8.21(−24.06 to 10.32)	9.47(5.41 to 11.39)	−8.64(−25.21 to 19.81)	28.85(21.70 to 36.05)	−8.06(−24.99 to 14.09)
Oceania	73.28(46.61 to 101.48)	9.23(−15.08 to 42.62)	11.43(4.66 to 19.90)	−25.86(−45.48 to 8.48)	61.85(39.91 to 87.24)	19.71(−10.14 to 58.93)
North Africa and Middle East	10.26(7.70 to 14.76)	−6.06(−17.83 to 9.09)	1.94(1.46 to 2.74)	−25.54(−40.77 to 12.10)	8.32(5.88 to 12.42)	0.03(−14.04 to 19.14)
South Asia	43.93(28.43 to 53.57)	−36.11(−49.87 to −15.86)	5.42(3.44 to 7.25)	−40.55(−57.44 to −12.47)	38.51(24.54 to 47.91)	−35.43(−50.68 to −14.56)
Sub-Saharan Africa	46.83(38.66 to 55.68)	−5.34(−20.55 to 10.59)	8.35(3.92 to 11.70)	−19.62(−44.77 to 15.26)	38.48(31.68 to 47.50)	−1.55(−19.69 to 20.22)
Southern Sub-Saharan Africa	40.77(32.92 to 50.43)	−5.76(−16.70 to 6.81)	13.17(8.49 to 16.28)	−8.54(−22.10 to 11.65)	27.60(21.28 to 36.33)	−4.37(−17.18 to 12.53)
Western Sub-Saharan Africa	49.32(39.57 to 60.39)	−0.06(−16.20 to 18.47)	3.81(1.93 to 5.34)	−22.80(−41.01 to 16.79)	45.50(36.33 to 56.15)	2.47(−15.14 to 24.09)
Eastern Sub-Saharan Africa	46.33(37.59 to 56.17)	−8.78(−32.29 to 13.87)	10.96(3.93 to 17.60)	−20.23(−55.08 to 34.45)	35.37(27.90 to 45.32)	−4.54(−33.63 to 27.71)
Central Sub-Saharan Africa	41.69(31.83 to 54.71)	−10.99(−32.14 to 17.51)	10.68(3.27 to 17.57)	−19.32(−47.89 to 43.53)	31.00(21.77 to 46.31)	−7.70(−33.42 to 27.36)

Change of age-standardized incidence (Supplementary Table 5) and DALYs (Supplementary Table 6) rate of bacterial skin diseases in 204 countries from 1990 to 2019 were explored. Regions with the most significant change in age-standardized incidence were Taiwan (Province of China) (9.53% [7.12–12.19]) and Poland (−35.94% [−35.65 to −32.29]); as for DALYs, Saint Vincent and the Grenadines (3.96 times [0.68 to 8.99]) and China mainland (−79.06% [−84.43 to −55.85%]). However, the regions with the greatest changes differed by approximately 15-fold in the age-standardized incidence of bacterial skin disease (United Kingdom, 330.18 million [321.50–339.55]) and least change in 2019 (China, 21.21 million [20.58–21.94]) (Figure 1G). Also, there was 34 times difference between regions with the biggest change of age-standardized DALYs rate of bacterial skin diseases (American Samoa, 1.76 million [1.25–2.29]) and the least (China, 5.16 [3.97–7.45]) [per 1,000 person-years)] (Figure 1H).

### Burden of Cellulitis

Cellulitis is a bacterial infection of the skin, and the main clinical features include poorly demarcated erythema, edema, warmth, and tenderness (12). Skin diseases accounted for 1.79% of the global disease burden in 2013. In the same period, cellulitis contributed 0.04% of total burden (13). In the United States, an estimated 14.5 million cases annually of cellulitis account for $3.7 billion in ambulatory care cost alone (12).

**Figure 2:**
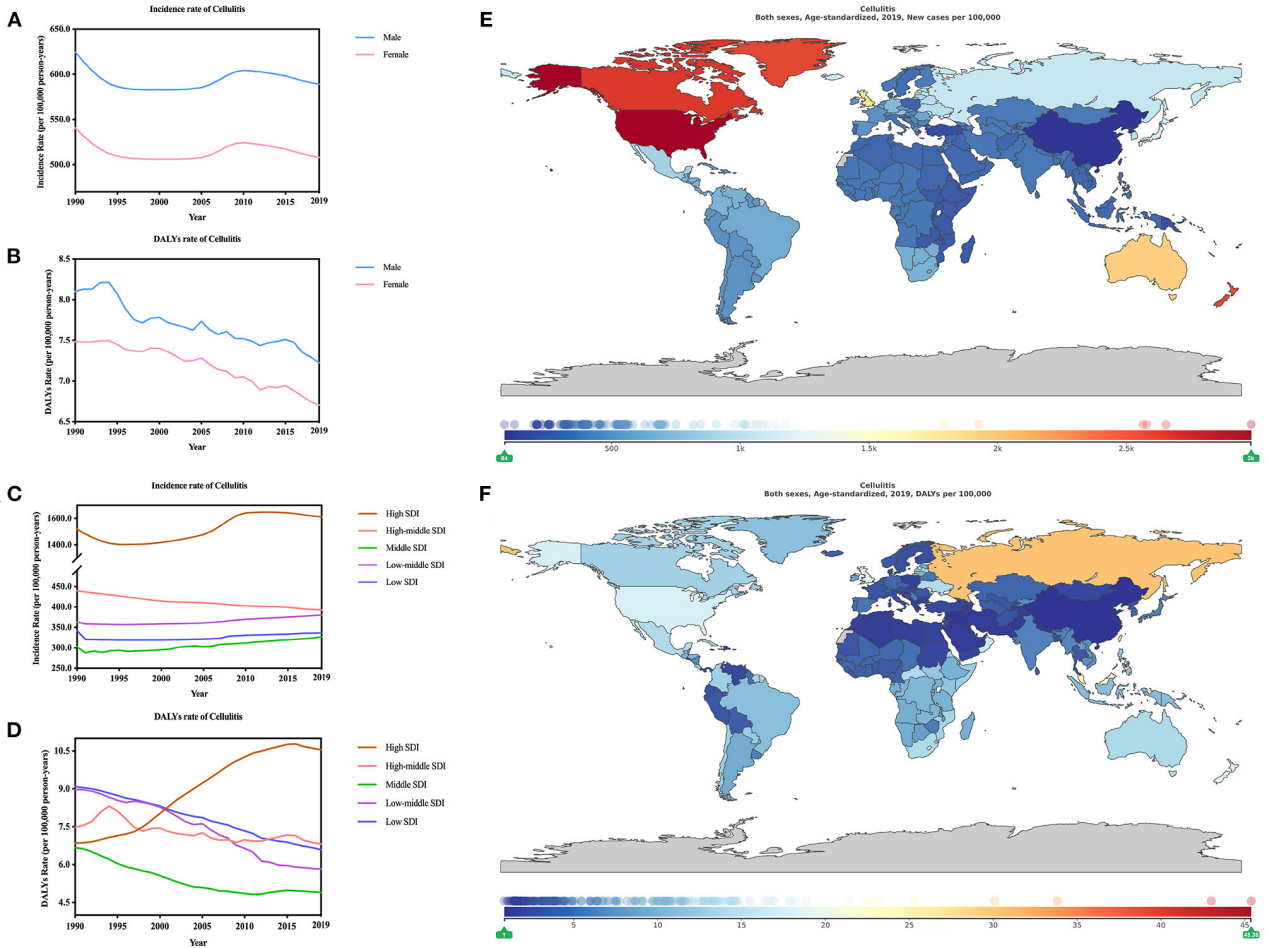
Burden of cellulitis for 204 countries and territories. Age-standardized incidence and DALYs rates of cellulitis **(A,B)** by sex; **(C,D)** by SDI per 100,000 population globally,1990–2019. The distribution of age-standardized incidence **(E)** and DALYs **(F)** rates of cellulitis per 100,000 population globally, in 2019.

In 2019, the incidence and DALYs rate of Cellulitis were 54.84 million [51.58–58.02] and 6.96 [4.75–8.35] (per 1,000 person-years) (Figures 1A,B; Supplementary Table 1). Interestingly, cellulitis depicted a sex gap the incidence and DALYs rates, which tended to have a male predominance over females. Age-standardized incidence was 5.89 million [5.54–6.23] for males, while it was 5.08 million [4.78–5.37]. As for DALYs rate, it was 7.22 [4.03–9.79] for males and 6.70 [4.18–8.12] (per 1,000 person-years) for females (Figures 2A,B; Supplementary Table 7).

According to the change of age-standardized incidence and DALYs rate of cellulitis from 1990 to 2019, analysis showed a decreasing trend (−6.15% [−6.76 to −5.55] (Table 1) and −10.52% [−36.46 to 15.42] (Table 2), respectively). At the same time change of age-standardized incidence rate of cellulitis was slighter for males (−5.70% [−6.32 to −5.10] than females (−6.06% [−6.75 to −5.32]), while contrarily the change of age-standardized DALYs rate of cellulitis was greater for male (−10.83% [−40.95 to 14.50]) than female (−10.42% [−40.40 to 20.21]) (Supplementary Table 3).

In 2019, as for incidence (or DALYs) rate of Cellulitis both indicated that the heaviest burden was high-SDI regions, with incidence of 16.12 million [15.24–16.96] and DALYs of 10.55 [6.83–13.57] (per 1,000 person-years). In contrast, high-middle SDI regions demonstrated the lowest burden with age-standardized incidence of 3.27million [3.05–3.50] and DALYs of 4.90 [3.47–5.81] (per 1,000 person-years) (Figures 2C,D; Supplementary Table 8). The lowest incidence of cellulitis incidence rates countries, such as East Asia (0.87million [0.80–0.94]), also had the lowest cellulitis DALY rates [1.38 [1.07–1.91] (per 1,000 person-years)]. The highest burden of age-standardized incidence was high-income North America (29.53 million [27.97–31.02]) while the highest burden of age-standardized DALYs was recorded in Eastern Europe [24.96 [14.31–36.42] (per 1,000 person-years)].

The change of age-standardized incidence and DALYs rates were further analyzed. The biggest change of age-standardized incidence (Supplementary Table 5) and DALYs (Supplementary Table 6) rate of cellulitis were recorded both in China with incidence of 26.27% [22.27–30.13] in Taiwan (Province of China) and −22.97% [−24.18 to −21.84] in Chinese mainland, and DALYs, Mauritius (2.26 times [0.06 to 4.77]) and China (−84.17% [−90.14 to −63.52%]). The highest age-standardized incidence (Figure 2E) rate of Cellulitis was United States of America 29.85 million [28.28–31.36] while the lowest was China 0.84 million [0.77–0.90] in 2019. As for the incidence, it displayed a huge gap (34 times) between these two regions, so was the gap in terms of age-standardized DALYs (Figure 2F) rate of Cellulitis. Bahrain recorded the highest DALYs [(45.38 [23.27–66.46]) (per 1,000 person-years)], 53 times of that of China, which recorded the lowest [(0.85 [0.65–1.23]) (per 1,000 person-years)] DALYs.

### Burden of Pyoderma

Pyoderma gangrenosumis a rare neutrophilic, autoinflammatory disorder, manifesting as a rapidly extending deep ulcer, and is often associated with a several immune-mediated diseases (14). Pyoderma gangrenosum, characterized by compromised borders and peripheral erythema, and rapidly developing painful skin ulcers, affects millions of people annually and has an average age of onset around 40 years of age (14). It results from impaired local defense mechanisms, which permits secondary bacterial invasion of the skin. According to a study from the GBD 2013, skin diseases contributed 1.79% to the global burden of diseases. Meanwhile pyoderma accounted for 0.05, which was 0.01% higher than cellulitis (13).

**Figure 3:**
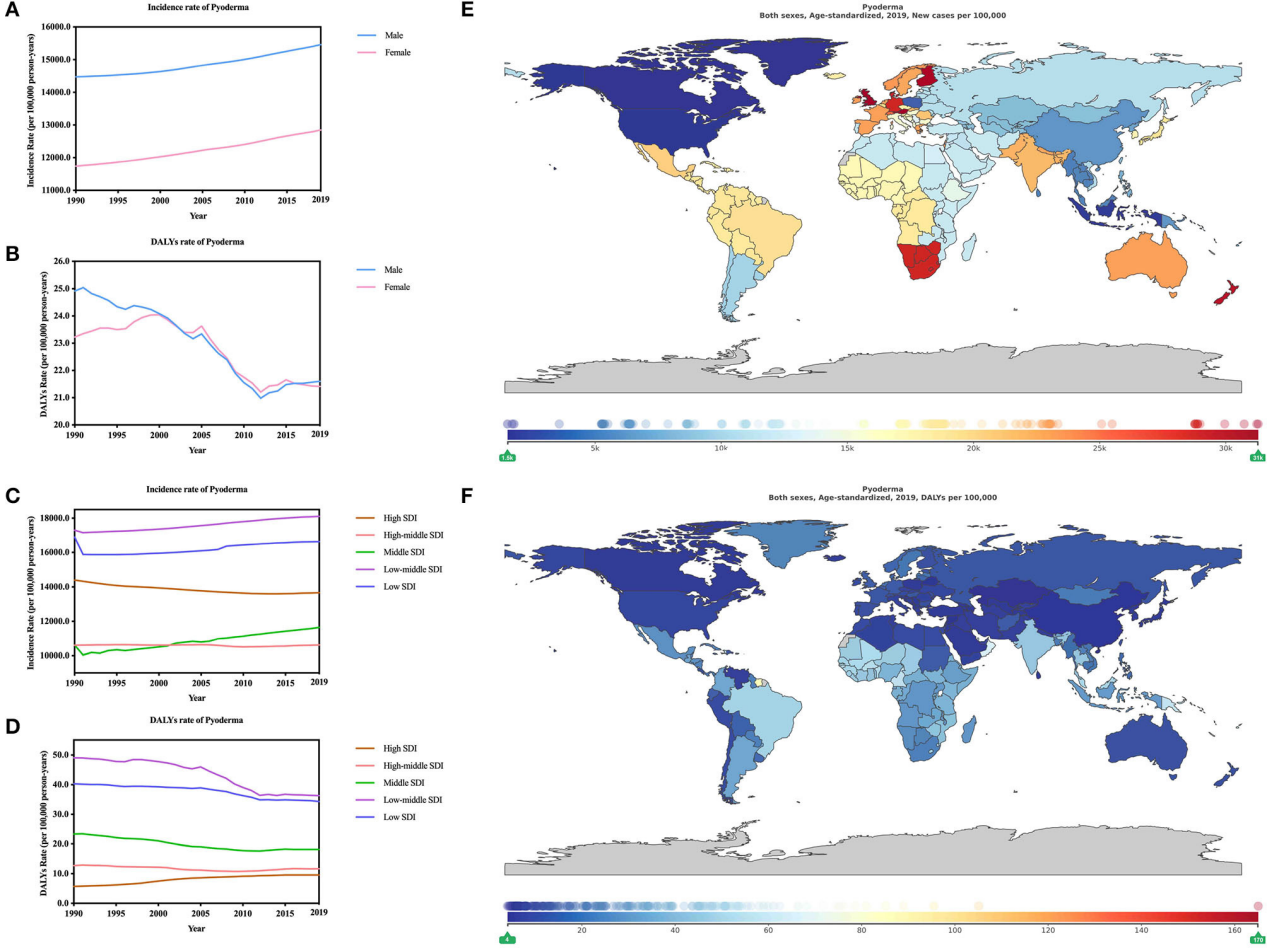
Burden of pyoderma for 204 countries and territories. Age-standardized incidence and DALYs rates of pyoderma **(A,B)** by sex; **(C,D)**, by SDI per 100,000 population globally,1990–2019. The distribution of age-standardized incidence **(E)** and DALYs **(F)** rates of pyoderma per 100,000 population globally, in 2019.

The age-standardized incidence and DALYs rate of Pyoderma was 141.36 million [137.66–145.85] and 21.43 [16.18–26.20] (per 1,000 person-years) (Figures 1A,B; Supplementary Table 1) in 2019. Like cellulitis, pyodermas also showed more males than females. Specifically, age-standardized incidence rate of pyodermas for males was 154.59 million [150.52–159.51] and 128.46 million [125.13–132.56] for females. Age-standardized DALYs rate was 21.60 [13.49–27.92] for males and 21.41 [16.94–26.36] (per 1,000 person-years) for females (Figures 3A,B; Supplementary Table 9). Significantly, the age-standardized incidence of of pyodermas from 1990 to 2019 increased (7.98% [7.64–8.30]) (Table 1) whereas the age-standardized DALYs showed a declining trend (−10.18% [−27.61 to 4.89]) (Table 2).

From 1990 to 2019, change of age-standardized incidence rate of pyodermas was much more obvious in females (9.44% [9.03 to 9.85]) than males (6.86% [6.43 to 7.26]). In addition, the change of DALYs rate of Pyodermas was greater in males (−13.31% [−35.05 to 12.00]) than females (−7.81% [−30.90 to 16.20]) (Supplementary Table 3).

In 2019, the incidence and DALYs rate of pyodermas both indicated that regions with the heaviest burn was low-middle SDI with incidence of 181.08 million [176.36–186.82] and DALYs of 36.34 [24.88–43.95] (per 1,000 person-years) (Figures 3C,D). Low SDI regions ranked the next. However, the High-middle SDI (106.17 million [103.43–109.67]) regions recorded the lowest age-standardized incidence rate of pyodermas while high SDI regions (9.54 [6.13–14.11]) (per 1,000 person-years) recorded the lowest age-standardized DALYs rate.

Through analyzation of the age-standardized incidence (Table 1) and DALYs (Table 2) rate of pyoderma, Southern Sub-Saharan Africa was found to be the heaviest burden areas (287.72 million [279.26–297.40]) while high-income North America (16.85 million [16.40–17.36]) had the least burden. Comparing these two regions, 17-fold gap could be identified. As for age-standardized DALY rates, the heaviest regions were Oceania [61.85 [39.91–87.24] (per 1,000 person-years)], 13-fold higher than East Asia [4.53 [3.39–6.73] (per 1,000 person-years)], which was the lowest DLAYs regions.

Further, we analyzed the change in 204 countries from 1990 to 2019. The change of age-standardized incidence (Supplementary Table 5) and DALYs (Supplementary Table 6) rate of pyoderma showed that Taiwan (Province of China) (9.15% [6.69–11.83]) was the region with the greatest deduction of burden while Poland (−35.10% [−36.92 to −33.39]) the biggest increase of burden, which was consistent with the change of age-standardized incidence of bacterial skin diseases. According to the change of DALYs rates, China (−77.63% [−84.92 to −52.71%]) declined the most while Saint Vincent and the Grenadines (5.43 times [0.77 to 17.11]) increased the most. In 2019, Austria (312.87 million [302.41–323.46]) showed the highest incidence (Figure 3E) rate of pyoderma, 20-fold higher than the region with the lowest incidence rate, which was Canada (15.25 million [14.79–15.77]) the lowest. The highest DALYs (Figure 3F) rate of pyoderma was American Samoa (1.65 million [1.09–2.21]) while China had the lowest (4.04 [2.47–6.76]) (per 1,000 person-years), 40-fold lower than the former one.

## Discussion

Bacterial skin diseases have detrimental effects on humans, ranging from physical incapacity to even death (15, 16). According to an analysis for the GBD 2017, bacterial skin diseases resulted in 76,000 deaths (48,700–95,600) in 2017 in total (10). However, the main findings of this study are as follows: (1) Age-standardized incidence of bacterial skin disease showed a marginal increase from 1990 to 2019 whereas the DALYs showed a slight decreasing trend. (2) Both age-standardized incidence and DALYs of pyoderma remained consistently higher than cellulitis. (3) In 2019, the top ranking of the age-standardized incidence and DALYs rates of bacterial skin disease were in the low-middle SDI area.

One major common bacterial skin disease is cellulitis. It is estimated that cellulitis contributed to 18,900 deaths (10,300–26,000) and age-standardized death rate of cellulitis was 0.2 (0.1 to 0.3) (per 100,000) from 2007–2017. Additionally, an upward trend could be identified in the percentage change of deaths of cellulitis (19.6% [9.8–28.2]) (10). Cellulitis often requires hospitalization for intravenous antibiotics, especially for patient who frequently have comorbid conditions or patients with severe manifestations, which therefore lead to considerable health care spending (17, 18). The cost of doctor visits for cellulitis (or erysipelas) continues to increase in the United States. And, from 2006 to 2015, the number of adults ($720–$1,680) and children ($939–$2,823) who visited the emergency department more than doubled and tripled, respectively (19). Additionally, misdiagnosis of lower extremity cellulitis also contribute to unnecessary patient morbidity (18).

Pyoderma is a major skin disease in this study, which contributed to even 3-fold more death than cellulitis, with 57,100 deaths (35,800–70,800). Age-standardized death rates of pyoderma were 0.8 (0.5 to 0.9) (per 100,000), much higher than that of cellulitis from 2007–2017. Additionally, an upward trend could be identified in the percentage change of deaths of cellulitis (19.6% [9.8–28.2]) and pyoderma (10.5% [3.2–19.0]) (10). The DALYs showed a surprisingly upward trend from 2012 to 2019 in this study. The incidence and DALYs rate of pyoderma was 141.36 million [137.66–145.85] and 21.43 [16.18–26.20] (per 1,000 person-years) in 2019. These findings indicated that prevention of pyoderma received little effects and pyoderma could contribute to the worsening of the global burden of bacterial skin disease.

As has been reported, potential reasons for the increased age-standardized incidence of bacterial skin disease are the lack of reliable microbiologic and laboratory diagnostics, which makes it difficult to differentiate from non-infectious mimics (20–23). The high prevalence of drug-resistant organisms and the increasing number of immunocompromised patients who are in transplantation and receive biologic immunosuppressives therapy are critical reasons for the complexity of treating these infections (24). A history of bacterial skin disease greatly increases the risk for subsequent episodes as well. Patients with comorbid conditions that predispose them to infection, particularly diabetes, are also at an upward trend of risk (25–28). Thus, prevention of bacterial skin diseases should focus on management of predisposing conditions.

This study still has some limitations. The GBD data are derived from estimation and mathematical modeling. The universality and accuracy of the research results could be influenced by inability to adjust confounding factors, such as patients, providers, and geographic levels. Although our research has some limitations, it can still provide a reference for studying the relationship between bacterial skin diseases and SDI to a certain extent.

To conclude, the findings in this report provide a systematic understanding of the burden of bacterial skin diseases from 1990 to 2019, which are valuable for the formulation of global bacterial skin disease prevention and control policies and the implementation of effective intervention measures to improve or reduce the burden of global bacterial skin disease.

## Data Availability Statement

The original contributions presented in the study are included in the article/Supplementary Material, further inquiries can be directed to the corresponding author/s.

## Author Contributions

CYJ, YrZ, JR, and YX: designed and conceived the study and critical revision of the manuscript for important intellectual content. YX, YL, and WB: data acquisition. JZ, XLC, and YH: analyzed the data. B-NX, CPX, and JR: drawn the picture. CYJ: study supervision. YrZ: administrative support. YX: finished the manuscript. All authors reviewed and validated the manuscript.

## Funding

This study was supported by the Starting Package of Xiang'an Hospital of Xiamen University (PM201809170010), Open project of Provincial Key Laboratory of Union Hospital Affiliated to Fujian Medical University in 2020 (Nos. XHZDSYS202004 and XHZDSYS202005).

## Conflict of Interest

The authors declare that the research was conducted in the absence of any commercial or financial relationships that could be construed as a potential conflict of interest.

## Publisher's Note

All claims expressed in this article are solely those of the authors and do not necessarily represent those of their affiliated organizations, or those of the publisher, the editors and the reviewers. Any product that may be evaluated in this article, or claim that may be made by its manufacturer, is not guaranteed or endorsed by the publisher.
