# Supplementary material for: Global Burden of Bacterial Skin Diseases: A Systematic Analysis Combined With Sociodemographic Index, 1990–2019
Source: Front Med (Lausanne). 2022 Apr 25;9:861115. doi: 10.3389/fmed.2022.861115 (PMC9084187; doi:10.3389/fmed.2022.861115)
Supplement: Supplementary file 1 [file Table_1.docx]

S1 Table Age-standardized incidence and DALYs of major bacterial skin diseases, both sexes, 1990-2019.

| **Group** | **Incidence** | | |  | **DALYs (Disability-Adjusted Life Years)** | | |
| --- | --- | --- | --- | --- | --- | --- | --- |
|  | **Bacterial skin diseases** | **Cellulitis** | **Pyoderma** |  | **Bacterial skin diseases** | **Cellulitis** | **Pyoderma** |
| **1990** | 13674.63(13336.66 to 14100.56) | 584.34(548.63 to 619.74) | 13090.29(12750.27 to 13506.00) |  | 31.63(23.79 to 38.92) | 7.77(5.18 to 10.78) | 23.86(17.18 to 31.11) |
| **1991** | 13681.10(13342.10 to 14106.90) | 574.15(539.39 to 608.62) | 13106.94(12769.47 to 13522.90) |  | 31.76(23.58 to 39.09) | 7.78(5.21 to 10.76) | 23.98(17.16 to 31.08) |
| **1992** | 13689.74(13349.96 to 14114.67) | 565.39(531.45 to 599.58) | 13124.35(12784.74 to 13540.40) |  | 31.71(23.54 to 38.68) | 7.79(5.21 to 10.72) | 23.93(17.05 to 30.64) |
| **1993** | 13700.52(13360.24 to 14124.25) | 558.21(524.56 to 591.71) | 13142.31(12802.63 to 13557.57) |  | 31.76(23.76 to 38.42) | 7.83(5.25 to 10.71) | 23.93(17.28 to 30.41) |
| **1994** | 13714.44(13373.81 to 14136.69) | 552.70(519.53 to 586.07) | 13161.73(12819.73 to 13581.31) |  | 31.70(23.77 to 38.36) | 7.84(5.29 to 10.66) | 23.87(17.20 to 30.36) |
| **1995** | 13732.20(13391.57 to 14155.33) | 549.48(516.85 to 582.64) | 13182.72(12838.02 to 13604.16) |  | 31.47(23.77 to 38.10) | 7.74(5.26 to 10.53) | 23.73(17.24 to 29.96) |
| **1996** | 13753.44(13410.37 to 14175.99) | 547.65(515.20 to 580.61) | 13205.78(12862.31 to 13628.40) |  | 31.31(23.62 to 37.84) | 7.61(5.17 to 10.39) | 23.70(17.16 to 29.83) |
| **1997** | 13777.04(13435.49 to 14200.31) | 546.70(514.08 to 579.59) | 13230.34(12887.37 to 13653.79) |  | 31.43(23.55 to 38.01) | 7.54(5.17 to 10.28) | 23.89(17.28 to 30.17) |
| **1998** | 13802.49(13462.44 to 14228.86) | 546.27(513.40 to 578.88) | 13256.21(12911.83 to 13679.91) |  | 31.47(23.58 to 37.84) | 7.52(5.19 to 10.08) | 23.95(17.38 to 30.04) |
| **1999** | 13830.25(13490.89 to 14257.84) | 545.98(513.21 to 578.45) | 13284.27(12940.00 to 13709.31) |  | 31.53(23.60 to 37.62) | 7.57(5.25 to 10.00) | 23.96(17.06 to 29.87) |
| **2000** | 13860.67(13520.41 to 14289.38) | 545.99(513.50 to 578.37) | 13314.68(12966.60 to 13741.44) |  | 31.48(23.69 to 37.64) | 7.58(5.24 to 9.83) | 23.90(17.19 to 29.36) |
| **2001** | 13893.90(13551.86 to 14323.35) | 545.82(513.51 to 578.29) | 13348.08(13004.61 to 13774.23) |  | 31.26(23.47 to 37.26) | 7.53(5.30 to 9.71) | 23.73(17.07 to 29.25) |
| **2002** | 13930.13(13589.41 to 14356.33) | 545.84(513.61 to 578.02) | 13384.29(13043.06 to 13809.96) |  | 30.97(23.43 to 36.48) | 7.49(5.29 to 9.50) | 23.48(17.10 to 28.59) |
| **2003** | 13969.03(13625.95 to 14393.22) | 546.04(513.87 to 577.96) | 13422.98(13082.69 to 13846.00) |  | 30.67(23.33 to 36.46) | 7.44(5.25 to 9.36) | 23.22(16.86 to 28.39) |
| **2004** | 14010.27(13664.98 to 14436.26) | 546.48(514.37 to 578.40) | 13463.78(13124.28 to 13889.28) |  | 30.56(22.98 to 36.39) | 7.43(5.28 to 9.18) | 23.13(16.83 to 28.15) |
| **2005** | 14053.56(13710.64 to 14482.24) | 547.59(515.54 to 579.62) | 13505.98(13165.03 to 13931.74) |  | 30.84(22.91 to 36.72) | 7.50(5.25 to 9.24) | 23.34(16.80 to 28.41) |
| **2006** | 14093.81(13751.92 to 14519.79) | 550.21(518.33 to 582.00) | 13543.60(13202.09 to 13972.34) |  | 30.34(22.74 to 35.93) | 7.41(5.23 to 9.13) | 22.93(16.83 to 27.69) |
| **2007** | 14130.09(13788.09 to 14555.66) | 554.77(522.98 to 586.37) | 13575.32(13233.64 to 14004.65) |  | 29.93(22.70 to 35.38) | 7.35(5.24 to 9.08) | 22.57(16.81 to 27.34) |
| **2008** | 14167.95(13823.17 to 14591.17) | 559.73(527.88 to 591.17) | 13608.21(13266.47 to 14035.53) |  | 29.66(22.49 to 35.29) | 7.36(5.21 to 9.00) | 22.31(16.87 to 27.13) |
| **2009** | 14209.05(13863.96 to 14629.90) | 563.47(531.48 to 594.48) | 13645.58(13302.47 to 14072.49) |  | 29.10(22.46 to 34.69) | 7.27(5.16 to 8.86) | 21.83(16.57 to 26.46) |
| **2010** | 14252.90(13909.97 to 14672.58) | 564.78(533.29 to 595.46) | 13688.12(13346.43 to 14114.99) |  | 28.83(22.31 to 34.35) | 7.28(5.17 to 8.88) | 21.56(16.67 to 26.27) |
| **2011** | 14300.84(13956.98 to 14725.30) | 563.91(532.16 to 594.56) | 13736.92(13390.50 to 14165.38) |  | 28.59(22.00 to 34.08) | 7.23(5.12 to 8.80) | 21.35(16.59 to 25.84) |
| **2012** | 14352.41(14009.61 to 14778.57) | 562.77(530.98 to 593.44) | 13789.64(13439.11 to 14220.18) |  | 28.17(21.89 to 33.79) | 7.15(5.02 to 8.73) | 21.02(16.43 to 25.49) |
| **2013** | 14404.50(14061.94 to 14832.75) | 561.37(529.97 to 591.79) | 13843.13(13487.71 to 14276.21) |  | 28.41(21.70 to 33.89) | 7.19(4.97 to 8.74) | 21.22(16.30 to 25.87) |
| **2014** | 14454.91(14105.97 to 14886.10) | 559.67(528.38 to 590.13) | 13895.25(13538.65 to 14331.40) |  | 28.47(21.75 to 33.99) | 7.19(4.95 to 8.70) | 21.28(16.57 to 26.00) |
| **2015** | 14502.44(14148.02 to 14936.53) | 557.94(526.69 to 588.37) | 13944.50(13587.06 to 14383.88) |  | 28.71(21.54 to 34.29) | 7.22(4.91 to 8.69) | 21.49(16.51 to 26.17) |
| **2016** | 14547.17(14197.64 to 14989.84) | 555.38(523.68 to 585.61) | 13991.79(13631.40 to 14434.66) |  | 28.62(21.49 to 34.12) | 7.17(4.89 to 8.63) | 21.45(16.31 to 25.98) |
| **2017** | 14587.13(14229.88 to 15029.19) | 552.58(520.29 to 583.36) | 14034.55(13673.40 to 14478.52) |  | 28.50(21.16 to 34.31) | 7.07(4.79 to 8.48) | 21.42(16.21 to 26.03) |
| **2018** | 14630.78(14271.18 to 15078.16) | 550.35(518.13 to 581.38) | 14080.44(13717.29 to 14529.43) |  | 28.43(21.20 to 34.29) | 7.01(4.73 to 8.44) | 21.42(16.24 to 25.97) |
| **2019** | 14684.30(14321.55 to 15134.03) | 548.42(515.82 to 580.15) | 14135.87(13766.18 to 14585.22) |  | 28.39(21.35 to 34.10) | 6.96(4.75 to 8.35) | 21.43(16.18 to 26.20) |
