# Supplementary material for: Global Burden of Bacterial Skin Diseases: A Systematic Analysis Combined With Sociodemographic Index, 1990–2019
Source: Front Med (Lausanne). 2022 Apr 25;9:861115. doi: 10.3389/fmed.2022.861115 (PMC9084187; doi:10.3389/fmed.2022.861115)
Supplement: Supplementary file 2 [file Table_2.docx]

S2 Table Age-standardized Incidence and DALYs and of bacterial skin diseases (by sexes), 1990-2019.

| **Year** | **Bacterial skin diseases** | | | | |
| --- | --- | --- | --- | --- | --- |
|  | **Incidence** | |  | **DALYs (Disability-Adjusted Life Years)** | |
|  | **Male** | **Female** |  | **Male** | **Female** |
| **1990** | 15091.62(14713.71 to 15561.12) | 12278.14(11975.31 to 12648.59) |  | 33.02(20.52 to 42.24) | 30.71(20.75 to 39.95) |
| **1991** | 15090.56(14715.43 to 15560.43) | 12290.58(11990.95 to 12661.33) |  | 33.17(20.42 to 42.31) | 30.83(20.94 to 39.95) |
| **1992** | 15091.82(14713.96 to 15559.06) | 12305.59(12005.24 to 12676.27) |  | 32.95(20.53 to 41.69) | 30.93(21.22 to 39.84) |
| **1993** | 15095.38(14715.78 to 15563.34) | 12323.19(12022.78 to 12695.53) |  | 32.92(20.71 to 41.61) | 31.05(21.29 to 39.65) |
| **1994** | 15102.27(14727.74 to 15570.42) | 12344.39(12041.18 to 12718.66) |  | 32.78(20.74 to 41.56) | 31.06(21.36 to 39.49) |
| **1995** | 15113.59(14743.20 to 15584.19) | 12369.48(12067.50 to 12745.81) |  | 32.41(20.66 to 40.81) | 30.95(21.08 to 39.33) |
| **1996** | 15128.33(14754.42 to 15598.07) | 12398.48(12093.49 to 12774.39) |  | 32.13(20.28 to 40.90) | 30.92(21.19 to 39.74) |
| **1997** | 15145.24(14774.50 to 15616.31) | 12430.38(12125.64 to 12807.90) |  | 32.14(20.10 to 41.46) | 31.15(21.09 to 39.91) |
| **1998** | 15164.48(14793.46 to 15638.17) | 12463.77(12157.82 to 12839.73) |  | 32.05(20.00 to 41.20) | 31.31(21.20 to 39.91) |
| **1999** | 15187.14(14813.48 to 15662.32) | 12498.57(12189.15 to 12874.04) |  | 32.01(20.13 to 40.86) | 31.44(21.27 to 39.35) |
| **2000** | 15214.30(14838.73 to 15690.69) | 12534.09(12228.10 to 12910.17) |  | 31.87(20.16 to 40.47) | 31.45(21.57 to 39.05) |
| **2001** | 15246.02(14869.59 to 15720.69) | 12570.41(12265.09 to 12947.44) |  | 31.64(20.03 to 40.28) | 31.22(21.53 to 38.49) |
| **2002** | 15281.78(14904.36 to 15750.14) | 12608.47(12300.73 to 12986.52) |  | 31.34(20.00 to 39.29) | 30.93(21.59 to 37.84) |
| **2003** | 15321.24(14939.64 to 15786.68) | 12648.13(12337.19 to 13024.70) |  | 31.02(20.06 to 38.88) | 30.64(21.65 to 37.31) |
| **2004** | 15363.32(14977.42 to 15830.58) | 12689.59(12377.79 to 13067.68) |  | 30.79(19.81 to 38.64) | 30.64(21.77 to 37.04) |
| **2005** | 15407.52(15025.67 to 15881.56) | 12733.06(12419.04 to 13112.97) |  | 31.08(19.78 to 39.02) | 30.91(21.71 to 37.44) |
| **2006** | 15448.65(15065.35 to 15921.12) | 12773.06(12459.96 to 13152.41) |  | 30.59(19.85 to 38.40) | 30.36(22.11 to 36.78) |
| **2007** | 15485.44(15105.44 to 15957.18) | 12809.16(12499.57 to 13187.10) |  | 30.19(19.58 to 37.68) | 29.91(22.09 to 36.42) |
| **2008** | 15524.21(15145.49 to 16003.25) | 12846.43(12537.24 to 13227.21) |  | 30.00(19.54 to 37.76) | 29.56(22.12 to 35.58) |
| **2009** | 15566.20(15190.19 to 16045.35) | 12887.08(12577.67 to 13266.53) |  | 29.42(19.36 to 37.07) | 29.00(22.21 to 35.10) |
| **2010** | 15609.96(15230.97 to 16083.09) | 12931.44(12617.81 to 13313.33) |  | 29.08(19.22 to 36.79) | 28.80(21.93 to 34.53) |
| **2011** | 15656.48(15277.24 to 16133.45) | 12980.82(12669.26 to 13361.61) |  | 28.83(19.23 to 36.63) | 28.54(21.87 to 34.28) |
| **2012** | 15706.47(15326.41 to 16186.83) | 13033.90(12723.86 to 13421.70) |  | 28.42(19.12 to 36.03) | 28.10(21.88 to 34.08) |
| **2013** | 15757.59(15372.47 to 16235.41) | 13086.89(12775.34 to 13478.83) |  | 28.65(19.12 to 36.09) | 28.35(21.80 to 33.87) |
| **2014** | 15807.82(15418.82 to 16281.73) | 13137.28(12825.17 to 13536.81) |  | 28.73(19.03 to 36.44) | 28.39(22.02 to 34.31) |
| **2015** | 15856.58(15471.14 to 16333.62) | 13183.21(12868.72 to 13586.71) |  | 28.99(19.14 to 36.99) | 28.60(22.02 to 34.17) |
| **2016** | 15903.88(15506.92 to 16386.29) | 13225.00(12906.24 to 13625.40) |  | 29.01(19.03 to 36.75) | 28.41(21.71 to 33.75) |
| **2017** | 15946.12(15551.18 to 16445.57) | 13262.37(12939.44 to 13660.34) |  | 28.88(18.67 to 36.96) | 28.30(21.87 to 33.93) |
| **2018** | 15992.05(15591.47 to 16483.21) | 13303.52(12977.87 to 13705.97) |  | 28.86(18.95 to 36.67) | 28.18(21.84 to 33.64) |
| **2019** | 16048.41(15644.72 to 16545.08) | 13354.01(13024.44 to 13758.49) |  | 28.83(18.83 to 36.69) | 28.12(22.05 to 33.91) |
