# Supplementary material for: Global Burden of Bacterial Skin Diseases: A Systematic Analysis Combined With Sociodemographic Index, 1990–2019
Source: Front Med (Lausanne). 2022 Apr 25;9:861115. doi: 10.3389/fmed.2022.861115 (PMC9084187; doi:10.3389/fmed.2022.861115)
Supplement: Supplementary file 3 [file Table_3.docx]

S3 Table Age-standardized change in(incidence and DALYs) major bacterial skin diseases(by sexes), 1990-2019.

| **Group** | **Incidence(1990-2019)** | | | | | | | |  | **DALYs (Disability-Adjusted Life Years, 1990-2019)** | | | | | | | |
| --- | --- | --- | --- | --- | --- | --- | --- | --- | --- | --- | --- | --- | --- | --- | --- | --- | --- |
|  | **Bacterial skin diseases** | |  | **Cellulitis** | |  | **Pyoderma** | |  | **Bacterial skin diseases** | |  | **Cellulitis** | |  | **Pyoderma** | |
|  | **Male** | **Female** |  | **Male** | **Female** |  | **Male** | **Female** |  | **Male** | **Female** |  | **Male** | **Female** |  | **Male** | **Female** |
| **Global** | 6.34(5.94 to 6.73) | 8.76(8.36 to 9.16) |  | -5.70(-6.32 to -5.10) | -6.06(-6.75 to -5.32) |  | 6.86(6.44 to 7.26) | 9.45(9.03 to 9.85) |  | -12.70(-31.49 to 6.44) | -8.45(-28.18 to 10.44) |  | -10.83(-40.95 to 14.50) | -10.42(-40.40 to 20.21) |  | -13.31(-35.05 to 12.00) | -7.81(-30.90 to 16.20) |
| **High SDI** | -5.25(-5.88 to -4.61) | -2.92(-3.55 to -2.24) |  | 3.27(2.12 to 4.44) | 9.42(8.12 to 10.68) |  | -6.12(-6.83 to -5.42) | -4.29(-4.97 to -3.57) |  | 52.76(-6.39 to 90.70) | 68.10(28.31 to 95.51) |  | 45.10(-17.35 to 79.92) | 64.07(22.11 to 87.01) |  | 62.21(-1.55 to 127.28) | 72.85(28.91 to 117.89) |
| **High-middle SDI** | -1.52(-2.07 to -0.94) | 0.81(0.24 to 1.40) |  | -10.98(-11.77 to -10.17) | -10.04(-10.97 to -9.06) |  | -1.14(-1.71 to -0.55) | 1.27(0.68 to 1.86) |  | -10.59(-31.11 to 5.09) | -7.48(-25.63 to 7.26) |  | -11.25(-39.97 to 10.25) | -6.82(-29.29 to 18.70) |  | -10.20(-34.80 to 10.87) | -7.85(-27.08 to 11.04) |
| **Middle SDI** | 6.90(6.43 to 7.39) | 13.70(13.08 to 14.27) |  | 6.59(5.83 to 7.32) | 10.11(9.32 to 10.89) |  | 6.91(6.42 to 7.42) | 13.80(13.18 to 14.39) |  | -24.81(-36.32 to -5.54) | -21.10(-31.08 to -3.99) |  | -29.49(-52.14 to 3.52) | -23.21(-39.69 to 1.89) |  | -23.46(-37.79 to 1.44) | -20.50(-31.49 to -2.64) |
| **Low-middle SDI** | 4.01(3.11 to 4.90) | 6.37(5.60 to 7.20) |  | 3.16(2.35 to 3.99) | 7.16(6.25 to 8.05) |  | 4.03(3.10 to 4.94) | 6.35(5.56 to 7.20) |  | -29.04(-44.48 to -2.98) | -25.05(-43.46 to -1.22) |  | -33.12(-50.24 to -4.90) | -36.85(-62.18 to -2.64) |  | -28.39(-45.22 to 1.44) | -22.59(-43.19 to 3.02) |
| **Low SDI** | -5.80(-6.53 to -5.03) | 3.51(2.78 to 4.22) |  | -6.11(-6.94 to -5.26) | 3.59(2.66 to 4.49) |  | -5.80(-6.53 to -5.01) | 3.51(2.76 to 4.24) |  | -15.50(-33.20 to 6.96) | -18.49(-38.32 to 4.01) |  | -23.77(-47.64 to 8.67) | -31.01(-62.89 to 10.71) |  | -13.56(-34.51 to 14.02) | -15.78(-38.11 to 7.65) |
