# Supplementary material for: Global Burden of Bacterial Skin Diseases: A Systematic Analysis Combined With Sociodemographic Index, 1990–2019
Source: Front Med (Lausanne). 2022 Apr 25;9:861115. doi: 10.3389/fmed.2022.861115 (PMC9084187; doi:10.3389/fmed.2022.861115)
Supplement: Supplementary file 4 [file Table_4.docx]

S4 Table Age-standardized incidence and DALYs of bacterial skin diseases (by different SDI, both sexes), 1990-2019.

| **Bacterial skin diseases** | | | | | | | | | | | |
| --- | --- | --- | --- | --- | --- | --- | --- | --- | --- | --- | --- |
|  | **Incidence** | | | | |  | **DALYs (Disability-Adjusted Life Years)** | | | | |
|  | **High SDI** | **High-middle SDI** | **Middle SDI** | **Low-middle SDI** | **Low SDI** |  | **High SDI** | **High-middle SDI** | **Middle SDI** | **Low-middle SDI** | **Low SDI** |
| **1990** | 15923.39(15504.73to16362.60) | 11042.79(10753.02to11401.10) | 10910.29(10636.01to11261.22) | 17660.37(17212.94to18228.32) | 17247.87(16784.87to17805.50) |  | 12.55(9.08to18.47) | 20.21(15.67to26.28) | 29.98(20.89to34.65) | 58.06(33.51to72.40) | 49.38(40.06to61.17) |
| **1991** | 15810.98(15407.17to16253.86) | 11047.68(10758.05to11402.38) | 10325.35(10061.96to10653.76) | 17518.05(17062.21to18065.49) | 16201.20(15763.18to16733.08) |  | 12.65(9.18to18.58) | 20.45(15.84to26.43) | 30.01(20.96to34.35) | 57.98(33.17to71.45) | 49.21(39.95to60.52) |
| **1992** | 15709.75(15315.95to16150.42) | 11055.54(10763.67to11407.37) | 10478.74(10214.58to10815.41) | 17537.87(17083.50to18084.21) | 16197.18(15758.59to16726.99) |  | 12.78(9.33to18.71) | 20.50(16.17to26.57) | 29.58(20.85to33.85) | 57.69(32.93to70.60) | 49.09(39.68to59.88) |
| **1993** | 15620.52(15232.34to16055.84) | 11059.37(10772.77to11409.08) | 10430.18(10165.77to10762.59) | 17556.14(17101.57to18112.53) | 16198.36(15758.81to16723.36) |  | 12.96(9.49to19.05) | 20.85(16.94to26.92) | 29.16(20.73to33.26) | 57.46(32.76to69.69) | 49.00(39.60to59.55) |
| **1994** | 15545.75(15154.46to15981.91) | 11060.19(10779.63to11410.07) | 10584.51(10313.38to10922.78) | 17575.50(17120.10to18136.54) | 16198.59(15767.97to16732.37) |  | 13.16(9.69to19.15) | 20.97(16.99to26.84) | 28.73(20.58to32.80) | 56.94(32.50to68.97) | 48.76(39.66to58.62) |
| **1995** | 15490.94(15105.51to15926.13) | 11060.27(10779.30to11409.08) | 10638.49(10361.83to10979.57) | 17593.19(17133.00to18162.91) | 16198.35(15765.94to16740.52) |  | 13.37(9.86to19.38) | 20.59(16.57to26.50) | 28.17(20.64to32.16) | 56.36(32.39to67.36) | 48.36(39.07to57.75) |
| **1996** | 15453.15(15065.77to15890.23) | 11055.96(10777.71to11401.03) | 10589.62(10316.71to10928.22) | 17611.73(17155.75to18175.70) | 16202.24(15770.80to16735.94) |  | 13.59(10.08to19.59) | 20.15(15.95to25.96) | 27.77(20.36to31.81) | 56.21(31.86to67.90) | 48.02(38.43to56.59) |
| **1997** | 15425.92(15037.91to15852.38) | 11047.74(10775.55to11391.27) | 10642.90(10370.61to10980.63) | 17635.69(17179.86to18194.91) | 16214.02(15778.31to16748.62) |  | 13.88(10.35to19.88) | 19.77(15.79to25.50) | 27.60(20.28to31.77) | 56.97(32.68to68.63) | 48.05(38.54to56.57) |
| **1998** | 15404.18(15013.94to15819.37) | 11036.93(10766.80to11379.63) | 10695.90(10423.17to11035.43) | 17662.34(17201.40to18219.68) | 16233.01(15798.51to16755.31) |  | 14.32(10.75to20.45) | 19.59(15.54to25.49) | 27.34(20.20to31.47) | 56.96(31.78to68.20) | 48.00(38.51to56.15) |
| **1999** | 15384.63(15001.28to15799.75) | 11026.77(10754.47to11368.37) | 10749.59(10476.69to11095.52) | 17691.69(17233.15to18242.50) | 16254.84(15823.26to16779.59) |  | 14.93(11.08to20.96) | 19.64(15.67to25.40) | 27.03(20.02to31.16) | 56.55(31.42to66.41) | 47.79(38.37to55.63) |
| **2000** | 15365.41(14983.91to15783.12) | 11022.44(10751.80to11361.52) | 10806.01(10532.97to11154.94) | 17720.04(17264.03to18269.30) | 16274.75(15848.25to16799.57) |  | 15.53(11.40to21.61) | 19.63(15.73to25.45) | 26.57(20.09to30.78) | 56.07(31.61to66.12) | 47.63(38.06to55.16) |
| **2001** | 15340.85(14957.22to15755.83) | 11023.51(10755.15to11362.53) | 10862.65(10588.80to11215.07) | 17753.00(17300.90to18300.05) | 16295.67(15861.67to16824.83) |  | 16.12(11.77to22.28) | 19.35(15.60to25.24) | 25.91(19.73to30.11) | 55.46(31.48to65.15) | 47.28(37.72to54.99) |
| **2002** | 15315.44(14935.99to15723.60) | 11029.32(10759.18to11368.61) | 11021.53(10742.51to11378.50) | 17794.41(17341.68to18341.51) | 16321.81(15887.68to16854.73) |  | 16.65(12.04to22.83) | 18.91(15.32to24.82) | 25.26(19.56to29.32) | 54.67(30.90to63.68) | 47.09(37.43to54.58) |
| **2003** | 15292.19(14917.17to15700.01) | 11036.12(10764.32to11379.49) | 11078.98(10798.31to11436.90) | 17838.98(17385.53to18392.11) | 16352.73(15922.32to16883.68) |  | 17.11(12.38to23.36) | 18.60(14.95to24.49) | 24.69(19.44to28.72) | 53.58(30.79to62.61) | 46.91(37.71to54.54) |
| **2004** | 15270.56(14894.02to15678.69) | 11041.94(10768.83to11385.28) | 11137.09(10854.12to11495.58) | 17884.89(17431.27to18445.01) | 16385.62(15956.28to16928.70) |  | 17.49(12.52to23.81) | 18.42(14.72to24.37) | 24.26(19.22to28.13) | 52.94(30.54to61.45) | 46.67(37.68to54.01) |
| **2005** | 15254.62(14870.32to15664.20) | 11045.98(10774.45to11390.46) | 11094.93(10811.98to11449.85) | 17930.03(17476.24to18486.99) | 16417.16(15979.17to16965.04) |  | 17.84(12.70to24.27) | 18.47(14.67to24.29) | 24.12(19.17to28.17) | 53.64(30.94to62.11) | 46.80(37.21to54.48) |
| **2006** | 15250.53(14878.85to15650.13) | 11032.78(10760.57to11375.02) | 11146.44(10864.07to11500.40) | 17975.26(17523.41to18524.16) | 16453.50(16020.58to17003.80) |  | 18.17(12.84to24.58) | 18.10(14.22to23.89) | 23.75(19.05to27.79) | 51.94(31.05to60.27) | 46.20(37.02to53.25) |
| **2007** | 15261.52(14897.91to15656.67) | 11000.80(10728.47to11341.61) | 11290.04(11008.85to11642.92) | 18024.50(17575.51to18573.86) | 16496.70(16063.10to17032.85) |  | 18.50(12.99to25.02) | 17.86(14.02to23.63) | 23.40(18.73to27.45) | 50.52(30.93to58.45) | 45.73(37.12to52.52) |
| **2008** | 15277.93(14914.39to15681.40) | 10962.55(10691.81to11302.99) | 11333.24(11055.15to11681.67) | 18075.17(17627.70to18636.86) | 16692.59(16248.70to17236.74) |  | 18.85(13.15to25.47) | 17.81(13.86to23.39) | 23.28(18.75to27.39) | 49.27(30.79to57.18) | 45.25(36.70to52.06) |
| **2009** | 15287.24(14926.93to15695.31) | 10929.43(10659.06to11268.68) | 11395.32(11117.69to11747.02) | 18124.22(17677.65to18678.49) | 16736.23(16293.13to17287.68) |  | 19.12(13.33to25.80) | 17.62(13.70to23.10) | 22.89(18.43to27.12) | 47.04(30.55to54.32) | 44.36(36.17to50.96) |
| **2010** | 15283.79(14921.31to15694.81) | 10914.04(10645.14to11250.91) | 11441.56(11165.45to11793.19) | 18169.23(17728.30to18714.28) | 16770.90(16332.07to17320.79) |  | 19.39(13.29to26.10) | 17.77(13.69to23.21) | 22.64(18.50to26.83) | 45.73(30.34to53.07) | 43.65(35.59to50.35) |
| **2011** | 15268.99(14905.08to15675.73) | 10914.77(10645.04to11249.26) | 11516.33(11238.83to11868.67) | 18213.24(17749.79to18763.72) | 16799.75(16356.53to17351.91) |  | 19.63(13.31to26.40) | 17.83(13.54to23.23) | 22.48(18.26to26.75) | 44.58(30.05to51.66) | 43.00(35.02to49.69) |
| **2012** | 15259.37(14894.84to15668.44) | 10922.86(10655.10to11249.64) | 11569.88(11290.45to11921.88) | 18259.79(17794.39to18824.32) | 16829.73(16388.65to17386.47) |  | 19.81(13.20to26.68) | 17.93(13.49to23.35) | 22.45(18.28to26.77) | 42.56(29.92to49.31) | 41.95(34.32to48.44) |
| **2013** | 15254.36(14895.52to15662.49) | 10933.64(10663.12to11261.27) | 11632.84(11351.83to11986.39) | 18305.31(17846.37to18876.06) | 16858.79(16422.09to17417.87) |  | 19.97(13.13to26.87) | 18.21(13.37to23.52) | 22.76(18.23to27.17) | 42.82(29.75to49.91) | 41.93(34.19to48.94) |
| **2014** | 15252.40(14896.00to15657.31) | 10944.30(10677.56to11275.93) | 11692.31(11409.90to12051.27) | 18346.72(17877.24to18905.45) | 16886.05(16447.46to17448.26) |  | 20.13(13.20to27.07) | 18.44(13.34to23.61) | 23.01(18.25to27.43) | 42.37(29.28to49.34) | 41.74(33.82to48.46) |
| **2015** | 15253.70(14896.47to15662.20) | 10958.49(10690.25to11297.53) | 11747.96(11464.56to12109.06) | 18382.03(17907.89to18948.18) | 16908.59(16465.68to17474.57) |  | 20.30(13.27to27.22) | 18.74(13.27to23.83) | 23.24(18.31to27.69) | 42.75(29.64to49.87) | 41.79(33.83to48.62) |
| **2016** | 15259.02(14902.49to15667.09) | 10975.97(10704.81to11314.45) | 11797.22(11503.73to12163.09) | 18415.85(17947.69to18986.91) | 16935.69(16497.43to17509.64) |  | 20.36(13.26to27.37) | 18.87(13.28to23.99) | 23.14(18.04to27.47) | 42.50(29.04to49.80) | 41.60(33.72to48.45) |
| **2017** | 15267.00(14903.83to15670.84) | 10988.49(10712.37to11336.12) | 11851.74(11557.66to12226.85) | 18442.18(17988.83to19025.36) | 16956.31(16503.21to17521.38) |  | 20.22(13.15to27.18) | 18.61(13.00to23.85) | 23.14(18.02to27.44) | 42.45(28.90to49.94) | 41.45(33.63to48.62) |
| **2018** | 15273.11(14905.91to15680.72) | 10997.59(10722.70to11340.70) | 11904.30(11604.18to12274.30) | 18463.48(18004.98to19039.39) | 16964.57(16519.89to17533.43) |  | 20.14(13.16to27.15) | 18.51(13.03to23.97) | 23.09(18.04to27.43) | 42.26(29.41to49.98) | 41.29(33.49to48.27) |
| **2019** | 15276.52(14896.68to15673.53) | 11010.68(10736.91to11356.88) | 11974.15(11676.49to12338.95) | 18489.09(18017.94to19059.53) | 16972.32(16528.17to17544.18) |  | 20.09(13.22to27.02) | 18.48(12.90to23.84) | 23.06(17.93to27.64) | 42.16(28.74to50.30) | 40.95(32.97to47.96) |
