# Supplementary material for: Global Burden of Bacterial Skin Diseases: A Systematic Analysis Combined With Sociodemographic Index, 1990–2019
Source: Front Med (Lausanne). 2022 Apr 25;9:861115. doi: 10.3389/fmed.2022.861115 (PMC9084187; doi:10.3389/fmed.2022.861115)
Supplement: Supplementary file 5 [file Table_5.docx]

S5 Table Incidence of major bacterial skin diseases in 2019, and change of Age-standardized incidence during the periods 1990-2019 for both sexes in 204 countries.

| **Incidence** | | | | | | | | |
| --- | --- | --- | --- | --- | --- | --- | --- | --- |
|  | **Bacterial skin diseases** | |  | **Cellulitis** | |  | **Pyoderma** | |
|  | **Number of incident cases, 2019** | **Annualised rate of change of age-standardized incidence(1990-2019)(%)** |  | **Number of incident cases, 2019** | **Annualised rate of change of age-standardized incidence(1990-2019)(%)** |  | **Number of incident cases, 2019** | **Annualised rate of change of age-standardized incidence(1990-2019)(%)** |
| **Afghanistan** | 12337.84(11922.23to12835.91) | 1.26(-0.84to3.54) |  | 315.96(294.16to336.90) | 0.85(-1.99to3.56) |  | 12021.88(11608.43to12523.08) | 1.27(-0.92to3.62) |
| **Albania** | 16131.18(15642.34to16693.76) | 0.88(-1.10to3.09) |  | 453.95(421.47to484.66) | 1.93(-0.82to5.02) |  | 15677.23(15193.46to16233.09) | 0.85(-1.19to3.11) |
| **Algeria** | 12351.84(11907.47to12850.40) | 1.12(-0.81to3.31) |  | 316.53(294.49to338.67) | 0.40(-2.27to3.07) |  | 12035.30(11593.87to12539.64) | 1.14(-0.86to3.37) |
| **American Samoa** | 6714.55(6475.75to6971.39) | 4.45(2.33to6.76) |  | 209.19(193.76to223.93) | 3.36(0.75to6.06) |  | 6505.36(6264.16to6763.66) | 4.48(2.32to6.91) |
| **Andorra** | 23678.05(22870.65to24538.34) | 0.26(-2.03to2.45) |  | 531.08(494.30to566.33) | 2.27(-0.66to5.59) |  | 23146.98(22346.05to23998.85) | 0.22(-2.11to2.44) |
| **Angola** | 18925.96(18229.57to19740.76) | 2.34(0.26to4.42) |  | 391.33(364.07to418.19) | 1.96(-0.66to4.69) |  | 18534.63(17821.11to19340.28) | 2.35(0.21to4.46) |
| **Antigua and Barbuda** | 18843.04(18196.07to19521.07) | 1.84(-0.42to4.22) |  | 546.02(504.55to586.06) | 4.10(0.90to7.32) |  | 18297.02(17648.20to18990.11) | 1.78(-0.57to4.23) |
| **Argentina** | 10576.54(10227.66to10964.65) | 1.76(-0.33to3.82) |  | 526.48(490.16to562.52) | 7.46(4.46to10.65) |  | 10050.06(9710.11to10439.19) | 1.48(-0.71to3.65) |
| **Armenia** | 9046.20(8729.67to9428.45) | 1.58(-0.79to4.04) |  | 406.87(378.33to437.57) | 3.30(0.63to6.02) |  | 8639.33(8325.52to9021.15) | 1.50(-1.03to4.05) |
| **Australia** | 24964.52(24183.89to25818.02) | 1.27(-0.69to3.47) |  | 1926.39(1811.22to2038.34) | 6.76(4.18to9.76) |  | 23038.13(22257.84to23881.56) | 0.84(-1.26to3.21) |
| **Austria** | 32131.98(31091.01to33195.96) | 1.80(-0.40to3.77) |  | 845.25(792.64to897.70) | -4.70(-7.19to-1.98) |  | 31286.72(30241.13to32345.75) | 1.99(-0.29to4.00) |
| **Azerbaijan** | 9073.15(8765.34to9424.81) | 2.11(-0.15to4.36) |  | 408.23(379.89to438.14) | 3.79(0.97to6.65) |  | 8664.92(8353.19to9016.65) | 2.03(-0.32to4.41) |
| **Bahamas** | 18791.47(18118.47to19515.49) | 1.37(-0.84to3.51) |  | 542.59(501.42to583.88) | 3.15(0.04to6.54) |  | 18248.88(17584.67to18984.42) | 1.32(-0.94to3.50) |
| **Bahrain** | 12485.03(12034.00to13016.52) | 1.20(-0.96to3.39) |  | 332.34(308.95to355.36) | -0.64(-3.30to2.21) |  | 12152.69(11699.83to12683.31) | 1.26(-0.97to3.52) |
| **Bangladesh** | 22487.65(21777.34to23289.47) | 5.16(2.92to7.59) |  | 380.27(354.90to406.46) | 4.47(1.48to7.59) |  | 22107.37(21391.82to22914.36) | 5.18(2.89to7.66) |
| **Barbados** | 19049.13(18389.32to19770.23) | 2.43(0.23to4.63) |  | 582.60(541.69to625.34) | 6.77(3.48to9.82) |  | 18466.53(17805.54to19197.09) | 2.30(0.01to4.57) |
| **Belarus** | 11919.76(11490.90to12397.06) | 1.34(-0.83to3.54) |  | 1008.17(940.22to1073.56) | 2.37(-0.36to5.25) |  | 10911.59(10501.78to11390.36) | 1.25(-1.07to3.62) |
| **Belgium** | 20195.08(19512.80to20939.42) | 1.05(-1.38to3.64) |  | 677.19(634.10to724.81) | 2.30(-0.53to5.47) |  | 19517.89(18852.13to20249.59) | 1.01(-1.56to3.71) |
| **Belize** | 18841.34(18170.18to19573.20) | 1.01(-1.21to3.30) |  | 543.36(500.29to585.55) | 2.71(-0.62to6.03) |  | 18297.98(17635.37to19033.73) | 0.96(-1.33to3.35) |
| **Benin** | 17512.30(16824.71to18225.23) | 2.76(0.64to5.08) |  | 390.85(363.68to419.33) | 1.57(-1.09to4.36) |  | 17121.45(16432.82to17836.57) | 2.79(0.59to5.18) |
| **Bermuda** | 18818.87(18179.42to19539.92) | 1.32(-0.89to3.56) |  | 543.81(502.21to583.70) | 3.33(0.12to6.53) |  | 18275.05(17626.36to19002.34) | 1.26(-1.04to3.54) |
| **Bhutan** | 22682.60(21972.74to23543.91) | 6.44(4.12to8.87) |  | 379.86(354.18to405.86) | 4.51(1.86to7.50) |  | 22302.73(21592.96to23155.89) | 6.47(4.10to8.95) |
| **Bolivia (Plurinational State of)** | 18926.04(18336.27to19608.79) | 1.33(-0.71to3.54) |  | 524.21(483.19to565.12) | 5.98(2.83to9.27) |  | 18401.83(17806.45to19084.09) | 1.20(-0.83to3.50) |
| **Bosnia and Herzegovina** | 16087.90(15584.81to16643.56) | 1.03(-1.04to3.17) |  | 453.03(420.21to483.06) | 2.22(-0.61to5.11) |  | 15634.87(15128.97to16198.01) | 0.99(-1.11to3.20) |
| **Botswana** | 29572.85(28562.63to30659.43) | 2.03(-0.06to4.35) |  | 683.55(638.61to726.63) | 2.48(0.02to5.24) |  | 28889.30(27864.92to29993.50) | 2.02(-0.10to4.43) |
| **Brazil** | 19863.44(19331.18to20476.12) | 1.72(1.06to2.38) |  | 652.21(605.48to698.83) | 0.31(-0.90to1.72) |  | 19211.23(18682.81to19826.32) | 1.77(1.09to2.44) |
| **Brunei Darussalam** | 18441.57(17723.39to19181.34) | -0.14(-2.64to2.44) |  | 1058.54(980.75to1133.87) | 1.20(-1.88to4.54) |  | 17383.04(16662.70to18131.02) | -0.22(-2.86to2.58) |
| **Bulgaria** | 16118.60(15620.48to16656.18) | 1.24(-0.77to3.24) |  | 453.31(421.79to484.20) | 2.40(-0.24to5.13) |  | 15665.29(15175.13to16219.10) | 1.20(-0.86to3.26) |
| **Burkina Faso** | 17508.11(16877.21to18260.44) | 2.71(0.44to5.07) |  | 390.85(361.77to418.85) | 1.53(-1.25to4.37) |  | 17117.26(16489.87to17889.38) | 2.73(0.42to5.14) |
| **Burundi** | 12514.51(12093.95to12960.90) | 2.10(0.14to4.32) |  | 255.00(236.74to272.84) | 1.74(-1.13to4.60) |  | 12259.50(11851.72to12698.72) | 2.11(0.10to4.38) |
| **Cabo Verde** | 17553.94(16910.25to18306.99) | 3.09(0.74to5.46) |  | 392.77(365.02to421.22) | 2.10(-0.56to4.99) |  | 17161.17(16504.61to17912.97) | 3.12(0.72to5.55) |
| **Cambodia** | 5575.02(5375.06to5791.01) | 5.58(3.38to7.91) |  | 324.89(302.34to346.55) | 6.71(3.88to9.71) |  | 5250.13(5052.37to5465.26) | 5.51(3.19to8.04) |
| **Cameroon** | 17582.06(16963.47to18309.40) | 2.53(0.09to4.90) |  | 391.01(363.75to419.09) | 1.55(-1.15to4.21) |  | 17191.05(16565.84to17921.36) | 2.55(0.07to4.98) |
| **Canada** | 4178.38(4011.22to4334.95) | 1.68(-0.05to3.51) |  | 2653.72(2501.52to2812.25) | 2.49(-0.13to5.10) |  | 1524.66(1478.90to1577.08) | 0.30(-1.33to2.12) |
| **Central African Republic** | 18879.77(18167.58to19719.99) | 2.35(-0.02to4.76) |  | 388.66(361.96to416.37) | 1.65(-1.11to4.41) |  | 18491.11(17767.23to19344.57) | 2.36(-0.04to4.83) |
| **Chad** | 17469.55(16837.96to18203.13) | 2.54(0.37to5.00) |  | 391.29(364.10to418.54) | 1.55(-1.60to4.21) |  | 17078.25(16444.25to17829.30) | 2.56(0.32to5.09) |
| **Chile** | 10535.61(10196.93to10932.21) | 1.22(-0.79to3.37) |  | 534.70(498.45to569.93) | 6.06(3.33to8.83) |  | 10000.91(9658.00to10396.64) | 0.97(-1.11to3.26) |
| **China** | 6248.79(6073.21to6453.62) | -4.48(-5.08to-3.83) |  | 84.00(77.42to90.31) | -22.97(-24.18to-21.84) |  | 6164.80(5987.81to6364.95) | -4.16(-4.78to-3.52) |
| **Colombia** | 19569.52(18942.25to20228.54) | 0.70(-1.51to3.04) |  | 710.43(652.51to767.69) | 3.07(-0.58to6.94) |  | 18859.09(18231.31to19511.41) | 0.61(-1.72to3.06) |
| **Comoros** | 12540.86(12116.49to12978.39) | 2.18(0.03to4.22) |  | 256.04(237.85to273.67) | 1.80(-1.08to4.57) |  | 12284.82(11863.84to12721.48) | 2.19(-0.03to4.25) |
| **Congo** | 18998.13(18262.64to19816.86) | 2.82(0.63to5.19) |  | 395.20(367.62to423.38) | 2.80(-0.08to5.45) |  | 18602.93(17870.58to19438.92) | 2.82(0.57to5.27) |
| **Cook Islands** | 6533.11(6294.19to6803.18) | 2.54(0.17to4.96) |  | 209.16(193.94to224.35) | 3.11(0.58to5.79) |  | 6323.94(6089.23to6593.84) | 2.52(0.10to5.06) |
| **Costa Rica** | 19478.74(18853.59to20165.16) | 0.00(-2.29to2.28) |  | 707.18(653.08to765.17) | 2.38(-0.98to6.12) |  | 18771.56(18143.04to19452.97) | -0.09(-2.45to2.28) |
| **Croatia** | 17587.64(17070.33to18177.56) | 2.10(0.14to4.08) |  | 299.56(279.08to318.71) | 3.25(0.55to5.89) |  | 17288.08(16768.41to17877.53) | 2.08(0.11to4.10) |
| **Cuba** | 18921.72(18260.18to19696.33) | 1.60(-0.59to3.91) |  | 550.85(507.35to592.58) | 4.42(1.17to7.51) |  | 18370.87(17707.62to19136.94) | 1.52(-0.75to3.91) |
| **Cyprus** | 23230.99(22443.69to24110.75) | 0.67(-1.47to2.98) |  | 312.21(292.07to333.71) | 3.00(0.66to5.56) |  | 22918.78(22137.97to23797.56) | 0.64(-1.54to2.99) |
| **Czechia** | 18538.11(17940.47to19122.44) | 1.90(-0.03to4.00) |  | 551.61(512.63to588.93) | 4.42(1.84to7.34) |  | 17986.50(17394.58to18568.43) | 1.83(-0.16to4.01) |
| **C么te d'Ivoire** | 17578.17(16969.51to18321.38) | 2.32(-0.02to4.49) |  | 392.22(364.25to419.77) | 1.59(-1.12to4.35) |  | 17185.95(16572.89to17932.45) | 2.34(-0.09to4.55) |
| **Democratic People's Republic of Korea** | 7132.68(6913.98to7377.26) | 4.93(2.62to7.16) |  | 122.71(113.47to131.53) | 3.90(1.03to6.67) |  | 7009.97(6795.10to7256.70) | 4.95(2.59to7.21) |
| **Democratic Republic of the Congo** | 18945.59(18255.09to19728.97) | 2.52(0.30to4.81) |  | 391.77(364.33to419.38) | 1.73(-0.91to4.29) |  | 18553.81(17858.95to19352.08) | 2.54(0.31to4.90) |
| **Denmark** | 29318.42(28380.54to30377.22) | 1.35(-0.68to3.47) |  | 339.18(316.99to361.94) | 4.40(1.45to7.29) |  | 28979.24(28041.06to30025.69) | 1.31(-0.72to3.47) |
| **Djibouti** | 12564.19(12111.57to13029.28) | 2.00(0.10to3.99) |  | 257.24(238.55to274.54) | 1.87(-0.84to4.92) |  | 12306.94(11859.24to12768.75) | 2.00(0.03to4.03) |
| **Dominica** | 19099.93(18437.97to19841.88) | 2.42(0.13to4.57) |  | 566.69(525.68to608.21) | 7.00(3.70to10.50) |  | 18533.24(17862.28to19273.52) | 2.29(-0.07to4.48) |
| **Dominican Republic** | 18876.72(18149.79to19617.93) | 1.89(-0.40to4.22) |  | 544.37(501.87to588.23) | 3.76(0.55to7.10) |  | 18332.34(17621.06to19088.23) | 1.84(-0.49to4.21) |
| **Ecuador** | 19358.85(18769.34to19997.77) | 4.54(1.92to7.17) |  | 546.86(504.37to588.71) | 7.79(4.06to11.17) |  | 18811.99(18223.53to19460.07) | 4.44(1.83to7.14) |
| **Egypt** | 13279.93(12824.37to13783.38) | 0.19(-2.02to2.25) |  | 317.89(295.22to340.26) | 0.84(-2.03to3.62) |  | 12962.04(12515.36to13463.88) | 0.17(-2.08to2.27) |
| **El Salvador** | 19331.22(18707.49to20013.52) | -0.12(-2.51to2.18) |  | 689.05(633.98to743.68) | 0.10(-3.61to3.53) |  | 18642.16(18029.53to19338.41) | -0.13(-2.62to2.26) |
| **Equatorial Guinea** | 19093.92(18377.77to19918.99) | 4.09(1.74to6.35) |  | 396.99(369.38to424.81) | 4.25(1.43to7.34) |  | 18696.93(17971.15to19523.13) | 4.09(1.69to6.40) |
| **Eritrea** | 12530.66(12104.62to13005.37) | 2.30(0.44to4.30) |  | 255.47(237.19to274.37) | 2.11(-0.58to5.06) |  | 12275.19(11854.34to12748.58) | 2.30(0.38to4.32) |
| **Estonia** | 11990.72(11573.99to12457.45) | 1.99(-0.19to4.08) |  | 1017.08(949.03to1084.56) | 3.12(0.12to6.08) |  | 10973.64(10566.80to11436.04) | 1.89(-0.44to4.22) |
| **Eswatini** | 29555.04(28497.59to30634.65) | 2.13(-0.20to4.50) |  | 679.76(634.19to725.49) | 2.21(-0.49to5.04) |  | 28875.28(27841.62to29954.38) | 2.13(-0.28to4.56) |
| **Ethiopia** | 14367.43(13984.49to14799.61) | 3.70(2.68to4.79) |  | 261.54(243.20to279.59) | 1.97(0.56to3.39) |  | 14105.89(13717.50to14540.23) | 3.74(2.69to4.86) |
| **Fiji** | 6579.73(6352.76to6833.62) | 3.70(1.58to5.91) |  | 211.52(195.87to226.95) | 4.17(1.45to6.91) |  | 6368.20(6139.21to6626.74) | 3.68(1.51to5.96) |
| **Finland** | 31145.00(30127.98to32234.76) | 1.10(-1.00to3.40) |  | 415.62(387.04to443.77) | 2.75(-0.15to5.45) |  | 30729.37(29730.08to31800.46) | 1.08(-1.05to3.40) |
| **France** | 23523.83(22670.37to24395.03) | 0.82(-1.48to3.20) |  | 519.55(486.11to556.37) | 2.42(-0.40to5.12) |  | 23004.28(22149.61to23877.71) | 0.79(-1.60to3.22) |
| **Gabon** | 19020.85(18298.68to19860.81) | 2.59(0.40to4.91) |  | 398.98(371.35to425.83) | 3.44(0.82to6.25) |  | 18621.87(17904.78to19466.59) | 2.58(0.36to4.96) |
| **Gambia** | 17502.16(16899.77to18230.30) | 2.27(0.03to4.68) |  | 390.93(363.93to417.26) | 1.17(-1.69to4.23) |  | 17111.23(16503.34to17834.96) | 2.29(-0.01to4.73) |
| **Georgia** | 9371.45(9062.40to9746.73) | 5.64(3.36to8.15) |  | 424.85(397.11to452.27) | 8.13(5.28to10.99) |  | 8946.60(8632.82to9326.46) | 5.53(3.10to8.19) |
| **Germany** | 29531.07(28592.16to30500.92) | 1.56(-0.60to3.78) |  | 749.48(701.14to798.53) | 5.17(2.39to8.07) |  | 28781.59(27843.99to29747.12) | 1.47(-0.74to3.76) |
| **Ghana** | 17539.53(16897.77to18255.04) | 2.67(0.38to5.17) |  | 391.05(363.86to417.38) | 1.38(-1.36to4.39) |  | 17148.48(16510.95to17866.58) | 2.70(0.37to5.24) |
| **Greece** | 23529.56(22704.13to24410.29) | 0.90(-1.40to3.02) |  | 522.68(487.73to557.79) | 2.80(-0.29to5.92) |  | 23006.88(22177.84to23897.92) | 0.86(-1.48to3.02) |
| **Greenland** | 4112.98(3935.65to4258.70) | 1.06(-0.53to2.63) |  | 2577.88(2416.87to2725.53) | 1.12(-1.24to3.54) |  | 1535.10(1489.40to1589.28) | 0.96(-0.72to2.80) |
| **Grenada** | 19175.10(18492.58to19959.88) | 3.33(1.10to5.67) |  | 550.25(507.95to591.93) | 4.68(1.34to8.32) |  | 18624.85(17964.69to19406.29) | 3.29(0.99to5.72) |
| **Guam** | 6608.19(6363.99to6869.97) | 3.65(1.27to5.76) |  | 209.38(193.92to223.93) | 3.47(0.57to6.18) |  | 6398.81(6162.48to6664.71) | 3.66(1.18to5.82) |
| **Guatemala** | 19407.14(18783.85to20085.25) | -0.17(-2.40to2.00) |  | 687.70(632.77to741.36) | -0.48(-3.95to3.34) |  | 18719.44(18090.98to19400.20) | -0.16(-2.52to2.12) |
| **Guinea** | 17464.44(16813.82to18176.73) | 2.27(0.01to4.37) |  | 390.27(362.88to417.49) | 1.21(-1.59to3.89) |  | 17074.16(16433.67to17783.15) | 2.29(0.00to4.49) |
| **Guinea-Bissau** | 17458.64(16822.82to18242.48) | 2.38(0.12to4.62) |  | 390.56(362.23to418.30) | 1.52(-1.10to4.25) |  | 17068.08(16434.19to17829.68) | 2.40(0.10to4.70) |
| **Guyana** | 18817.45(18150.73to19565.41) | 1.30(-0.86to3.31) |  | 541.51(498.26to585.26) | 3.15(0.35to6.14) |  | 18275.94(17606.70to19015.11) | 1.25(-1.01to3.35) |
| **Haiti** | 18698.62(18036.15to19434.58) | 1.10(-1.12to3.35) |  | 540.50(499.56to581.76) | 2.96(-0.28to6.14) |  | 18158.12(17515.06to18891.01) | 1.04(-1.25to3.34) |
| **Honduras** | 19391.24(18757.41to20109.82) | -0.32(-2.73to2.00) |  | 692.54(637.31to751.64) | 0.38(-2.90to3.70) |  | 18698.71(18076.58to19408.77) | -0.34(-2.80to2.09) |
| **Hungary** | 16115.39(15630.88to16669.20) | 1.45(-0.50to3.49) |  | 452.41(421.67to483.78) | 2.50(-0.25to5.31) |  | 15662.97(15185.01to16219.37) | 1.42(-0.57to3.52) |
| **Iceland** | 19233.40(18589.73to19874.73) | 1.72(-0.71to4.25) |  | 1138.64(1067.40to1209.53) | 4.95(2.33to7.55) |  | 18094.76(17456.86to18741.20) | 1.52(-1.07to4.21) |
| **India** | 22072.27(21523.03to22766.31) | 5.55(4.82to6.24) |  | 400.29(374.05to427.27) | 8.45(7.71to9.22) |  | 21671.99(21116.58to22364.99) | 5.50(4.76to6.21) |
| **Indonesia** | 2120.99(2058.21to2193.64) | 5.94(5.33to6.55) |  | 334.45(311.09to356.38) | 6.59(5.72to7.48) |  | 1786.55(1730.22to1856.07) | 5.82(5.10to6.55) |
| **Iran (Islamic Republic of)** | 12494.83(12175.12to12885.75) | 1.02(0.49to1.54) |  | 395.51(369.14to421.66) | 0.01(-0.59to0.73) |  | 12099.31(11777.19to12492.28) | 1.05(0.51to1.59) |
| **Iraq** | 11791.32(11377.38to12275.67) | 1.14(-1.31to3.54) |  | 316.89(294.88to339.51) | 0.49(-2.33to3.19) |  | 11474.43(11057.20to11961.58) | 1.16(-1.37to3.58) |
| **Ireland** | 23554.13(22706.10to24407.65) | 0.89(-1.42to3.10) |  | 544.75(509.39to580.19) | 6.10(2.92to9.24) |  | 23009.37(22171.56to23859.84) | 0.78(-1.63to3.03) |
| **Israel** | 23611.78(22825.06to24434.21) | 1.51(-0.64to3.91) |  | 559.98(522.10to595.97) | 10.77(7.56to14.12) |  | 23051.80(22268.52to23872.86) | 1.30(-0.84to3.76) |
| **Italy** | 15899.75(15411.45to16469.94) | 0.25(-0.81to1.32) |  | 387.74(361.66to412.60) | -4.26(-6.05to-2.36) |  | 15512.01(15019.10to16076.91) | 0.37(-0.72to1.46) |
| **Jamaica** | 18847.19(18159.61to19574.05) | 1.68(-0.52to4.01) |  | 544.67(503.15to584.87) | 3.80(0.79to7.02) |  | 18302.52(17613.55to19031.04) | 1.62(-0.58to4.02) |
| **Japan** | 20081.81(19431.78to20762.10) | 6.05(5.24to6.98) |  | 1176.98(1094.81to1261.23) | 6.00(4.82to7.25) |  | 18904.83(18289.05to19590.14) | 6.05(5.21to7.05) |
| **Jordan** | 12521.05(12112.33to13009.90) | 1.42(-0.64to3.51) |  | 444.16(412.63to475.03) | 5.36(2.53to8.09) |  | 12076.89(11655.22to12573.42) | 1.28(-0.87to3.46) |
| **Kazakhstan** | 9001.73(8700.05to9369.63) | 1.28(-1.08to3.74) |  | 405.14(376.66to433.57) | 3.35(0.69to6.22) |  | 8596.60(8283.65to8962.99) | 1.18(-1.25to3.74) |
| **Kenya** | 12656.26(12328.15to13041.84) | 2.13(1.75to2.53) |  | 264.28(246.36to282.66) | 1.83(1.36to2.37) |  | 12391.98(12063.25to12782.33) | 2.13(1.74to2.54) |
| **Kiribati** | 6478.20(6241.73to6739.00) | 2.81(0.51to5.03) |  | 207.59(192.43to223.09) | 3.45(0.54to6.37) |  | 6270.61(6033.32to6532.57) | 2.78(0.42to5.07) |
| **Kuwait** | 12416.43(12018.65to12905.15) | 0.77(-1.32to2.89) |  | 319.63(296.68to341.73) | -0.69(-3.36to2.29) |  | 12096.80(11696.54to12590.75) | 0.81(-1.35to2.98) |
| **Kyrgyzstan** | 9033.79(8689.75to9396.75) | 1.49(-0.76to3.93) |  | 406.69(377.46to435.10) | 3.41(0.30to6.13) |  | 8627.10(8290.09to8989.11) | 1.40(-0.98to3.85) |
| **Lao People's Democratic Republic** | 5620.00(5435.31to5833.40) | 5.38(3.10to7.45) |  | 326.32(304.19to349.76) | 6.88(3.89to9.83) |  | 5293.68(5107.38to5517.23) | 5.29(2.87to7.47) |
| **Latvia** | 12063.25(11652.33to12533.94) | 1.94(-0.07to4.14) |  | 1069.04(997.05to1138.30) | 4.30(1.37to7.04) |  | 10994.21(10575.57to11470.55) | 1.72(-0.42to4.03) |
| **Lebanon** | 12336.95(11904.56to12846.92) | 1.02(-1.08to3.06) |  | 315.52(293.32to338.72) | 0.39(-2.30to3.17) |  | 12021.42(11590.95to12536.56) | 1.04(-1.12to3.14) |
| **Lesotho** | 29558.79(28539.75to30627.18) | 1.73(-0.44to3.77) |  | 678.35(633.75to721.69) | 1.97(-0.49to4.63) |  | 28880.44(27862.78to29946.61) | 1.73(-0.49to3.84) |
| **Liberia** | 17544.08(16927.21to18280.33) | 2.60(0.44to5.04) |  | 391.72(364.16to419.76) | 1.50(-1.22to4.10) |  | 17152.36(16538.02to17886.37) | 2.63(0.43to5.13) |
| **Libya** | 12356.87(11900.94to12845.83) | 0.80(-1.37to3.07) |  | 317.26(295.02to339.23) | -0.11(-2.61to2.56) |  | 12039.61(11597.24to12531.57) | 0.82(-1.41to3.17) |
| **Lithuania** | 11955.39(11547.01to12434.47) | 1.85(-0.37to3.96) |  | 979.24(915.08to1048.17) | 3.21(0.44to5.98) |  | 10976.15(10568.49to11455.38) | 1.73(-0.62to4.01) |
| **Luxembourg** | 25475.24(24561.71to26346.92) | 0.97(-1.35to3.17) |  | 395.92(369.82to422.57) | 2.69(0.12to5.61) |  | 25079.32(24180.55to25955.51) | 0.95(-1.39to3.19) |
| **Madagascar** | 12517.28(12085.64to12964.08) | 1.95(-0.04to4.00) |  | 255.05(236.84to272.89) | 1.67(-1.14to4.53) |  | 12262.23(11846.67to12712.02) | 1.95(-0.06to4.03) |
| **Malawi** | 12510.39(12092.00to12949.83) | 2.00(-0.06to4.14) |  | 255.04(236.87to273.47) | 1.70(-1.03to4.68) |  | 12255.35(11827.94to12697.10) | 2.01(-0.14to4.20) |
| **Malaysia** | 5740.15(5539.96to5949.99) | 6.17(3.90to8.53) |  | 358.96(335.10to383.09) | 13.19(9.92to16.77) |  | 5381.20(5182.85to5586.79) | 5.73(3.30to8.18) |
| **Maldives** | 5847.41(5651.61to6065.53) | 7.63(5.54to9.95) |  | 343.82(319.86to367.51) | 10.20(7.20to13.21) |  | 5503.59(5302.29to5723.55) | 7.48(5.28to9.95) |
| **Mali** | 17492.11(16829.65to18239.51) | 2.50(0.15to4.97) |  | 391.76(364.43to418.60) | 1.54(-1.19to4.26) |  | 17100.35(16418.57to17850.57) | 2.52(0.09to5.04) |
| **Malta** | 23569.49(22804.95to24433.02) | 1.47(-0.74to3.76) |  | 818.17(767.08to871.41) | 5.83(2.94to8.82) |  | 22751.32(21980.78to23634.04) | 1.31(-0.95to3.69) |
| **Marshall Islands** | 6575.32(6350.69to6846.46) | 3.59(1.50to5.99) |  | 209.13(193.71to224.62) | 3.80(1.00to6.45) |  | 6366.19(6143.19to6633.84) | 3.59(1.43to6.05) |
| **Mauritania** | 17555.08(16883.30to18332.90) | 2.52(0.17to4.96) |  | 390.71(363.38to418.84) | 1.44(-1.50to4.46) |  | 17164.38(16488.66to17945.72) | 2.54(0.13to5.04) |
| **Mauritius** | 5625.79(5435.35to5832.39) | 5.01(2.94to7.06) |  | 337.54(314.73to358.44) | 9.79(6.86to12.91) |  | 5288.25(5104.28to5490.01) | 4.72(2.60to6.85) |
| **Mexico** | 21237.77(20641.48to21913.68) | 3.92(3.36to4.51) |  | 907.20(837.96to976.94) | 6.60(5.50to7.69) |  | 20330.57(19729.65to20993.98) | 3.81(3.22to4.41) |
| **Micronesia (Federated States of)** | 6577.40(6335.88to6836.93) | 3.17(0.95to5.44) |  | 208.65(193.58to223.25) | 3.61(0.91to6.25) |  | 6368.75(6119.62to6629.53) | 3.15(0.86to5.50) |
| **Monaco** | 23531.71(22705.39to24361.81) | 0.98(-1.31to3.22) |  | 522.26(486.93to559.13) | 2.83(-0.03to5.69) |  | 23009.44(22198.76to23853.09) | 0.94(-1.40to3.26) |
| **Mongolia** | 9056.46(8744.38to9400.79) | 0.97(-1.39to3.24) |  | 406.35(376.56to433.63) | 2.84(0.11to5.80) |  | 8650.11(8339.44to8991.42) | 0.88(-1.57to3.21) |
| **Montenegro** | 16107.61(15613.65to16604.34) | 1.08(-0.90to3.08) |  | 454.04(423.63to485.61) | 2.46(-0.22to5.28) |  | 15653.57(15163.64to16141.55) | 1.04(-1.00to3.10) |
| **Morocco** | 12339.20(11927.05to12851.00) | 1.14(-0.81to3.29) |  | 316.70(294.40to339.07) | 0.71(-1.97to3.48) |  | 12022.51(11609.65to12541.47) | 1.15(-0.85to3.37) |
| **Mozambique** | 12582.13(12163.00to13028.72) | 2.83(0.89to4.85) |  | 261.23(242.32to279.80) | 3.45(0.63to6.26) |  | 12320.91(11898.78to12763.43) | 2.81(0.81to4.87) |
| **Myanmar** | 5582.56(5394.19to5781.39) | 4.35(2.25to6.64) |  | 326.05(303.40to348.56) | 6.36(3.48to9.11) |  | 5256.52(5069.91to5455.38) | 4.23(2.00to6.66) |
| **Namibia** | 29493.25(28465.41to30550.59) | 1.42(-0.70to3.64) |  | 680.56(635.62to724.81) | 1.90(-0.67to4.79) |  | 28812.69(27785.56to29863.75) | 1.41(-0.76to3.70) |
| **Nauru** | 6524.74(6296.65to6785.57) | 2.44(0.30to4.63) |  | 209.54(194.25to224.12) | 3.38(0.77to6.10) |  | 6315.21(6084.45to6578.26) | 2.41(0.20to4.69) |
| **Nepal** | 23101.97(22371.63to24037.58) | 5.30(2.80to7.69) |  | 383.01(356.10to410.08) | 5.95(3.23to9.03) |  | 22718.96(21992.35to23663.77) | 5.29(2.77to7.72) |
| **Netherlands** | 23444.96(22593.13to24328.63) | 1.13(-1.27to3.40) |  | 525.66(490.44to561.36) | 3.35(0.27to6.48) |  | 22919.30(22082.82to23792.71) | 1.08(-1.34to3.43) |
| **New Zealand** | 32523.33(31553.32to33502.53) | 4.10(2.34to5.86) |  | 2564.18(2421.17to2708.06) | 4.58(2.15to7.01) |  | 29959.16(29017.05to30936.72) | 4.06(2.07to5.97) |
| **Nicaragua** | 19505.87(18879.29to20222.93) | 0.51(-1.62to2.79) |  | 694.78(640.61to751.02) | 0.82(-2.96to4.54) |  | 18811.09(18187.65to19512.24) | 0.50(-1.69to2.88) |
| **Niger** | 17479.61(16842.39to18260.49) | 2.17(-0.25to4.54) |  | 391.19(363.89to416.67) | 1.27(-1.48to3.89) |  | 17088.42(16459.87to17867.03) | 2.19(-0.29to4.63) |
| **Nigeria** | 17695.05(17218.24to18281.56) | 1.42(0.98to1.84) |  | 397.01(370.22to424.38) | 0.97(0.47to1.55) |  | 17298.04(16826.26to17891.85) | 1.43(0.98to1.86) |
| **Niue** | 6553.49(6314.18to6829.71) | 3.49(1.02to5.98) |  | 209.97(195.20to225.31) | 3.97(1.18to7.06) |  | 6343.52(6106.58to6622.04) | 3.47(0.94to6.00) |
| **North Macedonia** | 16145.03(15663.27to16677.65) | 1.18(-1.00to3.31) |  | 455.10(422.65to486.60) | 2.53(-0.19to5.33) |  | 15689.93(15212.92to16222.57) | 1.14(-1.08to3.31) |
| **Northern Mariana Islands** | 6659.71(6416.15to6935.72) | 3.48(1.28to5.72) |  | 229.22(213.60to244.87) | 5.41(2.30to8.66) |  | 6430.49(6189.03to6708.11) | 3.41(1.13to5.78) |
| **Norway** | 23758.31(23012.56to24554.53) | 1.14(0.47to1.79) |  | 407.54(381.58to435.25) | 3.67(2.65to4.76) |  | 23350.77(22604.07to24145.51) | 1.10(0.42to1.75) |
| **Oman** | 12548.39(12075.12to13052.64) | 1.62(-0.75to3.92) |  | 331.83(308.61to354.87) | 2.43(-0.37to5.55) |  | 12216.57(11754.09to12714.03) | 1.60(-0.84to3.93) |
| **Pakistan** | 22528.37(21908.92to23243.34) | 4.94(3.51to6.30) |  | 384.43(358.49to412.04) | 4.06(2.30to5.65) |  | 22143.94(21517.99to22865.81) | 4.96(3.50to6.34) |
| **Palau** | 6613.05(6374.65to6878.50) | 4.26(1.99to6.59) |  | 210.43(195.30to225.71) | 4.25(1.62to6.99) |  | 6402.62(6164.29to6664.31) | 4.26(1.91to6.66) |
| **Palestine** | 12348.02(11910.98to12844.51) | 1.24(-0.67to3.26) |  | 316.36(294.31to337.88) | 0.69(-2.12to3.52) |  | 12031.66(11595.16to12525.78) | 1.25(-0.69to3.35) |
| **Panama** | 19591.39(18971.49to20269.95) | 0.22(-2.01to2.84) |  | 698.69(642.35to754.03) | 0.48(-3.34to4.16) |  | 18892.71(18277.22to19561.18) | 0.21(-2.10to2.85) |
| **Papua New Guinea** | 6559.13(6325.87to6821.37) | 3.22(0.86to5.62) |  | 209.17(193.99to223.36) | 3.77(1.22to6.52) |  | 6349.95(6111.32to6610.07) | 3.20(0.75to5.68) |
| **Paraguay** | 19291.75(18734.16to19972.79) | 0.46(-1.70to2.64) |  | 518.43(479.49to558.94) | 1.67(-1.08to4.92) |  | 18773.32(18216.87to19451.25) | 0.43(-1.79to2.68) |
| **Peru** | 18937.36(18341.29to19563.74) | 1.19(-0.99to3.33) |  | 524.84(485.15to566.02) | 5.85(2.51to9.15) |  | 18412.52(17830.28to19051.53) | 1.06(-1.19to3.28) |
| **Philippines** | 8249.41(8027.45to8511.65) | 7.43(6.86to7.98) |  | 363.35(339.15to386.60) | 12.27(11.57to12.94) |  | 7886.06(7663.19to8144.95) | 7.22(6.62to7.78) |
| **Poland** | 3880.18(3808.19to3958.28) | -33.94(-35.65to-32.29) |  | 301.70(290.61to313.25) | -16.25(-20.27to-12.18) |  | 3578.48(3511.51to3656.80) | -35.10(-36.92to-33.39) |
| **Portugal** | 12302.59(11888.56to12749.73) | 2.04(-0.52to4.64) |  | 364.82(341.11to387.96) | 7.82(4.93to10.79) |  | 11937.77(11530.13to12385.27) | 1.87(-0.73to4.52) |
| **Puerto Rico** | 18933.22(18246.97to19675.45) | 2.39(0.03to4.65) |  | 553.41(513.79to594.71) | 4.71(1.44to8.04) |  | 18379.81(17695.26to19118.47) | 2.32(-0.07to4.60) |
| **Qatar** | 12646.44(12189.08to13160.64) | 1.72(-0.44to4.12) |  | 333.52(310.26to356.01) | 2.21(-1.00to5.47) |  | 12312.92(11857.21to12831.82) | 1.71(-0.51to4.16) |
| **Republic of Korea** | 18376.35(17697.81to19130.64) | 0.49(-2.14to3.00) |  | 1038.90(964.18to1117.97) | 0.92(-2.58to4.27) |  | 17337.46(16641.52to18094.54) | 0.46(-2.36to3.19) |
| **Republic of Moldova** | 11954.05(11526.75to12416.11) | 1.66(-0.34to4.08) |  | 1020.77(952.08to1089.18) | 3.80(1.26to6.44) |  | 10933.28(10515.43to11398.59) | 1.46(-0.73to4.08) |
| **Romania** | 21783.84(21152.89to22445.51) | 2.05(0.00to4.00) |  | 629.05(585.92to670.14) | 3.74(0.99to6.49) |  | 21154.79(20516.79to21833.14) | 2.00(-0.13to4.03) |
| **Russian Federation** | 12108.58(11802.68to12468.93) | 1.96(1.61to2.30) |  | 1104.18(1034.03to1174.14) | 8.24(7.37to9.17) |  | 11004.41(10708.44to11369.27) | 1.37(1.01to1.73) |
| **Rwanda** | 12527.86(12113.36to13000.26) | 2.18(0.13to4.13) |  | 255.47(237.59to274.23) | 1.86(-0.73to4.57) |  | 12272.39(11849.05to12759.15) | 2.18(0.11to4.19) |
| **Saint Kitts and Nevis** | 19022.13(18313.04to19788.23) | 2.20(-0.02to4.48) |  | 557.18(514.47to600.00) | 4.39(1.35to7.44) |  | 18464.95(17757.38to19222.61) | 2.14(-0.18to4.48) |
| **Saint Lucia** | 18944.06(18269.56to19660.77) | 2.31(0.06to4.35) |  | 554.71(513.98to594.97) | 5.75(2.55to9.21) |  | 18389.35(17721.65to19109.07) | 2.21(-0.11to4.30) |
| **Saint Vincent and the Grenadines** | 19019.89(18328.72to19761.46) | 2.15(-0.05to4.46) |  | 551.24(511.18to591.86) | 4.15(0.74to7.84) |  | 18468.64(17793.55to19213.47) | 2.09(-0.17to4.47) |
| **Samoa** | 6573.27(6333.21to6853.96) | 2.94(0.51to5.23) |  | 209.38(193.94to224.97) | 3.44(0.75to6.19) |  | 6363.90(6124.34to6641.57) | 2.92(0.42to5.29) |
| **San Marino** | 23406.52(22562.11to24241.89) | 0.14(-1.95to2.36) |  | 517.06(482.61to552.32) | 1.33(-1.48to4.55) |  | 22889.46(22047.81to23726.23) | 0.12(-2.03to2.42) |
| **Sao Tome and Principe** | 17585.83(16965.11to18285.43) | 2.70(0.23to5.01) |  | 392.77(365.07to418.93) | 1.64(-1.02to4.46) |  | 17193.05(16562.26to17892.10) | 2.72(0.18to5.09) |
| **Saudi Arabia** | 12452.78(12006.58to12943.90) | 1.09(-1.04to3.32) |  | 322.58(299.26to345.70) | 0.54(-2.18to3.33) |  | 12130.20(11682.77to12623.74) | 1.10(-1.04to3.33) |
| **Senegal** | 17515.50(16913.97to18233.98) | 2.39(0.19to4.71) |  | 391.00(362.91to418.49) | 1.36(-1.39to4.04) |  | 17124.51(16521.85to17841.25) | 2.41(0.15to4.78) |
| **Serbia** | 13968.20(13511.39to14462.05) | 2.03(-0.06to4.29) |  | 481.59(449.05to515.09) | 4.13(0.99to7.23) |  | 13486.61(13029.98to13981.77) | 1.96(-0.21to4.36) |
| **Seychelles** | 5691.81(5498.81to5912.75) | 5.92(3.72to8.11) |  | 330.12(306.47to352.80) | 7.06(4.29to9.80) |  | 5361.69(5174.39to5574.77) | 5.85(3.51to8.26) |
| **Sierra Leone** | 17510.93(16885.22to18246.88) | 2.54(0.26to4.98) |  | 391.17(364.40to419.51) | 1.39(-1.43to4.11) |  | 17119.75(16492.37to17867.42) | 2.56(0.21to5.05) |
| **Singapore** | 18344.35(17628.32to19078.37) | 0.33(-2.11to2.86) |  | 1085.56(1009.58to1161.23) | 1.90(-0.93to5.15) |  | 17258.79(16559.63to18016.35) | 0.24(-2.40to2.85) |
| **Slovakia** | 22386.64(21735.28to23072.77) | 1.07(-0.68to3.00) |  | 511.25(475.77to546.00) | 2.15(-0.45to5.03) |  | 21875.39(21226.84to22569.44) | 1.05(-0.73to3.01) |
| **Slovenia** | 18574.50(18005.32to19190.05) | 1.51(-0.41to3.47) |  | 459.63(427.43to489.25) | 2.45(-0.27to5.37) |  | 18114.87(17535.21to18731.58) | 1.49(-0.50to3.51) |
| **Solomon Islands** | 6556.05(6318.87to6848.86) | 2.86(0.66to5.27) |  | 208.98(193.86to223.22) | 3.38(0.72to6.13) |  | 6347.07(6105.47to6635.67) | 2.85(0.57to5.35) |
| **Somalia** | 12489.74(12069.75to12919.89) | 2.08(0.14to4.09) |  | 254.35(235.94to271.73) | 1.55(-1.15to4.26) |  | 12235.40(11827.45to12673.45) | 2.09(0.12to4.14) |
| **South Africa** | 29464.98(28584.70to30417.79) | 1.60(0.70to2.47) |  | 694.79(649.78to738.27) | 2.89(1.82to3.96) |  | 28770.19(27879.19to29734.43) | 1.57(0.65to2.46) |
| **South Sudan** | 12521.37(12089.88to12958.67) | 1.76(-0.30to3.74) |  | 255.21(237.02to272.94) | 1.61(-1.00to4.44) |  | 12266.16(11835.33to12705.91) | 1.77(-0.38to3.81) |
| **Spain** | 23590.35(22760.70to24480.45) | 1.14(-1.15to3.45) |  | 530.31(496.06to567.21) | 4.63(1.87to7.72) |  | 23060.04(22232.74to23963.97) | 1.07(-1.30to3.45) |
| **Sri Lanka** | 5577.52(5381.42to5774.99) | 3.84(1.81to5.97) |  | 326.67(303.78to348.86) | 6.01(3.36to9.02) |  | 5250.85(5060.40to5452.23) | 3.71(1.56to5.91) |
| **Sudan** | 12340.30(11897.78to12841.51) | 1.09(-1.00to3.33) |  | 316.41(293.98to338.39) | 0.57(-2.16to3.39) |  | 12023.89(11572.88to12526.09) | 1.10(-1.03to3.43) |
| **Suriname** | 19010.03(18319.52to19755.74) | 1.97(-0.21to4.43) |  | 548.13(507.61to590.15) | 3.73(0.68to7.03) |  | 18461.90(17794.01to19211.41) | 1.92(-0.30to4.47) |
| **Sweden** | 23000.51(22225.65to23840.72) | 0.87(-1.25to2.80) |  | 348.79(325.94to374.17) | 3.05(0.70to5.42) |  | 22651.73(21874.26to23491.64) | 0.84(-1.30to2.81) |
| **Switzerland** | 25964.80(25043.74to26861.63) | 0.76(-1.44to2.94) |  | 468.91(437.50to501.14) | 2.91(-0.10to5.72) |  | 25495.89(24573.65to26395.49) | 0.72(-1.50to2.94) |
| **Syrian Arab Republic** | 12307.16(11888.12to12795.72) | 0.68(-1.57to2.80) |  | 314.79(292.90to337.36) | -0.39(-2.85to2.36) |  | 11992.37(11574.12to12483.07) | 0.71(-1.57to2.89) |
| **Taiwan (Province of China)** | 9001.71(8706.39to9298.65) | 9.53(7.12to12.19) |  | 230.51(214.84to246.27) | 26.27(22.27to30.13) |  | 8771.19(8472.05to9065.79) | 9.15(6.69to11.83) |
| **Tajikistan** | 9075.49(8738.44to9461.11) | 1.59(-0.68to4.10) |  | 408.31(380.06to435.94) | 3.39(0.67to6.33) |  | 8667.17(8334.39to9052.36) | 1.51(-0.92to4.12) |
| **Thailand** | 5665.93(5477.83to5861.32) | 5.02(2.97to7.23) |  | 327.37(304.07to350.28) | 6.35(3.50to9.47) |  | 5338.56(5144.27to5537.16) | 4.94(2.82to7.32) |
| **Timor-Leste** | 5615.58(5432.91to5815.62) | 4.21(2.01to6.62) |  | 326.24(303.80to348.65) | 6.06(3.37to8.86) |  | 5289.34(5104.99to5492.89) | 4.10(1.77to6.58) |
| **Togo** | 17478.97(16868.25to18224.14) | 2.34(0.10to4.69) |  | 390.81(363.22to419.11) | 1.40(-1.37to3.98) |  | 17088.16(16474.04to17839.62) | 2.37(0.10to4.77) |
| **Tokelau** | 6556.48(6317.19to6804.11) | 4.10(1.84to6.42) |  | 209.79(194.04to224.70) | 4.13(1.55to6.91) |  | 6346.69(6111.57to6599.32) | 4.10(1.77to6.53) |
| **Tonga** | 6543.77(6306.43to6825.23) | 3.31(1.12to5.46) |  | 208.84(194.07to223.63) | 3.59(0.80to6.22) |  | 6334.94(6090.84to6622.74) | 3.30(0.97to5.52) |
| **Trinidad and Tobago** | 18916.30(18277.58to19713.69) | 1.66(-0.63to4.05) |  | 554.64(515.11to595.17) | 5.36(1.85to8.99) |  | 18361.67(17714.90to19144.79) | 1.56(-0.77to4.03) |
| **Tunisia** | 12348.82(11936.00to12838.12) | 1.01(-1.03to3.00) |  | 316.00(293.09to337.20) | 0.18(-2.44to3.04) |  | 12032.81(11616.59to12522.88) | 1.03(-1.09to3.11) |
| **Turkey** | 12243.73(11833.98to12728.96) | 1.16(-1.04to3.35) |  | 197.78(183.51to212.01) | 0.43(-1.97to3.24) |  | 12045.95(11635.18to12542.24) | 1.17(-1.07to3.42) |
| **Turkmenistan** | 9128.78(8818.31to9508.47) | 2.51(0.41to4.86) |  | 408.30(379.50to436.79) | 3.80(1.05to6.80) |  | 8720.48(8410.55to9095.53) | 2.45(0.28to4.91) |
| **Tuvalu** | 6563.32(6322.26to6807.97) | 5.03(2.76to7.24) |  | 210.00(195.28to225.19) | 4.91(2.27to7.95) |  | 6353.32(6115.30to6601.95) | 5.03(2.67to7.31) |
| **Uganda** | 12511.43(12079.36to12951.63) | 2.11(0.13to4.25) |  | 255.29(236.29to273.30) | 1.98(-0.66to4.53) |  | 12256.15(11829.10to12701.33) | 2.11(0.11to4.28) |
| **Ukraine** | 11993.90(11635.74to12411.22) | 1.46(-0.50to3.35) |  | 1013.48(944.57to1080.76) | 2.18(-0.32to4.98) |  | 10980.42(10618.28to11389.34) | 1.40(-0.72to3.53) |
| **United Arab Emirates** | 12556.59(12112.21to13095.13) | 1.29(-1.09to3.78) |  | 327.82(304.30to349.79) | 1.25(-1.81to4.35) |  | 12228.77(11781.70to12770.80) | 1.29(-1.17to3.88) |
| **United Kingdom** | 33018.44(32150.26to33954.80) | 7.85(7.35to8.37) |  | 1790.72(1685.88to1891.35) | 20.55(18.57to22.43) |  | 31227.72(30367.72to32164.26) | 7.21(6.69to7.70) |
| **United Republic of Tanzania** | 12530.29(12102.47to12978.15) | 2.33(0.33to4.34) |  | 256.00(237.65to274.05) | 2.06(-0.78to4.93) |  | 12274.29(11840.09to12726.49) | 2.33(0.29to4.41) |
| **United States of America** | 4687.35(4516.39to4838.45) | 4.04(3.07to5.05) |  | 2985.00(2828.46to3135.85) | 5.36(3.81to6.83) |  | 1702.35(1657.85to1755.05) | 1.81(0.99to2.70) |
| **United States Virgin Islands** | 18838.32(18181.84to19603.37) | 1.96(-0.20to4.23) |  | 564.21(523.24to610.60) | 6.08(2.71to9.74) |  | 18274.10(17632.84to19036.83) | 1.84(-0.37to4.17) |
| **Uruguay** | 10544.93(10196.30to10935.41) | 1.45(-0.69to3.71) |  | 522.45(486.96to558.07) | 6.51(3.50to9.76) |  | 10022.47(9683.53to10399.67) | 1.20(-1.06to3.64) |
| **Uzbekistan** | 9043.46(8707.49to9423.21) | 1.43(-0.79to3.70) |  | 407.20(378.61to436.76) | 3.28(0.45to6.12) |  | 8636.26(8299.58to8998.00) | 1.34(-0.99to3.71) |
| **Vanuatu** | 6561.90(6327.31to6842.15) | 3.16(0.83to5.45) |  | 208.95(194.05to223.27) | 3.47(0.61to6.29) |  | 6352.95(6118.41to6630.19) | 3.15(0.78to5.50) |
| **Venezuela (Bolivarian Republic of)** | 19462.24(18816.41to20183.63) | -0.12(-2.39to2.48) |  | 694.05(639.53to749.06) | 0.35(-3.36to4.22) |  | 18768.20(18126.50to19484.63) | -0.14(-2.50to2.51) |
| **Viet Nam** | 11177.22(10728.99to11650.44) | 8.61(6.13to11.00) |  | 329.55(307.17to352.32) | 7.50(4.82to10.21) |  | 10847.67(10403.38to11325.24) | 8.64(6.11to11.12) |
| **Yemen** | 12331.27(11888.01to12839.26) | 1.00(-1.05to3.02) |  | 316.23(293.63to338.59) | 0.67(-2.24to3.44) |  | 12015.04(11581.56to12517.69) | 1.01(-1.09to3.09) |
| **Zambia** | 12556.40(12125.29to13014.32) | 2.21(0.18to4.23) |  | 256.66(238.26to274.52) | 1.91(-0.79to4.70) |  | 12299.74(11868.21to12760.11) | 2.22(0.09to4.25) |
| **Zimbabwe** | 29457.79(28475.19to30548.70) | 1.03(-1.14to3.21) |  | 679.97(633.63to726.02) | 1.35(-0.99to3.87) |  | 28777.82(27797.75to29890.41) | 1.02(-1.19to3.24) |
