# Supplementary material for: Global Burden of Bacterial Skin Diseases: A Systematic Analysis Combined With Sociodemographic Index, 1990–2019
Source: Front Med (Lausanne). 2022 Apr 25;9:861115. doi: 10.3389/fmed.2022.861115 (PMC9084187; doi:10.3389/fmed.2022.861115)
Supplement: Supplementary file 6 [file Table_6.docx]

S6 Table DALYs of major bacterial skin diseases in 2019, and change of Age-standardized DALYs during the periods 1990-2019 for both sexes in 204 countries.

| **DALYs (Disability-Adjusted Life Years)** | | | | | | | | |
| --- | --- | --- | --- | --- | --- | --- | --- | --- |
|  | **Bacterial skin diseases** | |  | **Cellulitis** | |  | **Pyoderma** | |
|  | **Number of DALYs, 2019** | **Annualised rate of change of age-standardized DALYs(1990-2019)(%)** |  | **Number of DALYs, 2019** | **Annualised rate of change of age-standardized DALYs(1990-2019)(%)** |  | **Number of DALYs, 2019** | **Annualised rate of change of age-standardized DALYs(1990-2019)(%)** |
| **Afghanistan** | 17.10(10.78to27.49) | 13.32(-11.19to47.50) |  | 2.97(1.87to6.43) | -21.03(-46.78to49.75) |  | 14.12(8.61to21.72) | 24.73(-6.32to71.12) |
| **Albania** | 7.84(5.39to11.48) | -51.08(-65.82to-13.46) |  | 1.85(1.32to2.54) | -38.13(-59.59to5.36) |  | 5.99(3.80to9.56) | -54.05(-70.59to-14.05) |
| **Algeria** | 9.42(6.73to14.39) | -1.37(-19.17to20.32) |  | 1.58(1.12to2.67) | -26.11(-49.59to18.33) |  | 7.84(5.42to11.89) | 5.77(-16.11to36.32) |
| **American Samoa** | 176.43(124.65to229.22) | 25.18(-20.12to81.85) |  | 11.39(4.09to31.97) | -17.88(-61.67to57.10) |  | 165.04(108.62to221.15) | 29.88(-17.86to89.18) |
| **Andorra** | 9.22(5.82to14.85) | 8.40(-4.80to24.85) |  | 2.59(1.75to3.50) | 7.56(-13.96to34.59) |  | 6.63(3.59to12.21) | 8.74(-3.43to26.24) |
| **Angola** | 39.59(30.45to51.87) | -5.32(-32.86to34.48) |  | 9.97(3.83to16.44) | -20.29(-54.41to48.75) |  | 29.62(20.98to41.68) | 1.07(-31.32to58.61) |
| **Antigua and Barbuda** | 47.57(26.40to81.45) | 144.60(50.11to251.41) |  | 14.23(7.63to25.50) | 81.74(20.58to164.46) |  | 33.34(17.62to56.56) | 186.98(63.29to333.80) |
| **Argentina** | 43.76(21.42to56.50) | 312.61(92.15to548.51) |  | 9.67(4.69to12.68) | 188.10(21.57to389.04) |  | 34.09(16.51to45.92) | 370.26(116.71to640.40) |
| **Armenia** | 9.22(5.62to12.82) | 67.43(-0.10to147.80) |  | 2.76(1.58to3.75) | 37.75(-24.09to101.11) |  | 6.46(3.70to9.55) | 84.41(6.59to192.84) |
| **Australia** | 26.18(15.81to34.83) | 67.19(3.33to112.70) |  | 14.33(7.73to18.08) | 87.83(-7.64to150.84) |  | 11.85(7.34to18.38) | 47.59(11.93to88.78) |
| **Austria** | 12.66(8.17to19.72) | 29.60(6.08to58.22) |  | 2.69(1.88to3.71) | 2.91(-18.03to23.12) |  | 9.97(5.94to16.62) | 39.34(11.43to88.66) |
| **Azerbaijan** | 6.91(5.15to9.60) | -7.35(-21.17to5.74) |  | 1.89(1.37to2.56) | -13.61(-29.19to7.71) |  | 5.02(3.50to7.46) | -4.76(-21.53to14.49) |
| **Bahamas** | 31.26(18.77to50.05) | 41.36(2.65to89.98) |  | 7.12(4.38to11.71) | 13.54(-26.43to67.63) |  | 24.14(14.12to38.61) | 52.38(9.78to111.77) |
| **Bahrain** | 119.88(96.26to147.42) | -64.71(-74.35to-36.46) |  | 45.38(23.27to66.46) | -72.86(-82.14to-49.69) |  | 74.50(53.19to103.00) | -56.81(-69.89to-23.88) |
| **Bangladesh** | 25.31(18.89to37.16) | -22.35(-46.95to18.61) |  | 2.55(1.65to5.28) | -27.10(-54.91to14.46) |  | 22.75(16.75to32.18) | -21.78(-47.08to21.21) |
| **Barbados** | 122.79(72.83to221.89) | 24.70(-7.17to70.99) |  | 33.81(17.87to70.41) | 2.00(-30.24to53.15) |  | 88.97(53.41to153.33) | 36.22(-2.53to90.10) |
| **Belarus** | 10.29(7.19to15.26) | 4.57(-12.08to28.58) |  | 5.45(3.91to8.48) | -3.00(-21.82to23.79) |  | 4.84(2.90to8.17) | 14.63(-1.92to45.13) |
| **Belgium** | 18.58(11.15to27.17) | 98.53(25.95to179.18) |  | 3.93(2.49to5.39) | 56.76(6.69to108.62) |  | 14.65(8.47to22.20) | 113.82(32.95to223.45) |
| **Belize** | 30.89(19.84to46.77) | 83.33(31.18to156.43) |  | 7.74(4.61to13.38) | 28.11(-13.29to88.00) |  | 23.15(14.17to35.75) | 114.23(49.11to217.94) |
| **Benin** | 58.18(40.67to79.35) | 1.74(-26.89to35.91) |  | 3.87(1.96to5.79) | -21.16(-49.86to47.86) |  | 54.31(36.83to75.06) | 3.89(-26.50to41.98) |
| **Bermuda** | 14.72(9.43to22.41) | 33.70(-6.52to83.40) |  | 1.74(1.18to2.48) | -2.86(-19.76to16.34) |  | 12.98(7.90to20.44) | 40.80(-5.70to101.24) |
| **Bhutan** | 37.53(14.84to54.91) | -1.28(-34.50to48.59) |  | 3.81(1.85to5.71) | -19.30(-53.87to45.30) |  | 33.73(12.90to49.75) | 1.28(-33.77to52.36) |
| **Bolivia (Plurinational State of)** | 19.13(14.27to25.12) | 0.94(-28.04to40.39) |  | 3.70(2.45to5.25) | -13.48(-38.51to37.96) |  | 15.44(11.20to20.88) | 5.14(-30.70to53.65) |
| **Bosnia and Herzegovina** | 9.98(7.24to13.83) | 16.28(-6.31to45.17) |  | 2.67(1.74to3.69) | 17.52(-16.37to55.31) |  | 7.30(4.88to10.89) | 15.84(-5.51to52.90) |
| **Botswana** | 33.41(20.85to54.77) | -13.68(-39.55to28.12) |  | 8.69(4.12to14.27) | -25.12(-50.04to17.53) |  | 24.72(13.82to42.48) | -8.78(-36.91to35.46) |
| **Brazil** | 62.20(31.73to79.87) | 80.06(-5.13to130.54) |  | 11.54(5.54to15.50) | 14.96(-41.18to52.97) |  | 50.66(24.68to66.41) | 106.73(13.15to163.32) |
| **Brunei Darussalam** | 27.93(21.59to34.05) | 58.70(22.59to108.99) |  | 10.53(7.59to13.73) | 42.19(-5.10to121.21) |  | 17.40(12.29to22.76) | 70.69(13.13to145.94) |
| **Bulgaria** | 13.46(9.11to19.39) | 34.68(-3.47to71.39) |  | 1.86(1.34to2.63) | -38.81(-53.11to-5.24) |  | 11.59(7.38to17.08) | 66.91(0.42to134.26) |
| **Burkina Faso** | 60.41(45.15to78.82) | 15.21(-15.16to57.56) |  | 4.13(1.87to6.84) | -12.56(-40.86to28.85) |  | 56.28(41.89to74.68) | 17.96(-14.72to65.42) |
| **Burundi** | 43.15(29.74to59.73) | -12.31(-44.15to29.90) |  | 11.26(3.89to19.76) | -19.59(-56.11to40.32) |  | 31.88(20.99to45.98) | -9.41(-46.66to44.11) |
| **Cabo Verde** | 35.26(27.84to46.41) | -18.68(-37.17to9.19) |  | 2.16(1.54to2.98) | -13.86(-36.09to23.27) |  | 33.10(26.17to43.86) | -18.97(-37.50to10.34) |
| **Cambodia** | 27.72(21.35to36.29) | -33.65(-52.04to-1.04) |  | 7.56(4.83to10.46) | -38.05(-57.27to-3.63) |  | 20.16(15.00to27.90) | -31.84(-51.68to3.55) |
| **Cameroon** | 61.94(44.14to87.01) | -4.21(-30.45to33.97) |  | 3.89(1.93to5.98) | -15.18(-40.94to25.48) |  | 58.05(40.35to82.69) | -3.37(-30.35to35.90) |
| **Canada** | 19.00(11.70to24.29) | 79.65(4.51to134.92) |  | 13.43(8.30to17.22) | 49.38(-12.46to95.91) |  | 5.57(2.96to8.66) | 251.27(70.21to387.73) |
| **Central African Republic** | 52.37(37.58to73.18) | -1.09(-27.96to36.45) |  | 14.45(4.56to24.50) | -7.17(-38.51to46.04) |  | 37.92(23.35to60.58) | 1.45(-27.00to44.29) |
| **Chad** | 52.42(39.49to67.77) | 5.10(-21.15to36.76) |  | 5.06(1.71to8.93) | -1.08(-30.13to41.43) |  | 47.35(36.20to62.46) | 5.81(-20.50to39.30) |
| **Chile** | 23.72(16.84to39.77) | -3.31(-19.90to31.66) |  | 13.25(9.49to24.22) | -18.02(-36.32to31.83) |  | 10.46(6.05to16.79) | 25.13(-15.28to67.81) |
| **China** | 5.16(3.97to7.45) | -79.06(-84.43to-55.85) |  | 0.85(0.65to1.23) | -84.18(-90.14to-63.52) |  | 4.31(3.19to6.51) | -77.63(-84.92to-52.71) |
| **Colombia** | 47.46(26.13to79.30) | 83.67(11.26to167.08) |  | 13.29(6.80to21.66) | 53.18(-20.22to131.04) |  | 34.18(18.61to57.67) | 99.08(29.45to205.11) |
| **Comoros** | 46.83(33.42to62.11) | -3.55(-33.02to69.32) |  | 10.57(3.66to17.86) | -16.43(-50.11to71.06) |  | 36.27(25.73to49.17) | 0.98(-32.34to81.19) |
| **Congo** | 46.40(33.81to62.74) | -14.19(-36.33to12.99) |  | 10.88(3.80to18.70) | -28.41(-55.14to23.89) |  | 35.52(24.19to51.06) | -8.62(-33.81to26.09) |
| **Cook Islands** | 23.97(17.25to30.60) | 20.03(-23.85to72.23) |  | 2.94(1.58to4.38) | -23.82(-49.80to32.67) |  | 21.04(15.15to27.15) | 30.51(-21.88to92.59) |
| **Costa Rica** | 25.17(17.53to38.18) | 44.76(13.75to87.24) |  | 14.44(9.35to23.62) | 43.60(8.33to94.06) |  | 10.72(6.93to17.15) | 46.36(15.43to110.19) |
| **Croatia** | 7.12(4.53to11.21) | 17.73(-7.74to41.26) |  | 1.40(0.99to1.91) | -10.50(-27.01to0.91) |  | 5.72(3.32to9.66) | 27.55(-4.04to65.38) |
| **Cuba** | 39.91(26.79to66.71) | 20.47(-11.62to72.94) |  | 14.79(9.32to28.21) | -1.36(-33.36to52.83) |  | 25.13(16.66to41.74) | 38.50(0.41to105.59) |
| **Cyprus** | 16.62(12.03to24.91) | -11.49(-34.51to17.67) |  | 3.06(2.17to4.36) | -15.69(-52.28to33.34) |  | 13.57(9.30to21.38) | -10.49(-32.65to18.40) |
| **Czechia** | 12.38(6.24to18.16) | 50.49(-12.45to107.39) |  | 4.45(1.89to6.23) | 39.72(-34.94to98.46) |  | 7.93(3.99to12.54) | 57.29(0.49to130.47) |
| **C么te d'Ivoire** | 50.08(35.33to67.23) | -8.19(-30.77to22.94) |  | 3.83(1.86to5.79) | -20.74(-47.57to28.98) |  | 46.25(32.30to63.10) | -6.97(-31.61to27.83) |
| **Democratic People's Republic of Korea** | 9.15(6.31to12.97) | -50.68(-69.34to-27.48) |  | 3.08(1.93to5.89) | -60.49(-77.64to-27.23) |  | 6.08(3.37to9.18) | -43.60(-72.49to-3.99) |
| **Democratic Republic of the Congo** | 41.02(30.09to57.22) | -12.84(-37.16to18.97) |  | 10.70(2.94to18.53) | -18.37(-49.84to51.48) |  | 30.32(20.16to48.11) | -10.70(-38.30to30.07) |
| **Denmark** | 18.22(9.24to26.35) | 71.88(3.83to142.70) |  | 1.85(1.23to2.52) | 15.93(-19.45to45.08) |  | 16.36(7.97to24.12) | 81.81(7.13to168.19) |
| **Djibouti** | 44.91(32.24to62.23) | 5.90(-23.42to47.85) |  | 10.55(3.45to18.33) | -12.37(-44.63to51.80) |  | 34.36(24.42to49.10) | 13.14(-18.61to63.13) |
| **Dominica** | 94.70(66.60to127.07) | 24.61(-15.63to85.17) |  | 22.93(14.01to32.98) | 8.18(-28.80to66.54) |  | 71.77(49.46to97.95) | 30.96(-14.25to97.53) |
| **Dominican Republic** | 15.17(11.17to20.73) | -33.64(-50.50to1.47) |  | 1.82(1.23to2.56) | -26.53(-50.49to12.89) |  | 13.35(9.61to18.81) | -34.50(-51.85to3.03) |
| **Ecuador** | 18.17(11.45to23.98) | 65.82(-7.30to117.76) |  | 3.08(1.61to4.40) | 55.27(-9.88to128.33) |  | 15.10(9.69to20.38) | 68.15(-7.59to129.49) |
| **Egypt** | 11.84(7.48to19.52) | -4.40(-27.49to31.72) |  | 1.81(1.10to3.33) | -14.78(-39.14to28.63) |  | 10.03(6.00to16.67) | -2.25(-25.86to35.66) |
| **El Salvador** | 28.78(21.70to37.52) | -13.00(-35.83to14.90) |  | 2.11(1.43to2.95) | -8.04(-28.45to19.10) |  | 26.67(19.80to35.06) | -13.37(-37.09to16.31) |
| **Equatorial Guinea** | 41.58(27.68to61.49) | -10.85(-44.24to35.79) |  | 8.65(3.13to15.37) | -37.96(-64.18to33.87) |  | 32.93(21.08to51.55) | 0.72(-39.63to69.80) |
| **Eritrea** | 52.26(37.62to72.94) | 7.62(-24.72to57.43) |  | 13.32(4.33to24.03) | -5.68(-39.32to58.74) |  | 38.94(26.31to56.07) | 13.08(-23.51to75.28) |
| **Estonia** | 22.75(14.46to39.95) | -18.50(-38.91to23.65) |  | 10.40(6.91to18.26) | -47.27(-63.00to-16.41) |  | 12.36(6.83to21.75) | 50.67(-8.34to144.78) |
| **Eswatini** | 65.13(45.07to90.56) | 3.45(-26.93to44.90) |  | 14.59(6.66to22.33) | -10.88(-42.05to35.34) |  | 50.55(34.00to71.43) | 8.48(-23.12to53.25) |
| **Ethiopia** | 42.41(34.63to52.95) | -32.34(-55.65to-2.12) |  | 10.26(3.65to17.25) | -41.27(-73.23to32.69) |  | 32.15(23.58to42.83) | -28.89(-57.77to10.71) |
| **Fiji** | 65.28(47.55to83.68) | 16.13(-20.95to66.44) |  | 11.69(7.35to16.09) | -26.35(-50.24to22.60) |  | 53.59(37.99to69.44) | 32.83(-13.99to93.31) |
| **Finland** | 14.21(9.38to22.06) | 19.17(-9.14to39.87) |  | 2.59(1.67to3.54) | 15.20(-29.87to45.34) |  | 11.62(7.38to18.87) | 20.09(-5.23to42.63) |
| **France** | 15.54(10.04to24.22) | 30.42(3.29to76.97) |  | 3.08(2.20to4.86) | -23.31(-37.32to11.33) |  | 12.46(7.30to19.81) | 57.68(13.29to135.69) |
| **Gabon** | 48.14(34.20to64.81) | 2.12(-28.40to48.11) |  | 9.98(3.90to16.44) | -23.91(-54.79to70.48) |  | 38.15(25.02to56.08) | 12.17(-25.08to79.59) |
| **Gambia** | 53.83(39.65to70.77) | 15.06(-19.09to65.79) |  | 3.87(1.95to5.94) | -9.26(-43.18to59.92) |  | 49.97(36.57to66.86) | 17.50(-17.66to72.26) |
| **Georgia** | 8.16(4.91to11.67) | 57.08(1.42to120.07) |  | 2.34(1.41to3.22) | 44.44(-11.16to103.50) |  | 5.81(3.17to8.70) | 62.83(3.60to139.02) |
| **Germany** | 18.27(11.71to25.90) | 53.65(4.31to105.86) |  | 4.40(2.57to5.74) | 36.09(-25.67to87.29) |  | 13.87(8.71to21.00) | 60.22(12.78to121.87) |
| **Ghana** | 48.91(30.04to65.23) | 8.50(-18.08to44.35) |  | 3.91(1.84to6.11) | -63.69(-77.40to12.26) |  | 45.01(27.57to60.39) | 31.13(-9.39to85.69) |
| **Greece** | 8.72(5.41to14.01) | 15.20(1.05to31.20) |  | 1.86(1.30to2.56) | -5.27(-19.98to9.46) |  | 6.86(3.90to12.04) | 22.38(3.81to52.06) |
| **Greenland** | 36.35(25.01to44.89) | 31.90(-11.88to66.95) |  | 11.60(7.32to15.57) | 3.62(-23.54to30.34) |  | 24.75(16.60to31.68) | 51.24(-11.02to109.03) |
| **Grenada** | 118.07(69.99to195.56) | 82.69(31.45to162.19) |  | 12.97(7.79to33.07) | -20.58(-51.28to42.04) |  | 105.10(60.76to175.40) | 117.60(42.80to226.13) |
| **Guam** | 78.76(54.80to98.31) | 113.04(5.59to204.98) |  | 3.57(2.35to6.15) | -14.63(-42.77to82.69) |  | 75.19(50.75to94.30) | 129.33(6.66to234.35) |
| **Guatemala** | 44.31(28.45to72.07) | 79.49(-0.10to220.16) |  | 10.08(6.18to18.17) | 2.91(-38.71to89.46) |  | 34.23(21.58to57.26) | 129.91(15.01to312.51) |
| **Guinea** | 55.78(41.75to73.23) | -5.69(-31.03to27.38) |  | 4.60(1.91to7.54) | -13.69(-44.49to31.41) |  | 51.17(37.96to67.75) | -4.90(-30.38to27.78) |
| **Guinea-Bissau** | 58.06(42.85to77.50) | -9.26(-33.86to28.69) |  | 4.83(2.04to8.14) | -24.02(-52.00to28.65) |  | 53.22(39.10to71.45) | -7.63(-33.36to32.76) |
| **Guyana** | 27.79(15.97to39.59) | 231.79(46.00to474.73) |  | 4.89(2.98to7.41) | 54.54(-18.72to141.91) |  | 22.90(12.52to33.09) | 339.52(64.23to893.44) |
| **Haiti** | 71.10(42.81to105.12) | 13.81(-31.82to81.45) |  | 18.90(8.02to34.08) | -5.98(-46.22to59.16) |  | 52.20(31.50to87.91) | 23.19(-30.45to105.38) |
| **Honduras** | 39.02(28.44to49.33) | 33.35(5.64to75.33) |  | 10.64(5.16to16.18) | 7.44(-22.19to58.29) |  | 28.37(21.03to37.06) | 46.62(13.93to96.95) |
| **Hungary** | 15.40(8.17to21.48) | 50.98(-14.79to108.94) |  | 2.27(1.60to3.06) | -41.28(-57.82to-24.23) |  | 13.13(6.21to18.87) | 107.26(-6.69to234.66) |
| **Iceland** | 10.66(7.14to15.82) | 18.55(-2.12to36.55) |  | 3.75(2.69to5.02) | -0.89(-19.87to16.39) |  | 6.90(4.16to11.50) | 32.70(5.23to70.96) |
| **India** | 51.89(30.76to63.37) | -37.80(-51.78to-16.28) |  | 6.44(3.91to8.73) | -41.81(-59.71to-11.77) |  | 45.45(26.47to56.82) | -37.19(-52.72to-15.95) |
| **Indonesia** | 42.21(27.16to48.89) | 13.67(-11.89to43.45) |  | 11.30(6.09to14.26) | 6.08(-18.05to47.32) |  | 30.91(21.08to37.13) | 16.72(-11.60to52.33) |
| **Iran (Islamic Republic of)** | 8.08(5.95to11.73) | -8.87(-22.06to10.42) |  | 1.44(1.05to1.94) | -16.60(-29.12to4.73) |  | 6.64(4.73to9.98) | -7.00(-22.94to15.27) |
| **Iraq** | 7.88(5.62to11.43) | -15.13(-34.16to7.97) |  | 1.16(0.82to1.61) | -13.00(-29.93to7.59) |  | 6.72(4.61to10.06) | -15.49(-36.58to12.28) |
| **Ireland** | 19.34(11.32to28.07) | 78.65(6.23to142.77) |  | 9.08(3.87to12.81) | 93.92(-17.63to166.18) |  | 10.26(6.57to16.66) | 67.02(22.35to161.04) |
| **Israel** | 29.48(16.12to40.26) | 103.04(19.05to170.05) |  | 16.62(6.63to21.22) | 126.70(10.82to206.98) |  | 12.87(7.70to20.42) | 78.92(21.32to153.35) |
| **Italy** | 10.39(6.89to15.59) | 40.29(4.13to69.83) |  | 2.62(1.94to3.93) | -7.07(-23.56to4.62) |  | 7.77(4.45to12.57) | 69.46(14.33to160.55) |
| **Jamaica** | 30.24(17.33to49.07) | 64.39(-0.85to152.60) |  | 6.06(3.23to9.47) | 24.79(-36.50to106.55) |  | 24.18(13.75to40.96) | 78.59(9.51to181.44) |
| **Japan** | 12.45(8.55to18.42) | 34.05(4.00to56.10) |  | 6.36(4.47to8.80) | 41.76(-4.12to68.58) |  | 6.08(3.47to10.63) | 26.83(11.04to56.12) |
| **Jordan** | 8.81(6.27to12.91) | -6.50(-28.26to15.41) |  | 2.43(1.57to3.22) | 6.10(-30.17to58.02) |  | 6.38(4.20to10.35) | -10.55(-35.97to22.07) |
| **Kazakhstan** | 8.29(5.69to11.96) | 18.07(3.93to35.89) |  | 4.25(2.77to5.83) | 15.07(-2.76to36.49) |  | 4.04(2.47to6.76) | 21.40(7.26to50.66) |
| **Kenya** | 51.41(37.45to66.63) | 17.38(-12.14to49.23) |  | 12.10(3.75to19.55) | 3.56(-36.74to59.10) |  | 39.32(28.56to53.43) | 22.40(-12.27to58.83) |
| **Kiribati** | 89.54(56.56to125.55) | -6.59(-33.19to35.20) |  | 10.99(4.68to21.05) | -29.19(-54.32to16.22) |  | 78.56(48.01to112.21) | -2.23(-31.19to40.60) |
| **Kuwait** | 9.35(5.69to13.69) | 45.05(3.40to92.80) |  | 1.30(0.93to1.78) | -1.42(-16.74to11.05) |  | 8.05(4.59to12.15) | 57.01(5.47to119.39) |
| **Kyrgyzstan** | 15.14(10.11to23.26) | -9.30(-28.49to29.08) |  | 7.40(4.55to12.27) | -21.16(-40.19to14.83) |  | 7.75(5.16to11.91) | 5.92(-17.00to52.98) |
| **Lao People's Democratic Republic** | 32.74(21.98to44.62) | -22.33(-46.10to9.06) |  | 8.47(4.65to12.49) | -33.29(-55.60to10.93) |  | 24.27(16.25to34.33) | -17.61(-47.34to22.97) |
| **Latvia** | 34.68(19.31to48.77) | 22.86(-16.13to58.59) |  | 13.81(7.34to20.36) | 12.52(-27.18to51.35) |  | 20.87(10.86to30.01) | 30.81(-8.91to70.56) |
| **Lebanon** | 8.81(5.92to14.26) | -13.56(-31.11to17.97) |  | 1.46(0.95to2.37) | -24.06(-43.11to11.57) |  | 7.35(4.84to12.11) | -11.12(-30.85to23.80) |
| **Lesotho** | 66.51(46.43to93.81) | 38.69(0.66to87.60) |  | 17.91(7.95to29.50) | 32.34(-12.58to102.83) |  | 48.60(33.03to69.10) | 41.19(1.66to95.79) |
| **Liberia** | 51.41(36.22to73.45) | -17.65(-42.16to16.04) |  | 3.43(1.82to5.56) | -34.08(-67.11to49.36) |  | 47.98(32.84to69.49) | -16.16(-41.94to22.27) |
| **Libya** | 11.09(7.57to17.16) | -5.10(-28.40to31.13) |  | 1.73(1.09to3.10) | -15.71(-39.84to29.50) |  | 9.36(5.89to14.82) | -2.84(-28.74to34.78) |
| **Lithuania** | 27.74(15.38to39.14) | 52.28(-8.74to102.97) |  | 10.38(5.70to14.28) | 27.18(-31.38to77.96) |  | 17.35(9.34to25.64) | 72.66(8.20to135.02) |
| **Luxembourg** | 12.78(8.17to19.48) | 33.18(-1.07to63.64) |  | 2.47(1.77to3.38) | -2.64(-27.69to15.67) |  | 10.31(6.11to16.52) | 46.04(6.20to96.94) |
| **Madagascar** | 39.99(29.24to54.00) | -10.91(-34.92to21.18) |  | 9.92(3.22to17.82) | -18.85(-48.80to30.80) |  | 30.07(21.50to41.53) | -7.93(-35.91to30.51) |
| **Malawi** | 38.34(29.99to47.93) | -8.17(-41.70to32.81) |  | 8.68(3.18to14.28) | -16.72(-68.67to69.29) |  | 29.66(21.38to39.61) | -5.33(-43.67to55.83) |
| **Malaysia** | 75.04(51.55to104.58) | 89.58(25.01to202.00) |  | 23.25(12.54to34.09) | 76.91(0.75to201.50) |  | 51.79(34.82to81.53) | 95.88(22.69to251.80) |
| **Maldives** | 35.03(24.58to44.22) | -28.40(-50.04to5.28) |  | 11.45(6.99to15.41) | -23.30(-60.60to54.45) |  | 23.58(16.82to30.60) | -30.65(-53.07to8.64) |
| **Mali** | 57.39(42.70to76.08) | 0.08(-26.71to37.41) |  | 4.41(2.02to6.90) | -19.80(-51.09to30.11) |  | 52.99(39.04to71.32) | 2.18(-26.03to42.02) |
| **Malta** | 17.60(11.10to26.05) | 79.53(25.33to142.37) |  | 9.70(5.59to15.18) | 119.35(39.98to175.73) |  | 7.90(4.58to13.32) | 46.81(13.19to123.34) |
| **Marshall Islands** | 75.17(51.49to106.65) | -7.53(-37.02to37.41) |  | 12.74(4.09to23.47) | -38.75(-64.46to18.66) |  | 62.43(40.96to92.15) | 3.20(-30.77to54.96) |
| **Mauritania** | 50.94(34.56to69.80) | -18.43(-41.85to12.48) |  | 3.21(1.66to5.16) | -31.35(-58.33to48.03) |  | 47.73(31.58to66.34) | -17.39(-42.30to13.90) |
| **Mauritius** | 21.17(8.89to30.08) | 262.34(49.45to494.75) |  | 4.36(1.72to6.17) | 226.30(5.80to477.21) |  | 16.81(6.98to23.97) | 273.04(63.93to519.97) |
| **Mexico** | 42.61(23.84to58.94) | 76.72(12.51to112.80) |  | 14.62(7.83to20.83) | 28.66(-14.69to55.15) |  | 27.98(14.52to40.32) | 119.57(31.13to184.38) |
| **Micronesia (Federated States of)** | 78.56(52.26to115.52) | -18.82(-48.01to22.64) |  | 10.38(4.40to17.53) | -43.51(-65.47to4.48) |  | 68.19(43.18to104.75) | -13.03(-44.37to33.86) |
| **Monaco** | 9.21(5.93to14.96) | -0.33(-10.72to10.15) |  | 1.82(1.26to2.55) | -0.07(-13.65to15.74) |  | 7.39(4.37to12.70) | -0.39(-11.82to11.65) |
| **Mongolia** | 24.10(18.70to31.81) | -19.61(-50.20to17.99) |  | 2.09(1.40to2.86) | -18.17(-41.60to17.74) |  | 22.01(16.99to29.43) | -19.74(-51.70to22.47) |
| **Montenegro** | 6.14(3.81to9.88) | 1.67(-4.33to9.06) |  | 1.59(1.10to2.18) | 0.50(-9.76to11.59) |  | 4.54(2.52to8.15) | 2.09(-4.72to10.21) |
| **Morocco** | 11.60(7.68to18.25) | 14.18(-4.99to38.99) |  | 1.88(1.29to3.40) | -11.80(-33.47to28.55) |  | 9.72(6.11to15.10) | 21.08(-1.41to48.89) |
| **Mozambique** | 57.29(42.43to77.33) | 9.00(-27.19to58.64) |  | 13.61(4.46to23.50) | -3.29(-45.45to47.36) |  | 43.68(30.64to62.06) | 13.50(-26.81to74.94) |
| **Myanmar** | 25.51(18.46to35.12) | -24.92(-46.37to4.47) |  | 6.70(3.60to9.93) | -32.89(-54.28to5.88) |  | 18.81(12.76to26.68) | -21.60(-46.36to15.12) |
| **Namibia** | 37.20(23.89to55.29) | -14.59(-38.08to19.95) |  | 9.73(4.32to14.84) | -24.09(-47.11to13.65) |  | 27.47(17.45to41.70) | -10.63(-34.15to25.92) |
| **Nauru** | 69.48(46.49to106.16) | -8.44(-32.45to24.79) |  | 10.52(4.16to18.22) | -38.37(-62.16to13.02) |  | 58.96(37.05to97.30) | 0.25(-26.35to39.52) |
| **Nepal** | 8.07(4.67to14.34) | 5.10(-4.00to13.64) |  | 1.25(0.87to1.71) | 2.89(-9.16to13.59) |  | 6.82(3.59to12.83) | 5.52(-4.83to15.34) |
| **Netherlands** | 24.51(15.22to33.79) | 93.68(8.64to153.96) |  | 3.54(2.38to4.83) | -6.14(-37.62to11.67) |  | 20.98(12.78to29.74) | 136.01(21.69to241.42) |
| **New Zealand** | 29.72(17.50to40.25) | 39.08(-5.76to63.26) |  | 18.21(9.88to23.17) | 32.45(-19.84to57.74) |  | 11.51(6.90to18.60) | 51.03(12.74to105.00) |
| **Nicaragua** | 32.01(25.44to39.99) | 34.00(6.64to68.18) |  | 6.97(3.31to10.03) | 71.30(-29.25to207.50) |  | 25.03(19.01to33.86) | 26.34(-0.42to69.63) |
| **Niger** | 52.51(37.58to71.77) | -8.93(-34.60to28.22) |  | 4.50(1.96to7.41) | -23.62(-57.30to25.09) |  | 48.01(34.05to66.11) | -7.26(-34.04to30.60) |
| **Nigeria** | 43.04(31.82to55.88) | -1.55(-22.44to27.38) |  | 3.47(1.80to5.06) | -14.40(-41.11to34.07) |  | 39.57(29.05to51.53) | -0.24(-21.98to30.50) |
| **Niue** | 53.18(36.53to72.93) | -7.79(-37.28to33.82) |  | 6.38(3.17to10.07) | -43.33(-66.69to13.99) |  | 46.80(32.14to66.07) | 0.84(-31.98to48.71) |
| **North Macedonia** | 7.00(4.69to10.69) | -8.67(-21.31to2.35) |  | 1.70(1.21to2.32) | -19.74(-33.79to2.76) |  | 5.30(3.22to8.91) | -4.44(-23.09to11.22) |
| **Northern Mariana Islands** | 138.49(97.84to171.57) | 62.53(-8.57to128.26) |  | 43.00(19.00to62.90) | 7.08(-35.44to84.15) |  | 95.49(68.84to129.83) | 111.96(-6.78to233.59) |
| **Norway** | 20.38(9.12to28.66) | 117.29(7.19to230.22) |  | 3.44(1.66to4.53) | 96.45(-14.10to185.75) |  | 16.94(7.24to24.31) | 122.07(12.79to252.90) |
| **Oman** | 83.22(59.06to102.85) | -3.37(-38.08to60.85) |  | 16.89(8.71to26.42) | -49.56(-73.86to52.40) |  | 66.33(40.70to85.18) | 26.00(-31.28to158.80) |
| **Pakistan** | 8.06(4.63to13.90) | 9.80(1.77to21.07) |  | 1.36(0.95to1.88) | 8.37(-3.82to22.41) |  | 6.69(3.47to12.53) | 10.10(0.98to22.02) |
| **Palau** | 10.23(7.43to13.25) | -1.03(-25.85to29.45) |  | 1.03(0.75to1.38) | -9.26(-26.98to11.00) |  | 9.20(6.44to12.02) | -0.01(-26.27to33.40) |
| **Palestine** | 10.55(7.27to14.25) | -22.80(-43.44to3.91) |  | 2.17(1.29to2.88) | -37.34(-59.85to-0.66) |  | 8.37(5.78to11.91) | -17.85(-38.63to10.26) |
| **Panama** | 24.45(15.58to37.02) | 60.91(14.87to127.73) |  | 3.29(2.14to4.53) | 33.68(-5.30to88.26) |  | 21.16(13.44to33.42) | 66.18(17.16to139.70) |
| **Papua New Guinea** | 71.36(41.94to106.58) | 7.31(-21.50to46.69) |  | 11.46(3.68to22.05) | -25.22(-50.43to16.52) |  | 59.90(35.56to89.67) | 17.05(-17.54to64.80) |
| **Paraguay** | 36.14(27.69to46.99) | 76.66(14.17to156.16) |  | 12.85(7.19to17.66) | 79.03(-16.18to168.67) |  | 23.28(16.23to34.53) | 75.37(21.50to169.43) |
| **Peru** | 13.56(9.93to19.08) | -49.79(-68.27to-21.89) |  | 3.23(2.29to4.72) | -70.28(-84.11to-5.12) |  | 10.32(7.00to15.27) | -35.96(-70.08to14.42) |
| **Philippines** | 30.73(22.61to37.71) | -29.24(-44.81to-4.71) |  | 12.64(7.89to15.76) | -28.51(-44.49to-3.35) |  | 18.09(13.64to24.06) | -29.74(-47.75to1.40) |
| **Poland** | 7.74(5.07to12.20) | -25.30(-39.30to10.54) |  | 1.67(1.14to3.06) | -70.17(-80.65to-23.06) |  | 6.07(3.21to10.00) | 27.55(-26.70to104.80) |
| **Portugal** | 14.44(9.65to23.78) | 68.18(18.01to120.48) |  | 4.92(3.30to8.18) | 42.81(-3.44to70.29) |  | 9.52(6.15to16.15) | 85.19(31.97to183.84) |
| **Puerto Rico** | 55.12(29.31to77.03) | 98.30(-0.96to190.10) |  | 9.16(4.11to13.28) | -15.84(-51.79to18.23) |  | 45.96(24.13to63.91) | 171.71(15.21to325.04) |
| **Qatar** | 59.25(29.79to78.05) | -19.28(-46.59to25.74) |  | 3.82(2.33to7.71) | -32.10(-56.24to10.52) |  | 55.43(26.69to73.67) | -18.21(-46.62to33.92) |
| **Republic of Korea** | 9.89(6.79to14.77) | -6.55(-22.99to10.26) |  | 4.07(2.92to5.35) | -9.25(-30.03to16.77) |  | 5.81(3.40to10.26) | -4.56(-23.16to13.97) |
| **Republic of Moldova** | 33.62(22.44to60.25) | 1.67(-23.02to46.28) |  | 22.51(13.14to42.04) | 25.72(-16.00to79.41) |  | 11.11(7.58to20.51) | -26.73(-48.44to10.92) |
| **Romania** | 11.16(7.53to16.51) | -1.57(-21.86to20.21) |  | 3.28(2.35to4.61) | -11.04(-34.09to10.03) |  | 7.87(4.77to12.51) | 3.00(-21.62to29.66) |
| **Russian Federation** | 42.77(25.31to62.40) | 46.99(-1.19to85.78) |  | 30.08(17.09to45.53) | 61.29(-3.11to111.56) |  | 12.69(7.21to18.65) | 21.47(-3.01to45.31) |
| **Rwanda** | 46.87(34.73to61.90) | -8.20(-37.72to36.24) |  | 10.75(3.60to17.67) | -25.28(-62.16to57.46) |  | 36.12(26.09to50.39) | -1.50(-38.19to55.47) |
| **Saint Kitts and Nevis** | 75.12(37.90to140.41) | 59.30(4.71to153.57) |  | 14.00(6.40to33.22) | -11.82(-44.90to54.96) |  | 61.11(30.85to112.87) | 95.42(21.29to218.02) |
| **Saint Lucia** | 67.48(39.86to110.04) | 118.95(37.50to214.19) |  | 22.30(13.27to39.69) | 54.75(1.72to126.93) |  | 45.18(24.79to74.18) | 175.33(52.15to322.37) |
| **Saint Vincent and the Grenadines** | 65.15(36.44to104.08) | 396.44(68.19to898.95) |  | 14.30(8.08to24.97) | 173.98(31.02to356.80) |  | 50.85(27.34to81.40) | 543.36(76.62to1711.75) |
| **Samoa** | 58.33(37.71to83.08) | -16.81(-42.70to23.18) |  | 7.52(3.53to12.24) | -34.97(-59.86to11.16) |  | 50.81(32.10to72.98) | -13.23(-41.34to34.57) |
| **San Marino** | 9.94(6.58to15.27) | 11.51(-4.07to36.55) |  | 2.04(1.44to2.84) | 6.05(-13.55to29.55) |  | 7.90(4.83to12.96) | 13.02(-4.31to41.55) |
| **Sao Tome and Principe** | 45.18(34.16to58.01) | -7.42(-31.60to29.40) |  | 3.27(1.74to5.11) | -14.71(-44.23to42.27) |  | 41.91(31.38to53.30) | -6.80(-32.30to29.30) |
| **Saudi Arabia** | 6.83(4.53to10.76) | -10.67(-37.61to38.82) |  | 1.42(1.00to1.98) | -22.28(-49.74to25.44) |  | 5.41(3.38to9.11) | -7.02(-35.44to42.09) |
| **Senegal** | 54.46(39.00to71.10) | -4.47(-30.10to27.64) |  | 4.00(1.90to6.24) | -19.54(-48.95to34.72) |  | 50.46(36.22to66.00) | -3.03(-29.04to32.58) |
| **Serbia** | 12.22(8.50to16.46) | 20.66(-18.77to55.19) |  | 5.96(3.70to7.84) | 26.36(-19.67to71.72) |  | 6.26(4.05to9.70) | 15.69(-21.43to58.95) |
| **Seychelles** | 24.53(18.85to31.47) | 52.16(4.12to108.62) |  | 5.98(3.42to8.25) | 26.39(-21.63to99.72) |  | 18.55(13.75to24.53) | 62.86(11.89to132.10) |
| **Sierra Leone** | 53.01(38.16to74.73) | -2.76(-30.06to36.88) |  | 4.06(1.82to6.45) | -19.11(-50.58to26.58) |  | 48.95(34.44to70.93) | -1.10(-30.22to41.50) |
| **Singapore** | 23.89(13.85to36.67) | 23.25(-16.13to73.16) |  | 16.96(8.89to26.33) | 19.71(-28.86to68.48) |  | 6.93(4.00to12.20) | 32.85(8.68to119.39) |
| **Slovakia** | 10.28(6.82to15.40) | 33.16(-4.98to68.16) |  | 2.03(1.44to2.78) | -1.20(-17.01to27.06) |  | 8.25(5.10to12.85) | 45.63(-4.24to100.92) |
| **Slovenia** | 8.91(5.75to13.58) | 9.65(-17.32to32.59) |  | 2.28(1.61to3.02) | -4.23(-32.09to17.35) |  | 6.63(3.89to10.82) | 15.39(-10.90to44.95) |
| **Solomon Islands** | 91.45(58.45to141.89) | 3.90(-25.59to46.98) |  | 15.49(4.63to32.42) | -32.92(-57.23to17.41) |  | 75.96(45.03to129.33) | 16.99(-16.89to65.78) |
| **Somalia** | 49.97(35.09to77.24) | -2.40(-33.92to39.54) |  | 13.11(4.21to23.68) | -9.16(-43.39to53.82) |  | 36.86(22.92to66.98) | 0.25(-34.88to49.80) |
| **South Africa** | 38.17(30.45to48.11) | -10.14(-20.56to2.64) |  | 14.41(9.62to18.11) | -11.96(-28.06to11.04) |  | 23.76(17.22to32.07) | -8.99(-26.99to10.22) |
| **South Sudan** | 51.70(33.72to70.38) | 0.83(-32.72to40.35) |  | 12.45(3.66to21.63) | -12.09(-50.06to56.84) |  | 39.25(27.70to54.04) | 5.76(-33.28to66.90) |
| **Spain** | 17.17(10.69to24.59) | 81.00(16.12to142.31) |  | 5.45(3.14to7.34) | 61.03(-17.95to112.72) |  | 11.73(7.10to18.23) | 92.07(27.87to193.90) |
| **Sri Lanka** | 9.22(6.89to12.05) | -7.44(-31.45to23.92) |  | 3.59(2.09to5.03) | 5.45(-27.12to45.98) |  | 5.63(3.86to8.22) | -14.13(-37.58to20.75) |
| **Sudan** | 15.50(8.18to25.56) | 40.07(-3.43to102.40) |  | 2.03(1.18to4.06) | -20.83(-60.12to120.60) |  | 13.47(6.10to22.90) | 58.47(-4.25to155.01) |
| **Suriname** | 98.69(67.83to127.78) | 52.01(2.68to115.00) |  | 13.58(8.33to19.96) | -2.80(-44.32to71.50) |  | 85.10(58.09to110.48) | 67.04(6.21to148.70) |
| **Sweden** | 23.15(13.18to32.01) | 80.63(-3.68to144.76) |  | 2.78(1.67to3.88) | -8.79(-49.56to15.50) |  | 20.37(11.01to28.71) | 108.53(5.48to200.29) |
| **Switzerland** | 15.69(9.31to22.62) | 52.67(-3.82to108.73) |  | 3.93(2.06to5.13) | 50.06(-21.86to105.50) |  | 11.75(7.13to17.77) | 53.56(1.73to119.57) |
| **Syrian Arab Republic** | 5.49(3.47to8.81) | 0.80(-9.83to13.40) |  | 1.06(0.76to1.47) | -3.31(-14.90to9.77) |  | 4.42(2.60to7.73) | 1.84(-10.18to16.36) |
| **Taiwan (Province of China)** | 38.48(21.33to69.37) | 107.04(28.81to191.08) |  | 25.33(12.40to42.94) | 112.28(11.36to197.42) |  | 13.15(7.86to28.66) | 97.63(42.44to232.36) |
| **Tajikistan** | 7.63(5.62to10.41) | 6.01(-8.85to30.08) |  | 1.64(1.18to2.23) | -0.54(-13.62to13.33) |  | 5.99(4.19to8.54) | 7.96(-10.01to38.94) |
| **Thailand** | 48.60(35.69to65.06) | -24.64(-48.74to14.72) |  | 3.35(1.73to4.87) | 51.61(-48.64to251.37) |  | 45.25(32.91to61.31) | -27.34(-52.00to17.80) |
| **Timor-Leste** | 29.46(18.25to46.63) | -19.92(-47.70to21.54) |  | 8.20(4.03to13.04) | -22.27(-47.23to18.50) |  | 21.26(13.21to34.37) | -18.98(-50.43to27.19) |
| **Togo** | 47.48(35.91to61.28) | -3.20(-27.42to27.89) |  | 3.77(1.80to5.64) | -16.40(-42.91to22.14) |  | 43.71(32.44to57.53) | -1.86(-27.12to30.10) |
| **Tokelau** | 51.25(33.19to70.86) | -8.44(-36.96to31.64) |  | 6.43(3.10to10.14) | -43.17(-65.97to10.38) |  | 44.82(28.45to62.94) | 0.35(-32.24to51.49) |
| **Tonga** | 57.40(37.98to82.06) | 5.27(-24.54to47.74) |  | 6.75(3.09to11.11) | -29.16(-56.76to20.93) |  | 50.65(32.44to73.68) | 12.56(-19.74to59.25) |
| **Trinidad and Tobago** | 50.73(30.62to77.52) | 22.24(-11.79to71.78) |  | 21.44(10.05to33.06) | 3.37(-29.83to50.78) |  | 29.29(19.31to47.50) | 41.09(0.57to95.14) |
| **Tunisia** | 9.24(6.39to14.40) | -1.58(-21.33to27.86) |  | 1.53(1.02to2.52) | -16.66(-38.20to19.00) |  | 7.70(5.11to12.25) | 2.09(-19.00to33.14) |
| **Turkey** | 8.67(6.37to12.62) | -23.46(-39.68to-1.35) |  | 2.29(1.53to2.99) | -33.37(-51.99to9.26) |  | 6.38(4.32to10.20) | -19.16(-39.97to3.80) |
| **Turkmenistan** | 10.42(7.30to15.70) | 18.38(-4.41to48.20) |  | 3.63(2.51to5.50) | 0.12(-21.41to30.17) |  | 6.79(4.54to10.94) | 31.19(3.32to77.35) |
| **Tuvalu** | 62.76(41.28to87.78) | -12.14(-38.56to24.98) |  | 9.02(3.70to14.51) | -40.62(-62.57to9.33) |  | 53.74(34.96to76.21) | -4.45(-35.17to43.81) |
| **Uganda** | 40.71(30.02to56.25) | -0.12(-27.03to37.48) |  | 10.14(3.47to17.12) | -10.17(-41.25to39.52) |  | 30.57(20.98to42.93) | 3.72(-25.90to50.87) |
| **Ukraine** | 22.87(13.69to30.30) | 75.20(24.82to134.05) |  | 14.66(8.16to19.28) | 83.30(23.03to149.00) |  | 8.21(4.65to12.11) | 62.40(18.17to121.55) |
| **United Arab Emirates** | 11.15(6.90to19.82) | 6.30(-19.56to45.53) |  | 1.88(1.12to3.71) | -20.42(-49.21to42.09) |  | 9.28(5.46to16.79) | 14.06(-15.43to64.64) |
| **United Kingdom** | 33.58(19.95to45.04) | 118.76(26.43to192.04) |  | 18.77(9.18to23.87) | 197.66(17.23to310.27) |  | 14.81(9.52to23.59) | 63.74(29.15to109.62) |
| **United Republic of Tanzania** | 48.98(36.85to66.09) | 1.61(-27.56to44.98) |  | 10.34(3.55to17.18) | -10.96(-46.99to33.00) |  | 38.64(29.11to52.03) | 5.60(-28.47to60.79) |
| **United States of America** | 26.03(17.31to35.99) | 80.95(25.24to112.62) |  | 17.87(11.78to23.00) | 54.74(7.93to82.51) |  | 8.17(4.40to12.89) | 187.41(75.64to244.69) |
| **United States Virgin Islands** | 46.33(31.09to60.02) | 114.57(24.03to208.42) |  | 16.43(10.37to24.18) | 60.36(-2.05to172.09) |  | 29.90(17.82to39.55) | 163.52(44.19to308.04) |
| **Uruguay** | 23.70(16.13to41.28) | 19.69(-5.25to58.09) |  | 6.24(4.28to11.54) | -36.21(-53.88to19.91) |  | 17.46(11.15to30.91) | 74.32(16.88to150.84) |
| **Uzbekistan** | 9.32(6.66to14.33) | 9.17(-9.32to38.64) |  | 3.19(2.26to5.08) | 8.11(-13.03to45.57) |  | 6.13(4.08to10.03) | 9.73(-8.87to42.03) |
| **Vanuatu** | 92.86(53.66to132.70) | 18.34(-16.99to72.02) |  | 11.88(5.13to20.00) | -26.97(-53.13to27.50) |  | 80.97(45.75to114.92) | 30.20(-9.52to90.63) |
| **Venezuela (Bolivarian Republic of)** | 8.56(5.28to13.63) | -3.68(-21.47to29.46) |  | 2.09(1.42to2.93) | -25.86(-46.07to16.37) |  | 6.47(3.58to11.11) | 6.64(-12.49to54.98) |
| **Viet Nam** | 28.26(10.27to39.01) | -32.80(-54.34to5.08) |  | 7.50(2.68to11.02) | -35.96(-58.62to1.32) |  | 20.76(7.97to30.39) | -31.58(-55.23to9.94) |
| **Yemen** | 9.91(6.88to15.10) | 1.59(-21.24to30.97) |  | 2.04(1.33to4.21) | -17.59(-43.72to25.48) |  | 7.88(5.18to12.31) | 8.09(-15.45to44.39) |
| **Zambia** | 45.41(34.72to58.37) | -5.22(-33.98to30.97) |  | 10.75(3.82to18.03) | -17.95(-52.77to40.13) |  | 34.66(25.37to47.53) | -0.42(-33.19to45.96) |
| **Zimbabwe** | 50.65(36.59to70.61) | 6.40(-20.30to42.13) |  | 5.96(3.04to12.46) | 10.43(-23.55to56.96) |  | 44.68(32.60to61.45) | 5.88(-21.48to42.42) |
