# Supplementary material for: Global Burden of Bacterial Skin Diseases: A Systematic Analysis Combined With Sociodemographic Index, 1990–2019
Source: Front Med (Lausanne). 2022 Apr 25;9:861115. doi: 10.3389/fmed.2022.861115 (PMC9084187; doi:10.3389/fmed.2022.861115)
Supplement: Supplementary file 7 [file Table_7.docx]

S7 Table Age-standardized Incidence and DALYs and of cellulitis (by sexes), 1990-2019.

| **Cellulitis** | | | | | |
| --- | --- | --- | --- | --- | --- |
|  | **Incidence** | |  | **DALYs (Disability-Adjusted Life Years)** | |
|  | **Male** | **Female** |  | **Male** | **Female** |
| **1990** | 624.54(586.79to662.33) | 540.66(508.39to572.87) |  | 8.10(4.83to12.41) | 7.48(4.55to11.99) |
| **1991** | 613.47(576.66to650.91) | 531.36(499.79to562.75) |  | 8.13(4.83to12.39) | 7.48(4.61to11.85) |
| **1992** | 603.85(567.09to640.86) | 523.47(492.11to554.43) |  | 8.13(4.84to12.39) | 7.48(4.58to11.80) |
| **1993** | 595.93(559.47to632.64) | 517.05(486.29to547.68) |  | 8.21(4.84to12.34) | 7.49(4.61to11.62) |
| **1994** | 589.83(553.61to626.18) | 512.17(481.71to542.49) |  | 8.21(4.78to12.40) | 7.50(4.65to11.58) |
| **1995** | 586.25(550.26to622.51) | 509.35(479.45to539.45) |  | 8.07(4.71to12.29) | 7.45(4.69to11.37) |
| **1996** | 584.31(548.78to620.04) | 507.73(477.90to537.71) |  | 7.88(4.62to11.98) | 7.39(4.67to11.20) |
| **1997** | 583.34(548.13to618.54) | 506.86(477.07to536.67) |  | 7.75(4.58to11.49) | 7.37(4.64to11.14) |
| **1998** | 583.03(548.09to618.15) | 506.41(476.55to536.04) |  | 7.72(4.57to11.28) | 7.36(4.66to10.90) |
| **1999** | 582.88(548.29to617.84) | 506.10(475.95to535.74) |  | 7.77(4.59to11.39) | 7.40(4.72to10.65) |
| **2000** | 582.99(548.73to618.05) | 506.14(475.81to535.68) |  | 7.78(4.59to11.20) | 7.40(4.69to10.48) |
| **2001** | 582.91(548.94to617.70) | 506.05(476.01to535.39) |  | 7.72(4.52to11.06) | 7.36(4.67to10.19) |
| **2002** | 583.03(549.25to617.76) | 506.15(476.29to535.25) |  | 7.69(4.50to11.01) | 7.31(4.67to9.96) |
| **2003** | 583.38(549.49to618.01) | 506.40(476.61to535.68) |  | 7.66(4.54to10.98) | 7.24(4.67to9.73) |
| **2004** | 584.03(549.60to618.69) | 506.84(476.86to536.09) |  | 7.62(4.50to10.94) | 7.25(4.61to9.62) |
| **2005** | 585.43(550.64to619.99) | 507.87(477.70to537.13) |  | 7.73(4.60to10.96) | 7.28(4.64to9.69) |
| **2006** | 588.37(553.93to622.63) | 510.39(480.28to539.16) |  | 7.63(4.50to10.87) | 7.20(4.63to9.46) |
| **2007** | 593.27(559.06to627.62) | 514.87(484.82to543.49) |  | 7.57(4.44to10.78) | 7.14(4.61to9.27) |
| **2008** | 598.59(564.48to632.65) | 519.71(489.89to548.09) |  | 7.61(4.45to10.74) | 7.12(4.56to9.06) |
| **2009** | 602.68(568.83to636.39) | 523.34(493.45to551.44) |  | 7.52(4.43to10.46) | 7.04(4.53to8.96) |
| **2010** | 604.33(570.86to637.87) | 524.48(495.03to552.26) |  | 7.52(4.45to10.41) | 7.05(4.43to8.77) |
| **2011** | 603.73(569.77to637.00) | 523.49(493.96to551.40) |  | 7.49(4.37to10.40) | 7.00(4.40to8.65) |
| **2012** | 602.77(568.92to636.08) | 522.27(492.76to550.29) |  | 7.43(4.34to10.32) | 6.89(4.34to8.49) |
| **2013** | 601.55(567.60to634.80) | 520.80(491.31to548.94) |  | 7.47(4.28to10.23) | 6.93(4.28to8.50) |
| **2014** | 599.97(566.04to633.24) | 519.07(489.58to547.28) |  | 7.49(4.23to10.10) | 6.92(4.28to8.52) |
| **2015** | 598.38(564.53to631.72) | 517.30(487.85to545.53) |  | 7.51(4.21to10.03) | 6.94(4.26to8.51) |
| **2016** | 595.84(561.77to629.25) | 514.79(485.67to542.81) |  | 7.48(4.14to9.88) | 6.88(4.27to8.30) |
| **2017** | 592.97(558.66to626.65) | 512.11(483.01to540.11) |  | 7.35(4.06to9.79) | 6.81(4.19to8.19) |
| **2018** | 590.76(556.43to624.32) | 509.88(480.48to538.27) |  | 7.30(4.00to9.90) | 6.75(4.18to8.04) |
| **2019** | 588.92(554.26to623.47) | 507.91(478.07to536.89) |  | 7.22(4.03to9.79) | 6.70(4.18to8.13) |
