# Supplementary material for: Global Burden of Bacterial Skin Diseases: A Systematic Analysis Combined With Sociodemographic Index, 1990–2019
Source: Front Med (Lausanne). 2022 Apr 25;9:861115. doi: 10.3389/fmed.2022.861115 (PMC9084187; doi:10.3389/fmed.2022.861115)
Supplement: Supplementary file 8 [file Table_8.docx]

S8 Table Age-standardized incidence and DALYs of cellulitis (by different SDI, both sexes), 1990-2019.

| **Cellulitis** | | | | | | | | | | | |
| --- | --- | --- | --- | --- | --- | --- | --- | --- | --- | --- | --- |
|  | **Incidence** | | | | |  | **DALYs (Disability-Adjusted Life Years)** | | | | |
|  | **High SDI** | **High-middle SDI** | **Middle SDI** | **Low-middle SDI** | **Low SDI** |  | **High SDI** | **High-middle SDI** | **Middle SDI** | **Low-middle SDI** | **Low SDI** |
| **1990** | 1519.41(1427.82to1605.42) | 439.41(411.24to467.16) | 302.28(281.05to323.53) | 363.02(338.92to386.74) | 341.62(318.62to364.59) |  | 6.85(5.19to10.34) | 7.48(5.29to11.10) | 6.67(4.25to8.72) | 8.98(4.62to13.46) | 9.09(3.28to13.25) |
| **1991** | 1482.30(1392.52to1567.85) | 436.48(408.90to464.06) | 287.90(267.68to308.32) | 358.68(334.84to382.21) | 320.66(299.17to342.10) |  | 6.87(5.26to10.48) | 7.56(5.32to11.12) | 6.63(4.26to8.74) | 8.97(4.55to13.67) | 9.05(3.25to13.19) |
| **1992** | 1450.56(1362.46to1533.54) | 434.32(406.87to461.87) | 292.03(271.57to312.68) | 358.04(334.25to381.67) | 320.24(298.93to341.66) |  | 6.91(5.25to10.58) | 7.71(5.41to11.24) | 6.50(4.21to8.59) | 8.92(4.48to13.61) | 9.01(3.20to13.15) |
| **1993** | 1425.87(1339.03to1505.56) | 432.04(404.77to459.53) | 288.96(269.02to309.39) | 357.49(333.75to381.01) | 319.94(298.60to341.41) |  | 6.99(5.32to10.72) | 8.09(5.68to11.84) | 6.35(4.21to8.25) | 8.80(4.46to12.99) | 8.93(3.24to13.04) |
| **1994** | 1409.50(1324.26to1487.91) | 429.51(402.47to456.90) | 293.20(273.15to313.79) | 357.14(333.48to380.73) | 319.63(298.26to341.01) |  | 7.08(5.39to10.84) | 8.31(5.84to12.17) | 6.22(4.17to8.12) | 8.67(4.52to12.79) | 8.83(3.20to12.87) |
| **1995** | 1403.00(1319.45to1480.35) | 427.08(400.23to454.43) | 294.07(274.05to314.78) | 356.98(333.42to380.72) | 319.40(298.03to340.75) |  | 7.15(5.46to10.96) | 8.13(5.71to11.74) | 6.05(4.11to7.86) | 8.55(4.53to12.41) | 8.74(3.26to12.76) |
| **1996** | 1402.43(1318.90to1479.41) | 424.43(397.79to451.49) | 291.45(271.69to312.04) | 357.02(333.58to380.66) | 319.24(297.85to340.58) |  | 7.21(5.52to11.01) | 7.81(5.48to11.18) | 5.92(4.05to7.72) | 8.46(4.48to12.17) | 8.64(3.17to12.48) |
| **1997** | 1403.23(1321.31to1479.88) | 421.78(395.34to448.42) | 292.31(272.54to312.98) | 357.26(333.61to381.20) | 319.18(297.79to340.67) |  | 7.31(5.60to11.13) | 7.47(5.23to10.59) | 5.83(4.01to7.60) | 8.51(4.51to12.20) | 8.57(3.22to12.36) |
| **1998** | 1405.89(1324.39to1481.83) | 419.11(392.69to445.79) | 293.18(273.42to313.94) | 357.61(333.77to381.70) | 319.22(297.81to340.46) |  | 7.50(5.70to11.19) | 7.33(5.18to10.40) | 5.75(3.99to7.40) | 8.47(4.50to11.97) | 8.49(3.25to12.22) |
| **1999** | 1410.54(1328.98to1486.58) | 416.49(389.95to442.89) | 294.17(274.32to315.07) | 358.03(334.28to382.24) | 319.33(297.84to340.33) |  | 7.75(5.93to11.35) | 7.43(5.25to10.63) | 5.68(3.98to7.32) | 8.39(4.48to11.79) | 8.41(3.24to11.98) |
| **2000** | 1417.78(1336.30to1494.88) | 414.37(387.97to440.36) | 295.45(275.46to316.34) | 358.41(334.46to382.79) | 319.46(298.15to340.68) |  | 8.03(6.07to11.60) | 7.45(5.25to10.61) | 5.57(3.91to7.20) | 8.28(4.44to11.57) | 8.32(3.28to11.87) |
| **2001** | 1426.83(1345.41to1503.57) | 412.88(386.44to438.64) | 296.87(276.72to317.84) | 358.77(335.00to382.99) | 319.66(298.27to340.66) |  | 8.30(6.20to11.82) | 7.32(5.18to10.52) | 5.44(3.87to6.97) | 8.11(4.40to11.31) | 8.18(3.28to11.72) |
| **2002** | 1437.00(1355.43to1513.34) | 412.20(386.01to437.91) | 301.84(281.36to323.23) | 359.15(335.54to383.27) | 319.96(298.70to341.34) |  | 8.56(6.27to11.97) | 7.23(5.16to10.36) | 5.32(3.86to6.72) | 7.92(4.35to11.04) | 8.08(3.23to11.55) |
| **2003** | 1448.84(1367.41to1525.47) | 411.71(385.45to437.41) | 303.24(282.59to324.74) | 359.56(335.88to383.57) | 320.36(298.98to341.89) |  | 8.79(6.39to12.14) | 7.19(5.16to10.32) | 5.21(3.84to6.59) | 7.71(4.25to10.61) | 7.99(3.24to11.37) |
| **2004** | 1462.40(1380.97to1540.38) | 411.11(384.90to436.68) | 304.64(283.82to326.09) | 360.07(336.30to383.98) | 320.83(299.40to342.45) |  | 9.01(6.47to12.30) | 7.15(5.14to10.20) | 5.12(3.79to6.50) | 7.59(4.26to10.37) | 7.91(3.27to11.27) |
| **2005** | 1478.20(1395.29to1556.36) | 410.39(384.45to435.72) | 302.65(281.91to323.90) | 360.77(336.89to384.65) | 321.34(300.00to343.31) |  | 9.23(6.55to12.57) | 7.27(5.21to10.32) | 5.09(3.76to6.43) | 7.62(4.28to10.44) | 7.86(3.29to11.19) |
| **2006** | 1505.03(1422.53to1582.88) | 408.99(383.27to434.00) | 303.90(283.03to325.05) | 362.04(337.99to386.05) | 322.17(300.68to343.93) |  | 9.45(6.59to12.76) | 7.06(5.12to9.97) | 5.04(3.74to6.35) | 7.38(4.25to10.02) | 7.73(3.27to10.91) |
| **2007** | 1544.88(1462.91to1622.82) | 407.32(381.75to431.91) | 308.35(287.18to329.78) | 363.97(339.93to388.21) | 323.46(301.96to345.33) |  | 9.67(6.71to12.93) | 6.97(5.03to9.95) | 4.97(3.72to6.17) | 7.18(4.20to9.70) | 7.65(3.27to10.84) |
| **2008** | 1587.46(1504.90to1666.25) | 405.44(380.01to430.13) | 309.23(288.06to330.55) | 366.16(342.16to390.67) | 328.31(306.47to350.37) |  | 9.91(6.84to13.13) | 6.99(5.07to9.94) | 4.95(3.67to6.19) | 7.06(4.15to9.41) | 7.57(3.28to10.71) |
| **2009** | 1622.82(1540.33to1702.63) | 403.67(378.51to428.27) | 310.70(289.49to331.91) | 368.23(344.35to392.40) | 329.66(307.57to351.95) |  | 10.11(6.87to13.26) | 6.88(5.00to9.85) | 4.90(3.62to6.04) | 6.77(4.06to9.03) | 7.44(3.27to10.53) |
| **2010** | 1641.47(1557.98to1722.12) | 402.58(377.75to427.18) | 311.73(290.50to332.94) | 369.82(345.84to393.84) | 330.57(308.38to353.03) |  | 10.26(6.92to13.45) | 6.98(5.03to10.02) | 4.86(3.59to5.93) | 6.64(4.09to8.82) | 7.34(3.30to10.43) |
| **2011** | 1646.11(1562.51to1726.92) | 401.94(377.19to426.41) | 313.95(292.61to335.51) | 370.99(346.87to395.01) | 331.13(309.04to353.61) |  | 10.42(6.95to13.57) | 6.93(4.91to9.90) | 4.82(3.55to5.91) | 6.50(4.03to8.59) | 7.23(3.27to10.21) |
| **2012** | 1647.91(1564.27to1728.53) | 401.52(376.85to425.83) | 315.36(293.93to336.92) | 372.08(347.64to396.42) | 331.70(309.72to354.19) |  | 10.50(6.89to13.64) | 6.94(4.80to9.84) | 4.83(3.54to5.90) | 6.14(3.79to8.06) | 7.05(3.19to10.00) |
| **2013** | 1647.68(1563.54to1728.04) | 401.07(376.51to425.18) | 317.07(295.55to338.78) | 373.11(348.53to397.69) | 332.26(310.01to354.76) |  | 10.59(6.88to13.67) | 7.03(4.78to9.82) | 4.90(3.55to5.89) | 6.09(3.81to7.91) | 7.00(3.18to9.97) |
| **2014** | 1645.83(1561.81to1725.83) | 400.43(375.93to424.41) | 318.58(297.02to340.53) | 374.11(349.19to398.93) | 332.84(310.64to355.34) |  | 10.68(6.83to13.75) | 7.10(4.75to9.85) | 4.95(3.52to5.89) | 5.98(3.80to7.75) | 6.93(3.24to9.85) |
| **2015** | 1643.28(1560.09to1722.94) | 399.49(375.06to423.81) | 319.80(298.19to341.91) | 375.08(350.08to399.95) | 333.43(311.36to355.70) |  | 10.77(6.86to13.82) | 7.18(4.73to9.80) | 4.98(3.48to5.87) | 5.96(3.79to7.71) | 6.89(3.20to9.86) |
| **2016** | 1635.98(1553.32to1715.84) | 397.34(372.93to421.61) | 320.83(299.43to342.88) | 376.37(351.70to401.48) | 334.53(312.36to357.21) |  | 10.78(6.91to13.80) | 7.15(4.63to9.71) | 4.97(3.50to5.87) | 5.91(3.77to7.59) | 6.80(3.17to9.69) |
| **2017** | 1626.79(1541.85to1707.01) | 395.14(370.55to419.05) | 322.17(300.52to344.43) | 377.51(352.81to402.56) | 335.52(312.98to358.67) |  | 10.68(6.84to13.68) | 6.97(4.51to9.43) | 4.95(3.50to5.87) | 5.88(3.77to7.54) | 6.73(3.22to9.60) |
| **2018** | 1619.54(1533.90to1703.24) | 393.98(369.28to418.37) | 323.89(302.03to346.35) | 378.69(354.01to404.08) | 335.89(313.37to358.52) |  | 10.61(6.84to13.55) | 6.87(4.48to9.46) | 4.93(3.45to5.88) | 5.85(3.73to7.51) | 6.67(3.18to9.51) |
| **2019** | 1611.70(1524.39to1695.94) | 393.24(368.49to417.64) | 326.80(304.80to349.61) | 380.41(355.40to406.00) | 336.14(313.60to358.93) |  | 10.55(6.83to13.57) | 6.82(4.43to9.33) | 4.90(3.47to5.81) | 5.82(3.71to7.42) | 6.60(3.19to9.44) |
