# Supplementary material for: Global Burden of Bacterial Skin Diseases: A Systematic Analysis Combined With Sociodemographic Index, 1990–2019
Source: Front Med (Lausanne). 2022 Apr 25;9:861115. doi: 10.3389/fmed.2022.861115 (PMC9084187; doi:10.3389/fmed.2022.861115)
Supplement: Supplementary file 9 [file Table_9.docx]

S9 Table Age-standardized Incidence and DALYs and of pyoderma (by sexes), 1990-2019.

| **Pyoderma** | | | | | |
| --- | --- | --- | --- | --- | --- |
|  | **Incidence** | |  | **DALYs (Disability-Adjusted Life Years)** | |
|  | **Male** | **Female** |  | **Male** | **Female** |
| **1990** | 14467.08(14083.18to14941.36) | 11737.47(11432.28to12106.09) |  | 24.92(13.26to33.26) | 23.23(15.16to31.98) |
| **1991** | 14477.09(14095.31to14951.92) | 11759.21(11454.49to12127.58) |  | 25.04(13.25to33.17) | 23.35(15.26to31.58) |
| **1992** | 14487.97(14107.16to14964.23) | 11782.12(11477.87to12151.03) |  | 24.82(13.25to32.52) | 23.45(15.40to31.42) |
| **1993** | 14499.46(14121.19to14976.44) | 11806.14(11500.48to12178.46) |  | 24.71(13.44to32.41) | 23.56(15.53to30.95) |
| **1994** | 14512.44(14135.90to14983.52) | 11832.22(11526.97to12208.10) |  | 24.57(13.47to32.13) | 23.56(15.80to30.72) |
| **1995** | 14527.33(14150.16to14997.43) | 11860.13(11553.24to12239.00) |  | 24.34(13.55to31.41) | 23.50(15.94to30.45) |
| **1996** | 14544.03(14165.23to15012.74) | 11890.75(11585.53to12272.33) |  | 24.25(13.35to31.28) | 23.53(15.87to30.36) |
| **1997** | 14561.89(14181.85to15033.91) | 11923.52(11618.72to12307.62) |  | 24.38(13.35to31.68) | 23.78(15.90to30.59) |
| **1998** | 14581.45(14202.45to15056.38) | 11957.36(11650.39to12341.89) |  | 24.33(13.46to31.21) | 23.95(16.09to30.54) |
| **1999** | 14604.26(14225.37to15075.92) | 11992.47(11688.95to12377.39) |  | 24.24(13.49to30.94) | 24.04(16.24to30.41) |
| **2000** | 14631.30(14246.24to15100.95) | 12027.94(11724.52to12414.95) |  | 24.09(13.40to30.68) | 24.05(16.41to30.12) |
| **2001** | 14663.11(14283.13to15133.26) | 12064.36(11759.09to12450.42) |  | 23.92(13.47to30.55) | 23.86(16.34to30.00) |
| **2002** | 14698.75(14317.62to15164.63) | 12102.33(11796.87to12487.49) |  | 23.65(13.45to29.84) | 23.63(16.31to29.63) |
| **2003** | 14737.86(14356.14to15200.60) | 12141.73(11832.25to12527.75) |  | 23.36(13.51to29.68) | 23.40(16.34to29.64) |
| **2004** | 14779.29(14398.34to15242.08) | 12182.75(11869.44to12570.06) |  | 23.17(13.53to29.30) | 23.39(16.33to29.26) |
| **2005** | 14822.09(14441.69to15287.96) | 12225.19(11909.42to12613.99) |  | 23.34(13.45to29.39) | 23.63(16.51to29.44) |
| **2006** | 14860.28(14473.68to15321.49) | 12262.68(11947.68to12651.09) |  | 22.96(13.61to29.09) | 23.16(16.63to28.60) |
| **2007** | 14892.18(14512.80to15359.69) | 12294.29(11979.52to12682.52) |  | 22.62(13.64to28.56) | 22.77(16.80to28.07) |
| **2008** | 14925.62(14552.73to15400.07) | 12326.71(12011.19to12715.52) |  | 22.39(13.63to28.41) | 22.44(16.82to27.68) |
| **2009** | 14963.52(14586.01to15440.67) | 12363.74(12045.98to12754.24) |  | 21.89(13.54to27.92) | 21.96(16.69to27.07) |
| **2010** | 15005.63(14628.35to15475.89) | 12406.95(12088.82to12796.55) |  | 21.56(13.50to27.70) | 21.75(16.82to26.71) |
| **2011** | 15052.74(14670.10to15525.88) | 12457.33(12141.09to12851.91) |  | 21.34(13.38to27.50) | 21.54(16.71to26.69) |
| **2012** | 15103.70(14716.59to15579.37) | 12511.63(12194.75to12905.10) |  | 20.98(13.43to27.22) | 21.21(16.75to26.11) |
| **2013** | 15156.04(14762.55to15633.93) | 12566.09(12249.74to12963.26) |  | 21.18(13.41to27.44) | 21.43(16.93to26.30) |
| **2014** | 15207.84(14812.52to15687.19) | 12618.21(12302.98to13020.38) |  | 21.25(13.37to27.49) | 21.47(17.14to26.35) |
| **2015** | 15258.20(14861.52to15738.23) | 12665.91(12349.71to13076.04) |  | 21.48(13.41to27.64) | 21.66(17.19to26.56) |
| **2016** | 15308.04(14911.05to15796.16) | 12710.20(12393.14to13120.07) |  | 21.53(13.54to27.87) | 21.53(17.24to26.22) |
| **2017** | 15353.15(14947.34to15844.93) | 12750.26(12426.81to13157.79) |  | 21.53(13.60to27.88) | 21.48(17.01to26.65) |
| **2018** | 15401.29(14997.50to15894.81) | 12793.64(12466.41to13206.98) |  | 21.57(13.56to27.72) | 21.43(16.89to26.49) |
| **2019** | 15459.49(15052.81to15951.29) | 12846.10(12513.10to13255.96) |  | 21.60(13.49to27.92) | 21.41(16.94to26.36) |
