# Supplementary material for: Global Burden of Bacterial Skin Diseases: A Systematic Analysis Combined With Sociodemographic Index, 1990–2019
Source: Front Med (Lausanne). 2022 Apr 25;9:861115. doi: 10.3389/fmed.2022.861115 (PMC9084187; doi:10.3389/fmed.2022.861115)
Supplement: Supplementary file 10 [file Table_10.docx]

S10 Table Age-standardized incidence and DALYs of pyoderma (by different SDI, both sexes), 1990-2019.

| **Pyoderma** | | | | | | | | | | | |
| --- | --- | --- | --- | --- | --- | --- | --- | --- | --- | --- | --- |
|  | **Incidence** | | | | |  | **DALYs (Disability-Adjusted Life Years)** | | | | |
|  | **High SDI** | **High-middle SDI** | **Middle SDI** | **Low-middle SDI** | **Low SDI** |  | **High SDI** | **High-middle SDI** | **Middle SDI** | **Low-middle SDI** | **Low SDI** |
| **1990** | 14403.98(14000.15to14834.90) | 10603.38(10318.42to10963.85) | 10608.01(10331.09to10963.01) | 17297.35(16844.45to17868.27) | 16906.25(16443.91to17468.53) |  | 5.78(3.66to9.75) | 12.79(9.40to16.33) | 23.31(16.18to27.90) | 49.02(27.28to63.49) | 40.16(32.11to53.36) |
| **1991** | 14328.68(13941.46to14762.05) | 10611.20(10326.65to10968.42) | 10037.45(9772.83to10370.77) | 17159.37(16703.11to17702.82) | 15880.55(15442.90to16408.21) |  | 5.87(3.75to9.81) | 12.73(9.11to16.32) | 23.38(16.11to28.06) | 48.77(27.54to62.12) | 40.08(32.03to52.75) |
| **1992** | 14259.19(13867.83to14694.27) | 10621.22(10341.11to10978.58) | 10186.71(9917.67to10524.21) | 17179.83(16725.50to17734.44) | 15876.94(15436.28to16403.89) |  | 5.97(3.85to9.92) | 12.75(9.41to16.31) | 23.08(16.12to27.58) | 48.66(27.31to61.62) | 40.08(32.01to52.32) |
| **1993** | 14194.65(13810.16to14628.94) | 10627.33(10347.54to10987.77) | 10141.22(9874.80to10477.66) | 17198.64(16744.03to17756.97) | 15878.42(15438.15to16403.75) |  | 6.09(3.96to10.10) | 12.66(9.50to16.18) | 22.81(16.04to27.25) | 48.27(27.12to61.18) | 39.93(32.07to52.20) |
| **1994** | 14136.25(13753.13to14570.89) | 10630.68(10348.96to10987.53) | 10291.31(10020.78to10628.81) | 17218.36(16762.06to17782.75) | 15878.95(15441.86to16404.90) |  | 6.22(4.08to10.22) | 12.46(9.52to15.92) | 21.85(15.66to26.04) | 47.82(27.01to60.46) | 39.62(31.91to51.07) |
| **1995** | 14087.95(13706.35to14512.72) | 10633.19(10352.45to10992.43) | 10344.42(10069.69to10678.45) | 17236.21(16780.46to17806.21) | 15878.95(15441.78to16417.41) |  | 5.70(3.58to9.70) | 12.34(9.34to16.03) | 21.59(15.53to25.70) | 49.08(27.59to64.73) | 40.29(32.13to54.04) |
| **1996** | 14050.73(13669.49to14481.32) | 10631.53(10352.25to10985.88) | 10298.17(10024.47to10636.13) | 17254.72(16797.40to17816.80) | 15883.00(15448.86to16414.49) |  | 6.57(4.35to10.56) | 12.89(9.37to16.48) | 22.52(15.97to26.89) | 48.45(27.08to61.10) | 39.47(31.99to50.07) |
| **1997** | 14022.69(13639.56to14447.18) | 10625.96(10348.72to10969.67) | 10350.59(10075.54to10691.11) | 17278.43(16821.19to17840.08) | 15894.84(15456.60to16424.64) |  | 6.82(4.54to10.81) | 12.26(9.29to15.99) | 21.77(15.59to26.00) | 48.49(27.05to60.49) | 39.51(32.20to49.89) |
| **1998** | 13998.30(13611.42to14417.38) | 10617.82(10342.85to10960.52) | 10402.72(10127.18to10743.26) | 17304.72(16848.11to17866.33) | 15913.79(15473.22to16441.78) |  | 7.18(4.87to11.19) | 12.21(9.30to15.86) | 22.13(15.82to26.29) | 48.16(26.74to58.96) | 39.38(31.98to49.10) |
| **1999** | 13974.09(13588.73to14392.05) | 10610.29(10337.17to10949.00) | 10455.41(10180.46to10796.81) | 17333.66(16875.94to17889.82) | 15935.51(15503.56to16461.91) |  | 6.38(4.16to10.36) | 12.18(9.22to15.85) | 21.00(15.56to24.96) | 47.75(26.62to59.65) | 39.39(31.65to50.24) |
| **2000** | 13947.64(13565.76to14360.14) | 10608.07(10336.43to10940.03) | 10510.57(10236.18to10852.99) | 17361.63(16907.86to17911.56) | 15955.29(15527.34to16484.34) |  | 7.50(5.10to11.55) | 12.30(9.40to16.03) | 21.35(15.52to25.38) | 47.79(26.88to58.07) | 39.30(31.89to48.77) |
| **2001** | 13914.02(13534.77to14324.74) | 10610.63(10342.96to10946.33) | 10565.78(10290.24to10908.97) | 17394.23(16943.26to17944.90) | 15976.02(15540.45to16501.99) |  | 7.82(5.32to11.91) | 12.03(9.17to15.65) | 19.94(15.20to23.81) | 47.35(26.83to57.45) | 39.10(31.92to48.37) |
| **2002** | 13878.44(13503.80to14285.60) | 10617.13(10349.87to10956.90) | 10719.68(10444.79to11068.03) | 17435.27(16985.24to17987.04) | 16001.85(15566.01to16530.94) |  | 8.32(5.70to12.52) | 11.68(9.09to15.28) | 20.47(15.25to24.41) | 45.87(26.51to54.63) | 38.92(31.43to47.85) |
| **2003** | 13843.35(13474.14to14246.61) | 10624.41(10354.60to10963.76) | 10775.74(10497.90to11125.49) | 17479.43(17032.32to18035.85) | 16032.37(15598.76to16575.94) |  | 8.08(5.50to12.22) | 11.27(8.87to14.82) | 19.48(15.00to23.32) | 46.75(26.65to55.83) | 39.02(31.52to47.99) |
| **2004** | 13808.17(13441.48to14210.51) | 10630.83(10357.46to10975.11) | 10832.44(10553.77to11183.65) | 17524.83(17075.32to18085.48) | 16064.79(15629.39to16618.06) |  | 8.48(5.78to12.73) | 11.41(8.95to15.04) | 19.15(14.93to23.01) | 45.36(26.36to53.72) | 38.76(31.50to47.57) |
| **2005** | 13776.41(13412.12to14176.73) | 10635.59(10363.35to10981.75) | 10792.28(10515.06to11142.68) | 17569.26(17115.41to18133.48) | 16095.82(15651.85to16653.68) |  | 8.61(5.85to12.85) | 11.21(8.82to14.80) | 18.71(14.65to22.45) | 46.01(26.45to54.86) | 38.94(31.51to47.60) |
| **2006** | 13745.50(13383.07to14141.10) | 10623.79(10351.91to10968.45) | 10842.55(10560.89to11192.92) | 17613.21(17165.15to18174.27) | 16131.32(15691.32to16691.06) |  | 8.83(5.92to13.14) | 10.82(8.28to14.50) | 19.03(14.84to22.84) | 43.34(26.39to51.26) | 38.08(31.27to46.26) |
| **2007** | 13716.63(13358.38to14109.29) | 10593.49(10326.19to10931.39) | 10981.69(10697.68to11333.12) | 17660.53(17212.71to18214.55) | 16173.24(15728.23to16718.94) |  | 8.73(5.88to13.02) | 11.04(8.53to14.59) | 18.33(14.54to22.13) | 44.56(26.47to53.01) | 38.47(31.29to46.85) |
| **2008** | 13690.47(13336.65to14080.08) | 10557.11(10289.74to10895.02) | 11024.01(10743.81to11380.06) | 17709.01(17258.39to18267.45) | 16364.28(15914.53to16906.35) |  | 8.93(5.96to13.27) | 10.90(8.39to14.53) | 18.43(14.48to22.16) | 42.21(26.33to50.21) | 37.68(30.89to45.62) |
| **2009** | 13664.42(13312.86to14055.93) | 10525.75(10258.03to10860.88) | 11084.62(10804.91to11441.46) | 17755.98(17306.35to18307.06) | 16406.57(15956.34to16955.74) |  | 9.21(6.04to13.73) | 11.00(8.19to14.80) | 17.78(14.31to21.78) | 38.08(25.70to45.15) | 35.77(29.40to42.86) |
| **2010** | 13642.32(13290.18to14030.72) | 10511.45(10244.58to10849.28) | 11129.82(10851.30to11488.24) | 17799.41(17348.41to18353.15) | 16440.33(15991.16to16994.38) |  | 9.02(5.99to13.38) | 10.73(8.13to14.40) | 17.99(14.37to21.93) | 40.27(25.75to47.37) | 36.92(30.41to44.82) |
| **2011** | 13622.88(13272.68to14013.86) | 10512.84(10243.41to10848.21) | 11202.38(10922.28to11560.70) | 17842.25(17386.33to18399.83) | 16468.61(16017.97to17021.27) |  | 9.13(6.03to13.61) | 10.78(8.16to14.47) | 17.62(14.23to21.48) | 39.08(25.79to46.24) | 36.31(29.86to43.87) |
| **2012** | 13611.47(13262.22to14008.70) | 10521.34(10253.26to10858.12) | 11254.51(10969.93to11613.37) | 17887.71(17428.04to18458.31) | 16498.03(16050.70to17054.24) |  | 9.53(6.04to14.10) | 10.90(8.16to14.65) | 18.07(14.26to22.17) | 36.73(25.14to43.58) | 34.93(28.97to42.09) |
| **2013** | 13606.68(13257.17to13998.83) | 10532.57(10264.73to10869.27) | 11315.77(11028.93to11672.87) | 17932.20(17465.70to18504.79) | 16526.52(16084.11to17084.32) |  | 9.37(6.01to14.00) | 11.35(8.32to15.31) | 17.66(14.29to21.68) | 36.78(25.02to43.52) | 34.90(28.95to42.16) |
| **2014** | 13606.57(13252.19to14002.07) | 10543.87(10277.54to10880.91) | 11373.73(11085.93to11735.71) | 17972.60(17502.91to18534.74) | 16553.21(16111.51to17112.69) |  | 9.31(6.03to13.89) | 11.72(8.24to15.61) | 17.85(14.25to21.92) | 36.43(25.56to43.26) | 34.90(28.75to41.92) |
| **2015** | 13610.42(13255.15to14009.47) | 10559.00(10292.22to10895.44) | 11428.16(11140.13to11791.89) | 18006.96(17537.56to18577.89) | 16575.16(16130.66to17141.95) |  | 9.45(6.00to14.02) | 11.18(8.25to15.08) | 18.17(14.28to22.44) | 36.38(24.77to43.32) | 34.81(28.88to42.00) |
| **2016** | 13623.04(13259.25to14025.49) | 10578.62(10309.35to10921.45) | 11476.39(11182.90to11843.18) | 18039.48(17571.02to18610.60) | 16601.16(16158.47to17163.18) |  | 9.58(6.07to14.15) | 11.56(8.28to15.51) | 18.26(14.24to22.31) | 36.59(24.91to43.40) | 34.80(28.71to41.70) |
| **2017** | 13640.21(13271.92to14055.98) | 10593.35(10319.14to10945.02) | 11529.56(11229.39to11910.13) | 18064.67(17608.10to18649.28) | 16620.79(16173.59to17187.61) |  | 9.55(6.06to14.10) | 11.63(8.23to15.58) | 18.17(14.24to22.19) | 36.57(24.92to43.68) | 34.72(28.75to41.84) |
| **2018** | 13653.57(13287.29to14058.80) | 10603.60(10330.65to10954.81) | 11580.41(11279.55to11954.15) | 18084.79(17627.29to18660.23) | 16628.68(16185.68to17194.40) |  | 9.54(6.13to14.11) | 11.64(8.20to15.51) | 18.19(14.20to22.18) | 36.34(24.88to43.95) | 34.36(28.47to41.47) |
| **2019** | 13664.82(13279.56to14062.69) | 10617.45(10343.26to10966.78) | 11647.34(11348.28to12014.66) | 18108.69(17636.38to18682.22) | 16636.19(16189.18to17199.82) |  | 9.54(6.13to14.11) | 11.66(8.30to15.55) | 18.17(13.99to22.37) | 36.41(24.56to43.57) | 34.61(28.52to41.42) |
